# Biomimetic Sequence-Templating Approach toward a Multiscale Modulation of Chromogenic Polymer Properties

**DOI:** 10.1021/acs.macromol.3c00403

**Published:** 2023-06-15

**Authors:** Yuyao Kuang, Ze-Fan Yao, Sujeung Lim, Catherine Ngo, Megan Alma Rocha, Dmitry A. Fishman, Herdeline Ann M. Ardoña

**Affiliations:** † Department of Chemical and Biomolecular Engineering, Samueli School of Engineering, University of California, Irvine, Irvine, California 92697, United States; ‡ Department of Biomedical Engineering, Samueli School of Engineering, University of California, Irvine, Irvine, California 92697, United States; § Department of Chemistry, School of Physical Sciences, University of California, Irvine, Irvine, California 92697, United States; ∥ Sue & Bill Gross Stem Cell Research Center, University of California, Irvine, Irvine, California 92697, United States

## Abstract

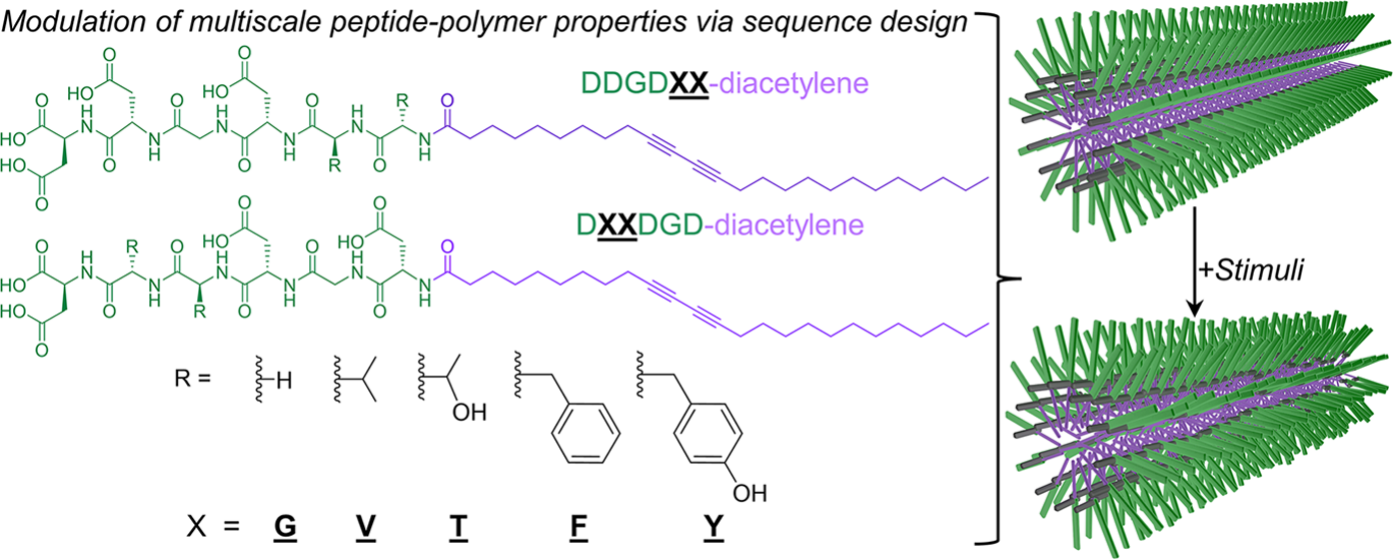

Precision control via molecular structure over adaptive conjugated polymer properties in aqueous environments is critical for realizing their biomedical applications. Here, we unravel the dependence of amphiphilic peptide-polydiacetylene (PDA) conjugate properties on the characteristic steric and hydrophobic contributions within peptide segments that serve as a biomimetic template for diacetylene polymerization in water. We investigated the functional impacts of molecular volume and polarity changes brought by dipeptide substitution domains on the following peptide-PDA material properties at multiple length scales: supramolecular assembly behavior, chain conformation-dependent photophysical properties, cell-material interfacing, and for the first time, bulk electrical properties of their films processed in water. A library of peptide-PDAs with systematically varied sequences show that the contributions of steric effects predominantly influence the electronic structure and resulting trends in photophysical properties, while the interplay between size and hydrophobicity of individual residues becomes more significant for higher-order assemblies affecting bulk properties. This work demonstrates sequence-tunable molecular volume and polarity as synthetic handles to rationally modulate PDA material properties across length scales, providing insights into the programmability of biomimetic conjugated polymers with adaptive functionalities.

## Introduction

**Figure 1:**
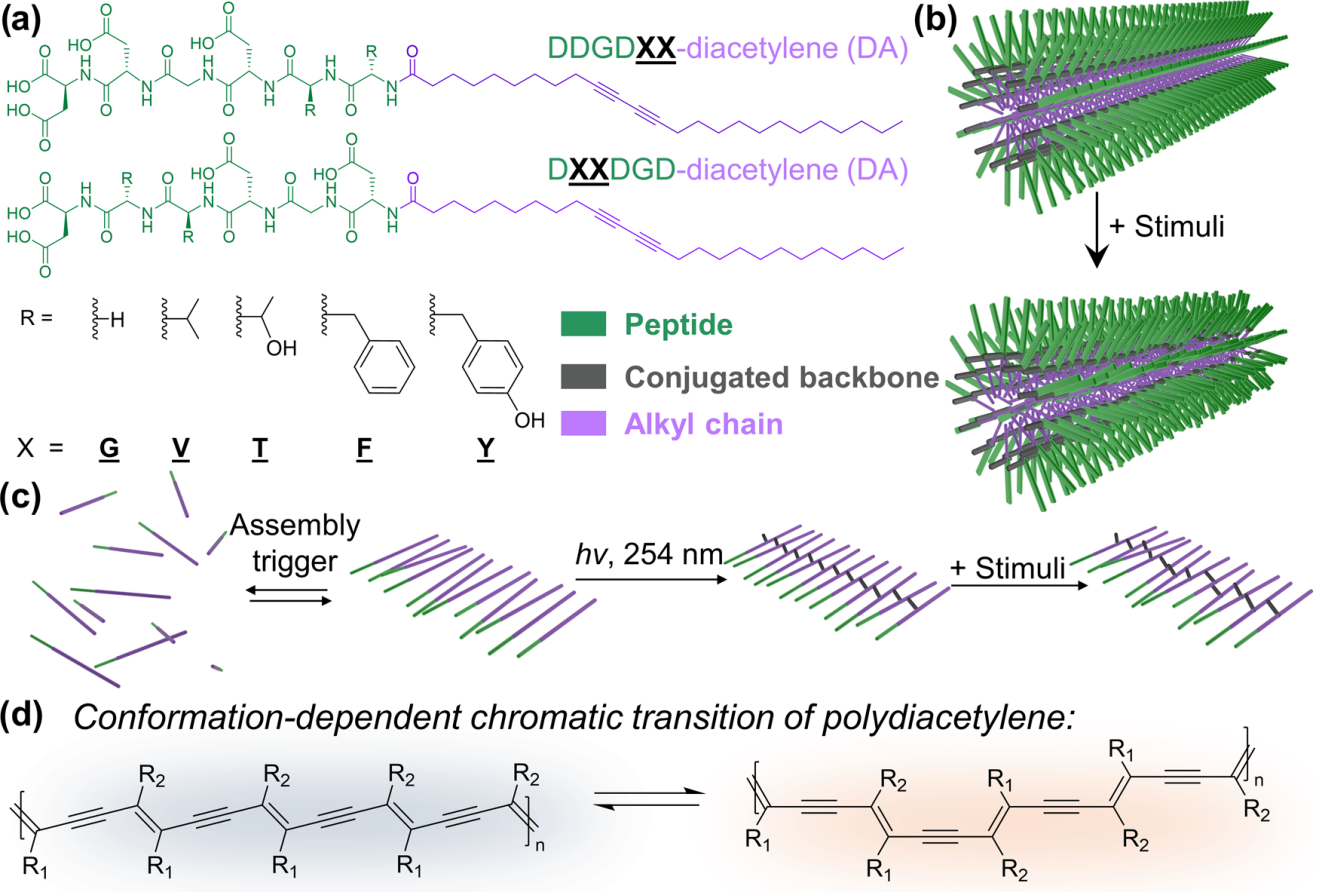
Systematically modulating residue size and hydrophobicity within peptide segments that serve as a biomimetic template for DA polymerization. (a) Molecular structures of the peptide-DA monomers used in this study (DDGD**XX**-DA and D**XX**DGD-DA, X = G, V, T, F, and Y). (b) Schematic representation of the micellar 1-D assemblies formed by peptide-PDAs, as established in the literature for many amphiphilic structures. Green, purple, and gray represent peptide moieties, DA-bearing alkyl chains, and conjugated backbones, respectively. (c) Polymerization of DA, planar structures, and torsional impact of charge repulsion on the PDA backbone adapted by PDA during (d) major chromatic transitions.

The chromatic transitions of polydiacetylene (PDA), which have been reported to be highly dependent on the conformation of conjugated backbone and sensitive to external stimuli, have received considerable interest in the fields of biosensing and imaging.^1,2^ To successfully form PDAs, diacetylene (DA) monomers undergo a UV- or γ-irradiation-induced topotactic polymerization via 1,4-addition to generate a π-conjugated ene–yne backbone only if the geometric requirements of ∼5 Å distance and 45° of tilt angles between DA monomers are satisfied (Figure 1).^3,4^ The conformation of the resulting π-conjugated polymer, and thus the corresponding electronic structure, lead to excitonic absorptions that are primarily associated with either a planar, nonfluorescent blue phase (λ_max_ ≈ 620 nm) or a nonplanar, fluorescent red phase (λ_max_ ≈ 540 nm).^5−7^ The formation of a specific phase has been established to be dependent on the inherent planarity of the electronic structure along the ene–yne chain that results in distinct chromatic phases.^8,9^ With external stimuli, torsional angles larger than 30° within the PDA conjugated backbone will lead to a bathochromic shift of the main absorption peak.^7,10−12^ There are other known chromatic phases correlated with distinct electronic structures that can be present in a PDA absorption spectrum, such as the yellow phase (λ_max_ ≈ 470 nm) or purple phase (λ_max_ ≈ 590 nm).^7^ However, PDA chromogenicity due to conformational changes is most commonly attributed to the blue-to-red phase transition. Due to the structural dependence of their optical and electronic properties, PDAs have been utilized as a colorimetric indicator of environmental conditions, such as small-molecule binding, mechanical force, heat, or solvent, for multiple sensing applications.^13−16^ As a consequence of the π-conjugation along the polymer backbone, several PDA-based materials also achieve excellent charge transport properties through control over polymerization routes and the resulting supramolecular structures.^17−20^ To tune the chromatic transitions and electronic structure of the resulting PDAs toward desired functionalities, different templating moieties can be appended to DA monomers that serve as a chemical handle for influencing the structural outcomes of topochemical polymerization.^13,14,21^

To date, self-assembled biomolecules such as peptides are among the DA templating groups shown to successfully drive and influence the photopolymerization and chromogenic transitions of PDAs.^9,22^ Noncovalent interactions within peptide moieties such as hydrogen bonding, electrostatic interactions, and π–π interactions can be programmed by introducing specific amino acids in peptide segments to support self-assemblies that satisfy the geometric requirements of topotactic DA polymerization.^23^ Taking advantage of peptidic supramolecular interactions to template the assembly of DAs not only offers a facile synthetic approach to form PDAs under aqueous conditions, but also enables a way to rationally influence PDA structure and properties via peptide sequence engineering. As such, the hierarchical nature of peptide assembly offers an advantage in probing how molecular-level changes in DA monomers impact PDA properties via the torsion-induced conformational changes at the chain-level and macroscale (bulk) ordering.^24−32^ Previous reports demonstrated the impact of DA proximity to the peptide groups and differentiated the impact of amino acid steric effect vs charge on the morphology and absorption of PDA assemblies.^33−35^ Although molecular polarity (affecting hydrophobic interactions) and residue size (imparting steric effects) are essential for the self-assembly biomolecules, the interplay between these factors is not well understood for PDAs and peptide-PDA conjugate systems. Moreover, the progression of the impact of individual residue substitution to bulk property changes for PDAs remains elusive. Considering the widespread use of PDA-based materials in various biomedical applications nowadays,^36−38^ there is a critical need for more studies that shed light on how to control material function based on the molecular design of biologically relevant templating groups for PDAs.^34,39−44^

In this study, we contribute toward addressing the knowledge gap in predicting the structural influence of the templating groups on PDA properties across length scales by molecularly controlling the properties of peptide moieties through steric effects and hydrophobic interactions (Figure 1). In particular, the impact of these molecular characteristics on DA monomer assembly and the resulting polymer conformation were mapped to photophysical and electrical properties of adaptive PDA materials templated by the biomimetic interactions between the peptide segments. We also explored how the subtle substitutions of peptide segments significantly impact the hierarchical structures and the polymerized ensembles within films that are interfaceable with human fibroblasts. Overall, this study provides new molecular-level insights into rationally designing the functionality of adaptive PDA materials under aqueous or physiologically relevant conditions by exploiting steric and hydrophobic contributions in biomimetic template groups such as peptides. These insights contribute significantly to the design principles that enable the modulation of self-assembly outcomes and specific functionalities for peptide-conjugated polymers.

## Results and Discussion

### Peptide-PDA Design

The sequence composition of oligopeptides with fixed lengths is systematically varied herein to understand how steric effects and individual residue hydrophobicity impact PDA properties across length scales. We chose 10,12-pentacosadiynoic acid (PCDA) as a commercially available PDA precursor that has been previously reported for use in sensing or tissue engineering applications.^11,16,45−47^ Here, peptide structure control was utilized to modulate supramolecular interactions between monomers and the conformation that they adapt after polymerization under aqueous environments. To do so, we prepared a library of amphiphilic DA monomers appended to a hexapeptide segment (with a dipeptide substitution domain) with comparable length as the aliphatic segments of PCDA: DDGD**XX**- and D**XX**DGD- (X = G, V, T, F, and Y; sequence presented as C → N). The dipeptide substitution domain (i.e., denoted as **XX**) is implemented at two different positions within the hexapeptide: (i) closer to the aliphatic chain or (ii) closer to the periphery (C-terminus) of the peptide region. We varied the position of the dipeptide substitution domains to assess the positional dependence of the perturbations in sterics and polarity afforded by each amino acid variation. Three aspartic acid (D) units per hexapeptide segment, with glycine (G) as a spacer between the 2nd and 3rd D positions and at least one D in the C-terminus, are also included as ionizable residues to improve aqueous solubility and to generate a pH-responsive template that can easily induce PDA conformational changes due to electrostatic repulsion between the carboxylates of D units when the pH > p*K*_a_ of the monomer. When pH < apparent p*K*_a_, protonated aspartic acids support hydrogen bonding along with other supramolecular interactions that guide the topochemical polymerization of PDAs. Hence, all peptide-PDAs here are assembled at pH 2, after which these aqueous suspensions were irradiated with 254 nm UV irradiation to initiate the polymerization process. One aspartic acid unit at the C*-*terminus of all monomers is kept constant throughout the library as the unaltered position at the polar end of the amphiphilic region. The synthesis and characterization data for these peptide-DA monomers and peptide-PDA polymers can be found in the Supporting Information (Figures S1–S42 and Table S1).

Through molecular variations in the peptidic template region of the amphiphiles, we sought to understand the distinct contributions of hydrophobicity and steric effects on conformations adapted by the DA assemblies and PDA nanostructures, as well as their resulting properties. For example, hexapeptides with divaline (VV) vs dithreonine (TT) domains have similar molecular volume, but the molecular polarity of VV-containing moiety is lower than the TT-containing analog. The same rationale goes for comparing diphenylalanine (FF) and dityrosine (YY) substitutions. As a point of reference, we used a diglycine (GG) substitution to generate a sequence with the lowest molecular volume but high molecular polarity value within the series. We can also consider that the molecular volume of TT and YY domains are larger than that of GG-, but all three are inherently more polar and less hydrophobic than the monomers with VV and FF domains. Since peptide-induced torsional impacts on the PDA backbone can result in measurable chromatic transitions, we utilize the photophysical properties of peptide-PDAs as a probe for monitoring the conformational changes imparted by the amino acid substitution across the peptide-polymer library studied herein.

**Figure 2:**
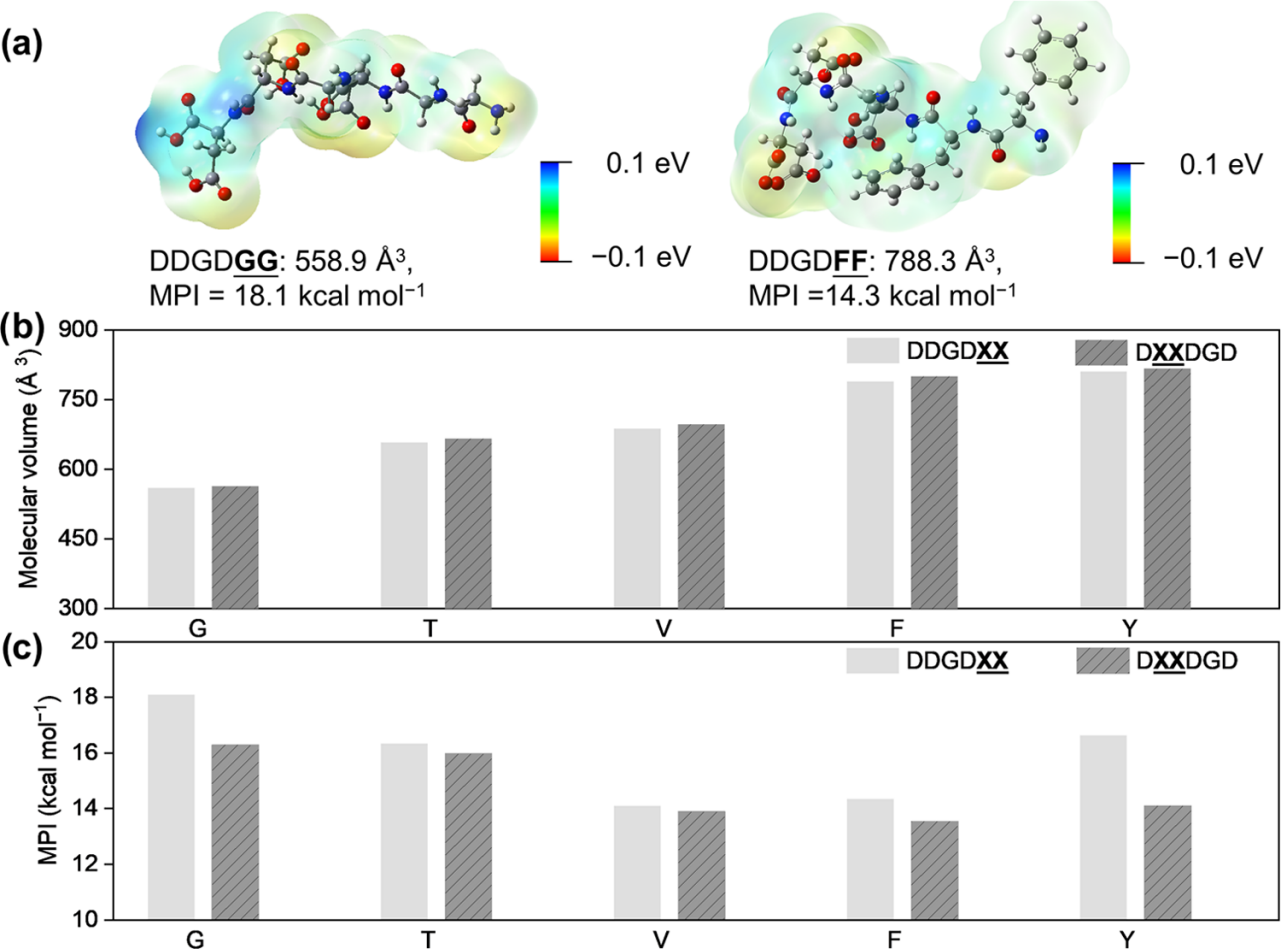
Quantitative analysis of molecular volume and polarity differences among the peptidic moieties used in this study. (a) Electrostatic potential surface maps for two representative peptides within our library: DDGD**GG** and DDGD**FF**. Plots of (b) molecular volume and (c) MPI for 10 peptides used as polar templating segments for the amphiphilic DA monomers.

To quantitatively compare the size and polarity differences of peptide segments, we calculated the molecular volume and molecular polarity index (MPI)^48−50^ for a single amino acid, dipeptides, hexapeptide segments, and peptide-DA monomers (Figures 2 and S43–S45; see the Supporting Information for a detailed description of molecular simulations). Unlike one of the conventional methods for quantifying hydrophobicity differences called grand average of hydropathy, which is based on the individual residue hydropathy and disregards the order of amino acids within a sequence,^51^ the quantitative analysis used in this study was performed using geometries optimized with density functional theory. Here, we focus on comparing the differences between the molecular volume and MPI values for energy-minimized peptide segments (Figure 2b,c) rather than the peptide-DA (Figures S43c and S44c) to emphasize the characteristic differences that the peptide segments impart on DA-bearing monomers. We hypothesize that the systematic variation within the substitution domains will allow us to probe the influence of the following supramolecular interactions between DA assemblies/PDA chains pre- and post-polymerization: (i) steric repulsion of bulky residues that could impact the distance of DA monomers and (ii) changes in hydrophobic interactions, intermolecular hydrogen bonding, and solvent–peptide segment interactions due to varying MPI values among the monomers.

### Formation of Peptide-PDA 1-D Nanostructures

**Figure 3:**
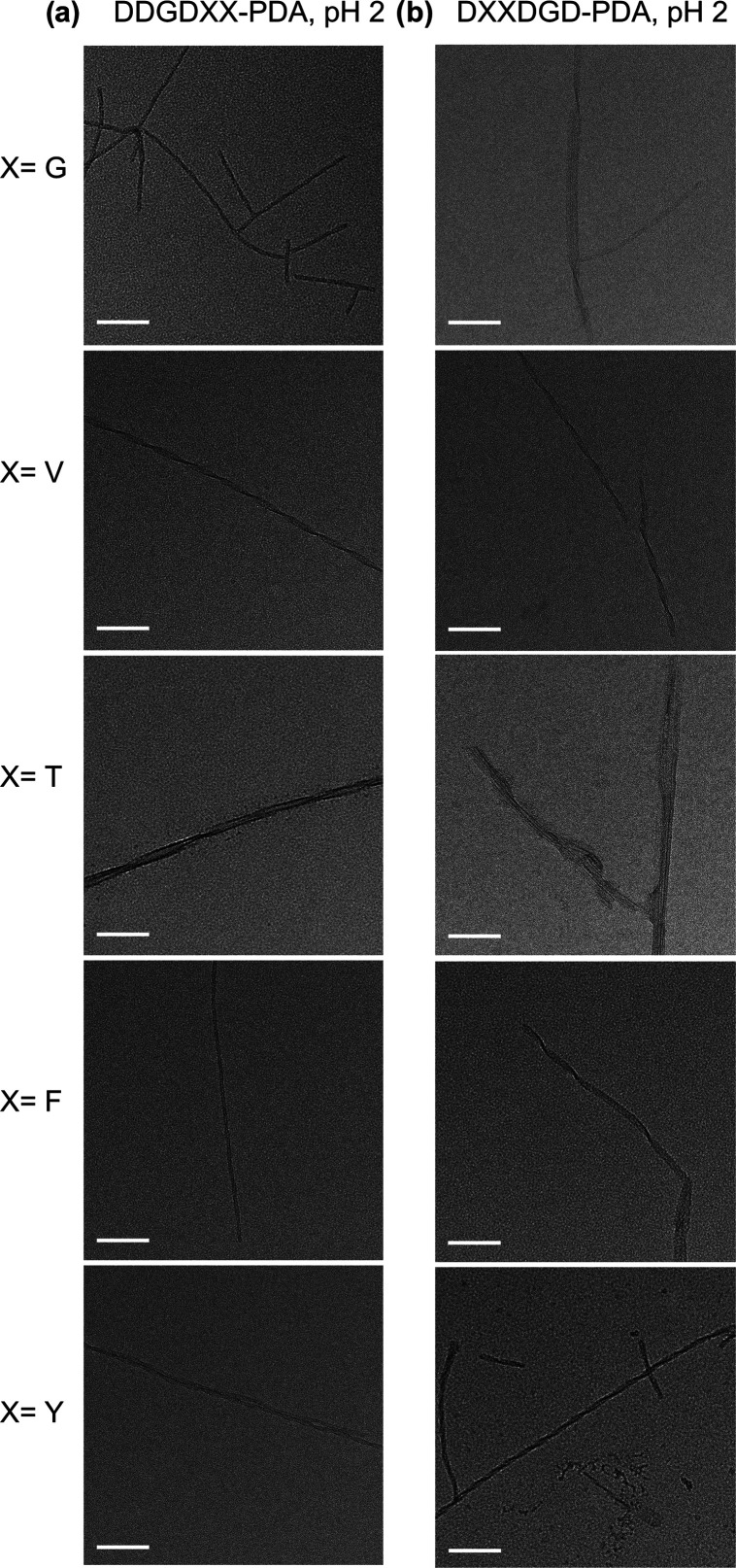
Representative TEM images of 1 mM aqueous solutions of (a) DDGD**XX**-PDA and (b) D**XX**DGD-PDA (**X** = G, V, T, F, and Y) nanostructures polymerized under 254 nm UV irradiation at pH 2. Scale bar = 100 nm.

To maximize the degree of polymerization that can be afforded by each peptidic monomer and standardize the length of UV irradiation time needed by all monomers within our library, we monitored the evolution of absorption peaks for 1 mM acidic solution of DDGD**XX**-PDA and D**XX**DGD-PDA (X = G, V, T, F, and Y) over a course of 40 min. The increase in the degree of polymerization for peptide-PDAs becomes insignificant after ∼30 min as shown in the absorption spectra (Figures S46–S48). We observed a progressive predominance of peak signatures for red phase PDAs (λ_max_ ∼540 nm) as the UV exposure increases, which is consistent with the observation for other reports on alkyl PDAs.^52,53^ The results of this absorption-based kinetic monitoring of PDA formation suggest that the degree of polymerization of our peptide-PDAs at pH 2 can be saturated within 40 min of exposure to 254 nm irradiation. In addition, the SEC characterization confirms that all samples were polymerized within the range of ∼10^5^ to ∼10^6^ Da (Figures S41 and S42 and Table S1), which is consistent with literature reports.^54,55^ A concentration-driven partial aggregation of peptide-PDAs at pH 10 condition and sample-column interaction may still exist, as indicated by the relatively short retention time and broad peak. With this, all subsequent experiments use polymer solutions resulting from peptide-DAs that are UV-irradiated with 254 nm for 40 min. From these samples, we visualized the resulting nanostructure morphologies formed by the resulting peptide-PDAs using transmission electron microscopy (TEM). All unpolymerized assemblies under acidic conditions, as well as the peptide-polymer conjugates in acidic and basic environments, formed 1-D nanostructures with fiber-like morphologies (∼5 to 16 nm) similar to other peptide amphiphiles (Figures 3, S49, and S50).^56^ Although no apparent trend changes in nanoscale morphology were observed across the sequence library or even between acidic and basic conditions, other than varying degrees of apparent twists, polydispersity, and aspect ratios, this does not eliminate the influence of peptide sequence in chain conformation and how the sequence impacts PDA properties at other length scales.

### Steric Effects within Peptide Segments Predominantly Influence the Photophysical Properties of Peptide-PDAs

**Figure 4:**
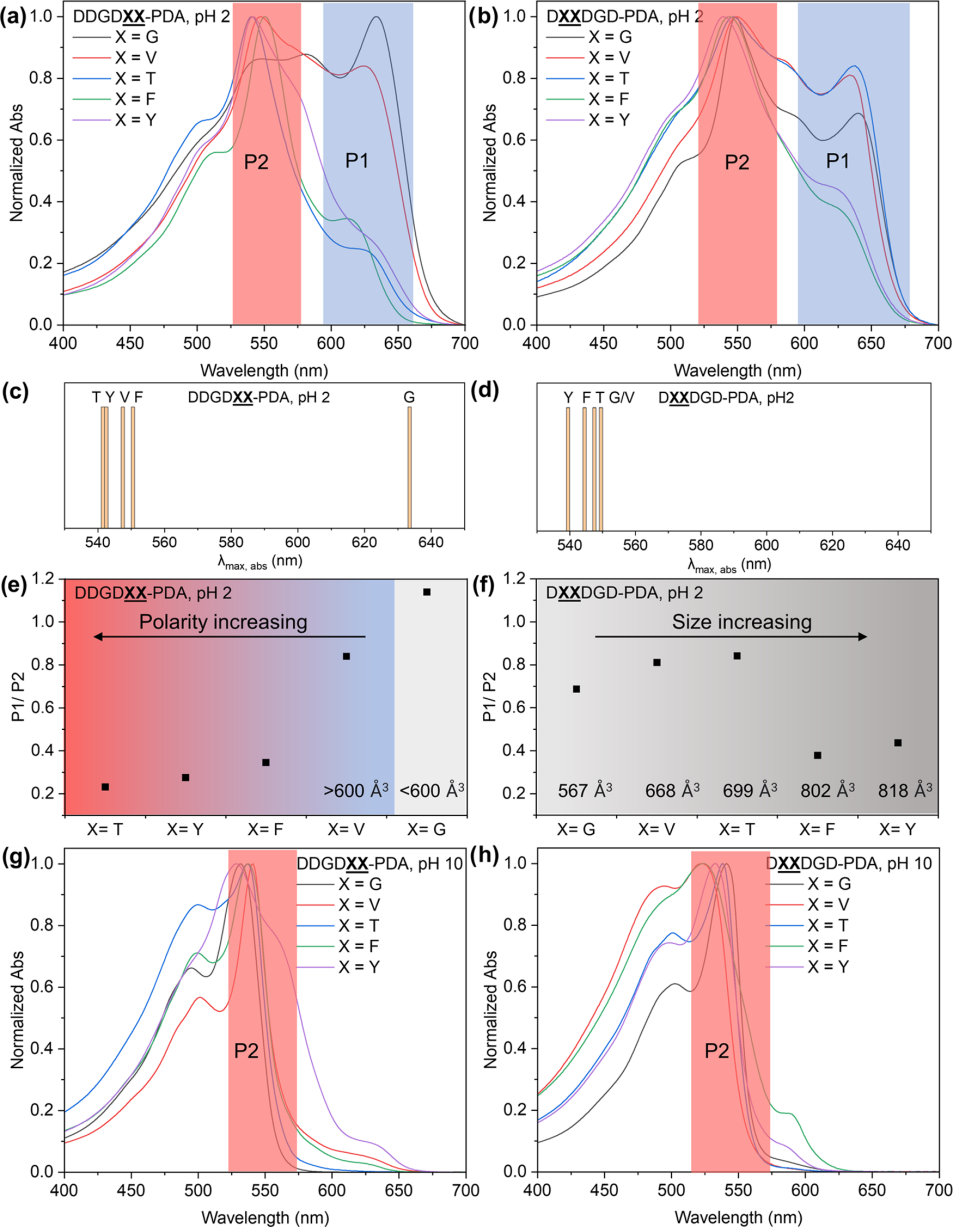
Absorption spectra of 1 mM (a) DDGD**XX**-PDA and (b) D**XX**DGD-PDA polymerized with 40 min 254 nm UV irradiation at pH 2 (X = G, V, T, F, and Y). Schematic diagram showing the hypsochromic shift of λ_max_, for (c) DDGD**XX**-PDA (X = V, T, F, and Y) absorption compared to λ_max_, for DDGD**GG**-PDA absorption at pH 2, and for (d) λ_max_ of D**XX**DGD-PDA (X = V, T, F, and Y) absorption compared to λ_max_ of D**GG**DGD-PDA absorption at pH 2. The P1/P2 intensity ratio for (e) DDGD**XX**-PDA and (f) D**XX**DGD-PDA with 40 min UV irradiation at pH 2 showed size-dependent trends for both isomers where substitution with larger residues favor the formation of red phase. Comparison between sequences of similar size but varying polarity did not show drastic changes in P1/P2 values except for DDGD**VV** and DDGD**TT**. UV–vis spectra of 1 mM (g) DDGD**XX**-PDA and (h) D**XX**DGD-PDA polymerized with 40 min 254 nm irradiation at pH 2 then switched to pH 10.

To explore the molecular contributions of peptide moieties on the photophysical properties of PDA, we performed UV–vis absorption, photoluminescence (PL), and circular dichroism (CD) spectroscopy on dilute aqueous dispersions of peptide-PDA nanostructures. It is well established that the photophysical properties of PDA are directly correlated to the monomer assembly behavior and ultimately, the conformation of the polymer backbone.^3,6,14,44,57^ Here, we utilize the distinct PDA chromatic phases, particularly the blue and red phases, to spectroscopically investigate the torsion experienced by PDAs along the π-conjugated chain as a consequence of the peptide templating groups (Figure 4 and Table 1). Comparing all λ_max_ of DDGD**XX**-PDA (X = V, T, F, and Y) absorption with respect to blue phase DDGD**GG**-PDA, significant hypsochromic shifts of λ_max_ (up to 100 nm) due to dominance in the absorption spectra of red phase peaks are observed. This trend shows that substituting glycines with larger side chains, when they are nearest to the alkyl DA region, leads to pronounced differences in the PDA chain conformation (Figure 4a,c and Table 1). To better assess the impacts of residue polarity and noncovalent interactions such as π interactions between groups with similarly sized side chains, we compared the spectral shifts between **VV**- vs **TT**- and **FF**- vs **YY**-substituted PDAs. Small spectral shifts (6–8 nm) are observed when comparing the λ_max_ for DDGD**VV**-PDA vs DDGD**TT**-PDA, and DDGD**FF**-PDA vs DDGD**YY**-PDA absorption, whose molecular volume of peptide segments are relatively similar while having a considerable difference in polarity (MPI values). To better quantify the torsional impact of the peptide variation on the PDA backbone, we took the intensity ratio of peak 1 (P1, λ_max_ for blue phase) and peak 2 (P2, λ_max_ for red phase). Despite DDGD**GG**-PDA having the highest P1/P2 ratio, we found that the ratio of the less planar red phase increases along with increasing peptide MPI when the molecular volumes of hexapeptides are larger than 600 Å^3^ (Figure 4e). Thus, with respect to DDGD**GG**-PDA, both the increase of steric repulsion and the polarity can impart a twisted conformation when the substitution domain is directly adjacent to the alkyl PDA group. Although the effect of steric bulk is more apparent than the effect of side chain polarity, as suggested by the extent of spectral shifts within this peptide series, the difference between P1/P2 ratios for V- and T-substituted DDGD**XX**-PDA is more notable than the F- vs Y-substituted peptide-PDAs. In sum, for the DDGD**XX**-PDA series under acidic conditions, steric effects predominated over contributions of molecular polarity on PDA conformation.

**Table 1:** Summary of Maximum Absorption Peaks for Peptide-PDAs under Different pH Conditions

DDGD**XX**-PDA	λ_max_ (nm)	D**XX**DGD-PDA	λ_max_ (nm)
X = G, pH 2	633	X = G, pH 2	549
X = V, pH 2	547	X = V, pH 2	549
X = T, pH 2	541	X = T, pH 2	547
X = F, pH 2	550	X = F, pH 2	544
X = Y, pH 2	542	X = Y, pH 2	539
X = G, pH 10	532	X = G, pH 10	542
X = V, pH 10	541	X = V, pH 10	544
X = T, pH 10	537	X = T, pH 10	528
X = F, pH 10	537	X = F, pH 10	523
X = Y, pH 10	528	X = Y, pH 10	534

When the substitution domain is further away from the conjugated backbone, but more accessible to water (D**XX**DGD-PDA; X = G, V, T, F, and Y), less impact of steric effects can be expected on polymer conformation. All peptide-PDAs within this series had λ_max_ of absorption ∼530 to 550 nm (Figure 4b,d and Table 1) and P1/P2 < 1 at pH 2, indicating the dominance of the less planar, red phase regardless of the peptide molecular volume. The spectral shifts of V-, T-, F-, and Y-substituted D**XX**DGD-PDA are also small (<10 nm) compared to D**GG**DGD-PDA. These results suggest that size differences in D**XX**DGD-PDA, from G- to the large F-/Y-, do not endow as much difference in conformation compared to DDGD**XX**-PDA; which is consistent with our expectation. This is also supported by the small 2–10 nm hypsochromic shift recorded for the λ_max_ of T-, F-, and Y-substituted peptides-PDA absorption with respect to D**GG**DDG-PDA absorption. However, Figure 4f indicates that the presence of bulky Y and F residues in the substitution domains show distinctly lower P1/P2 values than the G-, V-, and T-substituted peptides with molecular volumes <750 Å^3^. These spectral results suggest that although the impact of steric repulsion on the absorption maxima is not as drastic as in DDGD**XX**-PDA, the size of residues in the substitution domain for D**XX**DGD-PDA still affects the distribution of blue- or red-phase chains within the ensemble polymers that exist in the acidic aqueous solutions. The P1/P2 values are also comparable when comparing D**TT**DDG-PDA to D**VV**DDG-PDA and D**FF**DDG-PDA to D**YY**DDG-PDA, indicating that the polarity has a subtle impact on PDA conformation when the substitution domain is more accessible to the solvent. For the D**XX**DGD-PDA series, the absorption data demonstrate that the differential interaction of a peripheral XX domain with the solvent does not significantly perturb the DA assembly and the conformation of the resulting peptide.^58−62^ This is also consistent with the smaller MPI difference between D**TT**DGD vs D**VV**DGD than that of DDGD**TT** vs DDGD**VV**, as well as when comparing the MPI difference between the FF- vs YY-substitution of DDGD**XX** and D**XX**DGD peptides. For the sequences containing aromatic side chains, regardless of the position of the substitution, the additional π interactions did not favor the blue planar conformation as all spectra for the 4 peptide-PDAs with aromatic group have low P1/P2. This trend further highlights the contribution of steric effects to the resulting PDA conformation. The collective absorption profiles of PDAs from DDGD**XX**- and D**XX**DGD-PDA show that the torsional impact of peptides on PDA conformation is significantly different when the substitution domain is closer to the solvent or to the DA group even if the two substitution domain positions being compared here are just separated by one amino acid position. Moreover, while hydrophobicity and steric effects both contribute to the final chain conformation of the PDA, our results show that steric repulsion presented to be the more predominant factor in driving the preference toward a planar, blue or twisted, red PDA phase.

For the aqueous dispersions of both DDGD**XX**- and D**XX**DGD-PDA nanostructures, when the pH was switched to 10, an instantaneous color change was observed regardless of the sequence due to the electrostatic repulsion of aspartic acid units. The absorption profiles of all of the samples shifted to spectral signatures that are reminiscent of primarily twisted, red phase PDAs and almost completely diminished P1 (Figure 4g,h and Table 1). More specifically, the spectral shape of the DDGD**XX**-PDA absorbance profiles is similar within the series (Figure 4g), indicating that the electrostatic repulsion between aspartates has a much greater torsional impact on PDA than the size or hydrophobicity of the individual residues. This also suggests that the global nonplanarity of the π-conjugated backbone adapted by DDGD**XX**-PDAs is more similar from one sequence to another than their acidic counterparts. For the D**XX**DGD-PDA series, the absorption peak shapes are also more similar under basic than acidic conditions, with a slight hypsochromic shift of the more hydrophobic VV- and FF-bearing PDAs with respect to the rest of the peptides. The similarity of absorption profiles at pH 10, as well as the range of molecular weights determined via SEC characterization, show that the impact of the relatively small differences in conjugation length on peptide-polymer photophysical properties is minimal and is not influencing the chromatic phase adapted by each sample (Figure 4g,h and Table S1).^63^

When we examined the PL spectra of the polymer samples under basic conditions, DDGD**XX**-PDAs exhibited more apparent spectral differences across the series than the D**XX**DGD-PDAs (Figure S51). The trends in the shifts between λ_em_ at pH 10 do not exactly follow the molecular volume or MPI trends, illustrating the dominance of repulsive electrostatic effects over steric and hydrophobic effects on PDA conformation. We note that the PL spectra of acidic samples are not reported due to their very low signal-to-noise ratio that can be attributed to the existence of a more planar, blue phase with ultrafast relaxation of the excited state.^64,65^ Taken together, the absorption and PL data suggest that when the electrostatic repulsion between D residues is in full effect at high pH, the impacts of size and hydrophobicity on PDA conformation become less significant than when the carboxylates of the peptide segments are fully protonated. Yet, steric and hydrophobic contributions still show more effect when the XX domain is nearer to the DA units than when they are more accessible to the solvent, the peptide C-termini.

**Figure 5:**
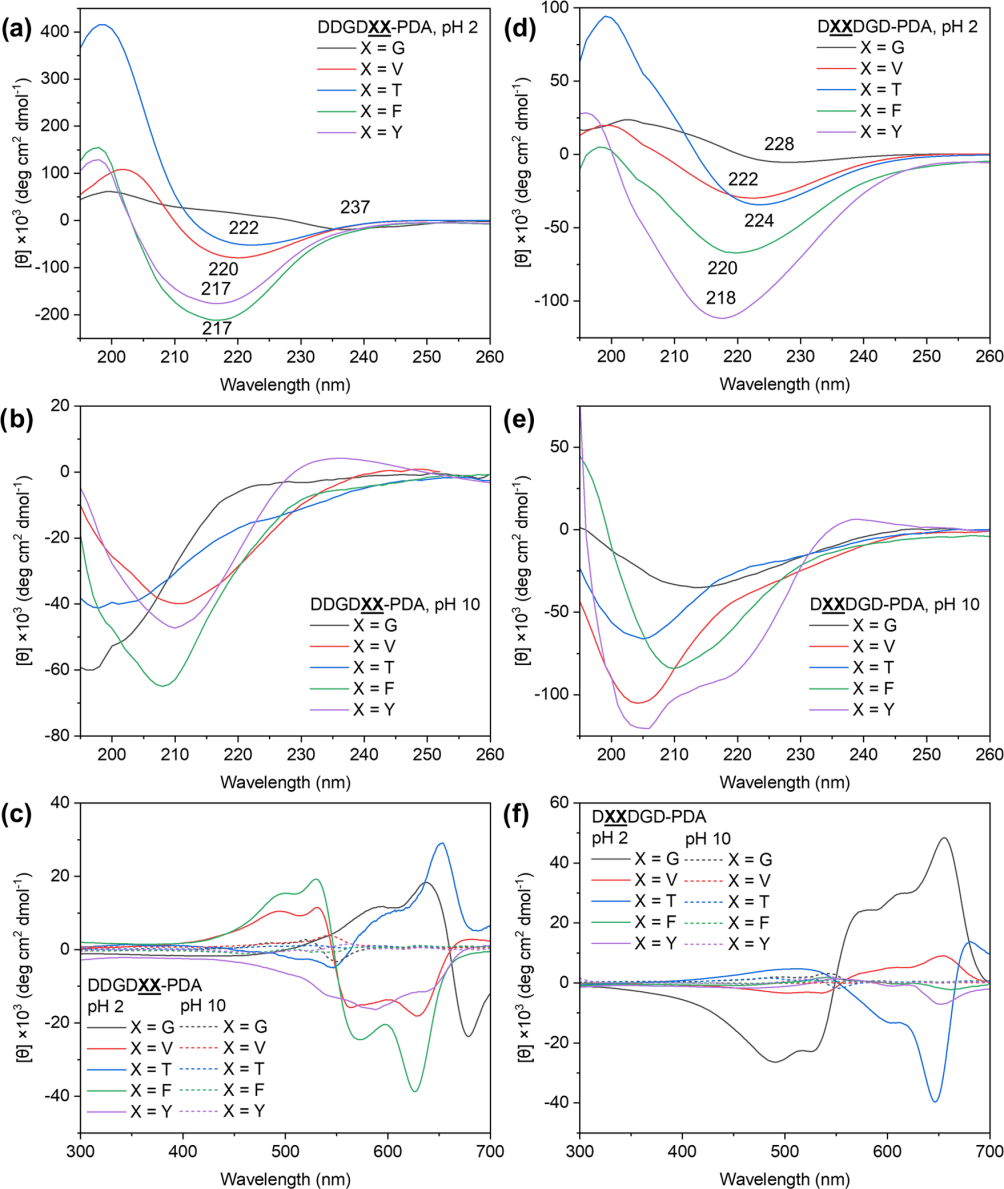
Secondary structure of peptide moieties driven by different supramolecular interactions at different pH conditions. The CD spectra (195–260 nm) of (a) DDGD**XX**-PDA and (d) D**XX**DGD-PDA (X = G, V, T, F, and Y) at pH 2 (peptide-DAs polymerized at 1 mM, and then diluted to 0.1 mM for measurement) show twisted β-sheet-like structures. The CD spectra (195–260 nm) of (b) DDGD**XX**-PDA and (e) D**XX**DGD-PDA at pH 10 (polymerized with 1 mM and then diluted to 0.1 mM for measurement) show features reminiscent of random coil. The CD spectra (300–700 nm) of (c) DDGD**XX**-PDA and (f) D**XX**DGD-PDA at different pH conditions show bisignate signals indicative of PDAs in a chiral environment.

**Figure 6:**
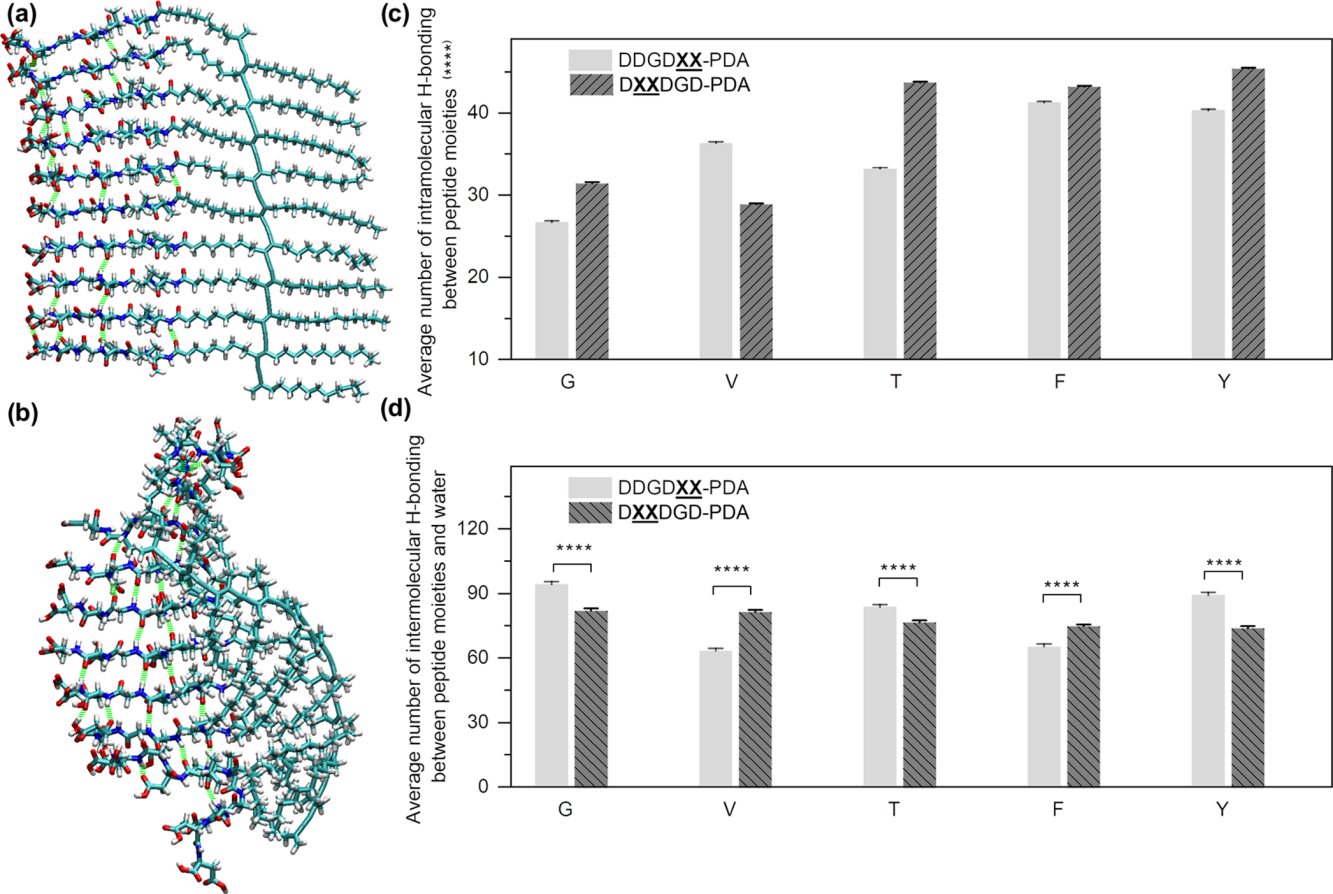
Molecular simulation of an energy-minimized, decamer peptide-PDA model. Molecular structures of a DDGD**VV**-PDA decamer simulated at (a) 0 ns and (b) 10 ns after energy relaxation. Average numbers of hydrogen bonds formed (c) between peptide moieties within the polymer and (d) with water during 10 ns simulation (1000 snapshots); error bars = standard error of the mean. For the statistical analyses, *p*-values were calculated using *t*-test (two-sample unequal variance test, one-tail distribution); ****p < 0.0001. The *p-*values are <0.0001 for all pairs of data for the analysis of hydrogen bonds formed between peptide moieties. The *p*-values for the analysis of hydrogen bonds formed between peptide and water are reported in Table S2. These simulations show that steric effects have a higher impact on intramolecular hydrogen bonding within the peptide region, while the residue polarity-driven changes in peptide–solvent interactions have a higher impact on intermolecular hydrogen bonding.

The absorption and PL spectra recorded for all samples under acidic and basic conditions reinforced the idea that subtle molecular changes in the peptidic templates can be utilized to modulate the resulting electronic structure of the PDA chains correlated to the torsion experienced by the π-conjugated backbone. In the past, spectral shifts between the chromatic transitions have been attributed to changes in both conjugation length and chain order.^7^ However, when comparing the blue and red phases, several experimental and computational demonstrations argued that a decrease in order and a corresponding shortening of the conjugation length is not appropriate for describing the red phase of PDA as an extended 1-D electronic system.^66,67^ We further explore the sequence-based structural modulation for the peptide-PDAs here by performing CD spectroscopy to evaluate any correlation between the observed photophysical properties and the sequence-defined order via the secondary structure adapted by peptidic moieties (Figure 5). Throughout the peptide-DA monomer library, the 0.1 mM peptide-DA monomer assemblies at pH 10 showed predominantly random coil secondary structures but showed β-sheet-like CD bands at pH 2 (Figure S52). The characteristic β-sheet-like band was still observed for all samples after polymerization under pH 2, indicating that polymerization did not completely disrupt the initial assembly and that the sequence-specific peptide interactions promoted DA geometric arrangements favorable toward the topochemical formation of PDA (Figure 5a,d). The exceptions are the polymers with X = G, which showed the absorption spectra most reminiscent of blue phase PDAs but had CD spectra showing a significant decrease in intensity of the characteristic β-sheet-like band from the monomeric assemblies (Figure S52b,d) to that signature observed for the polymeric equivalents (Figure 5a,d). This drastic spectral change can be attributed to G as a small residue that allows more degrees of freedom for the overall assembly/polymer structure. Under acidic conditions, there is also an apparent red shift in the high energy region of the CD spectra of peptide-PDAs going from those with the bulkiest dipeptide substitutions (FF- and YY-) to the G-substituted peptides (Figure 5a,d). The red shift in the CD profiles at this region is indicative of twisted β-sheet-like structures.^25,68^ Correlating with the absorbance data (Figure 4), those that have higher P1/P2 values exhibit the more red-shifted CD minima (DDGD**GG**, ∼235 nm; D**GG**DGD, ∼225 nm). These results suggest that the steric contribution of bulky residues hinders the formation of secondary structures that are appropriate for the formation of a planar conjugated backbone.^69^ Hence, the subtle steric and hydrophobic residue differences within our monomer library demonstrate distinct effects on the molecular- and chain-level order adapted by the peptide-polymer conjugates as shown by the spectroscopic differences. The molecular simulations reported here (Figures 6 and S53–S57) further support the molecular ordering behavior of peptide-polymer conjugates as well as the spectroscopic differences brought by differences in chain conformation. Comparing the acidic and basic peptide-PDA solutions, CD spectra show that increasing the electrostatic repulsion in the peptide region resulted in a structural shift from β-sheet-like to random coils (Figure 5a,b,d,e).

Within the region of PDA absorption (450–700 nm), the CD spectra of all peptide-PDAs at pH 2 exhibited a bisignate profile indicative of the Cotton effect (Figure 5c,f), and therefore suggests that the peptidic region presents a chiral local environment to the conjugated PDA backbone. The chirality brought by peptides to the DA monomers induced circular birefringence in the conjugate polymer structures, with varying degrees and handedness associated with twisted β-sheet regions as modulated by the sequence composition.^52,62^ For the DDGD**XX**-PDA series, the most hydrophobic (lowest MPI) sequences (VV- and FF-substituted) show characteristic negative excitonic chirality with cross-over wavelengths that clearly coincide with the λ_max_ of red phase PDAs absorption. Meanwhile, D**XX**DGD-PDAs with bulky and hydrophobic residues at the substitution domain have CD spectra with low intensity and asymmetric signals, suggesting that the interaction of residues with the solvent is more likely to affect the secondary structure. Upon switching to basic conditions, the structural reorganization of PDAs to a more disordered state is demonstrated by the quenched bisignate signature between 450 and 700 nm, alongside the transition from β-sheet-like structure to random coil observed for the peptide segment. Based on the photophysical properties of the peptide-PDAs studied here, both steric effects and hydrophobic interactions have more synergistic contributions to the PDA order at the secondary structure level than the conformation at the chain level. Steric repulsion in the peptide moiety has a larger impact on the chain conformation and electronic structure, and thus can be used to modulate the chromatic phase of the resulting PDA. On the other hand, residue hydrophobicity and general peptide polarity, via peptide segment-solvent interactions, become more relevant when considering order at the secondary structure level and excitonic chirality of peptide-PDAs. When triggering electrostatic repulsion by switching to basic conditions, regardless of the position or nature of the residue at the substitution domain, all samples adapted a disordered PDA assembly, peptide moieties in random coil, and dominance of the red PDA phase.

### Molecular Dynamics Simulation

To better explain the spectroscopic differences within our sample library, force-field-based molecular dynamics simulations were performed to obtain atomic-level approximations of the designed peptide-PDA structures in aqueous environments (Figures 6a,b and S53–S55). All carboxyl groups were kept in the protonated state to simulate polymer structures under acidic conditions. In particular, we conducted these simulations on a decamer peptide-PDA model to shed light on the synergistic role of hydrogen bonding with the other supramolecular factors such as steric and hydrophobic effects investigated here. To do so, weak and moderate intermolecular hydrogen bonds^70^ formed between peptide units and peptide-water interactions were quantified based on the structural snapshots from molecular dynamics simulations (Figures S56 and S57). Regardless of the substitution position, the average number of hydrogen bonds formed between peptide units is highest when the residues at the XX domain have the bulkiest, aromatic amino acids (FF and YY). This result is consistent with the spectroscopic data, where we concluded that steric effect plays a significant role in templating the conformation of PDA (Figure 6c), along with the consideration of π interactions. We also evaluated the hydrogen bonding between peptide moieties and water as the solvent to further investigate the impact of residue polarity (Figure 6d). When comparing the peptide-PDAs substituted with hydrophobic residues (VV and FF), we observed significantly higher polymer-solvent interactions when the D residues are more distributed throughout the monomer in D**XX**DGD- than for DDGD**XX**-. For the rest of the samples with higher MPI, such as those substituted with GG and OH-bearing residues (TT and YY), there are more H-bonding interactions when these polar residues are closer to the nonpolar core of the assembly. These simulation results can also partially explain why the intensities of CD signals for D**XX**DGD-PDA around 220 nm are relatively weaker compared to those for DDGD**XX**-PDA at pH 2. The observed solvation effects on the model may hinder the hydrophobic interactions necessary to form an ordered secondary structure when XX is further from the PDA backbone, which is also indicated when there are fewer D-units accessible to water.

### Modulation of Peptide-PDA Bulk Film Properties Based on Sequence Templating

**Figure 7:**
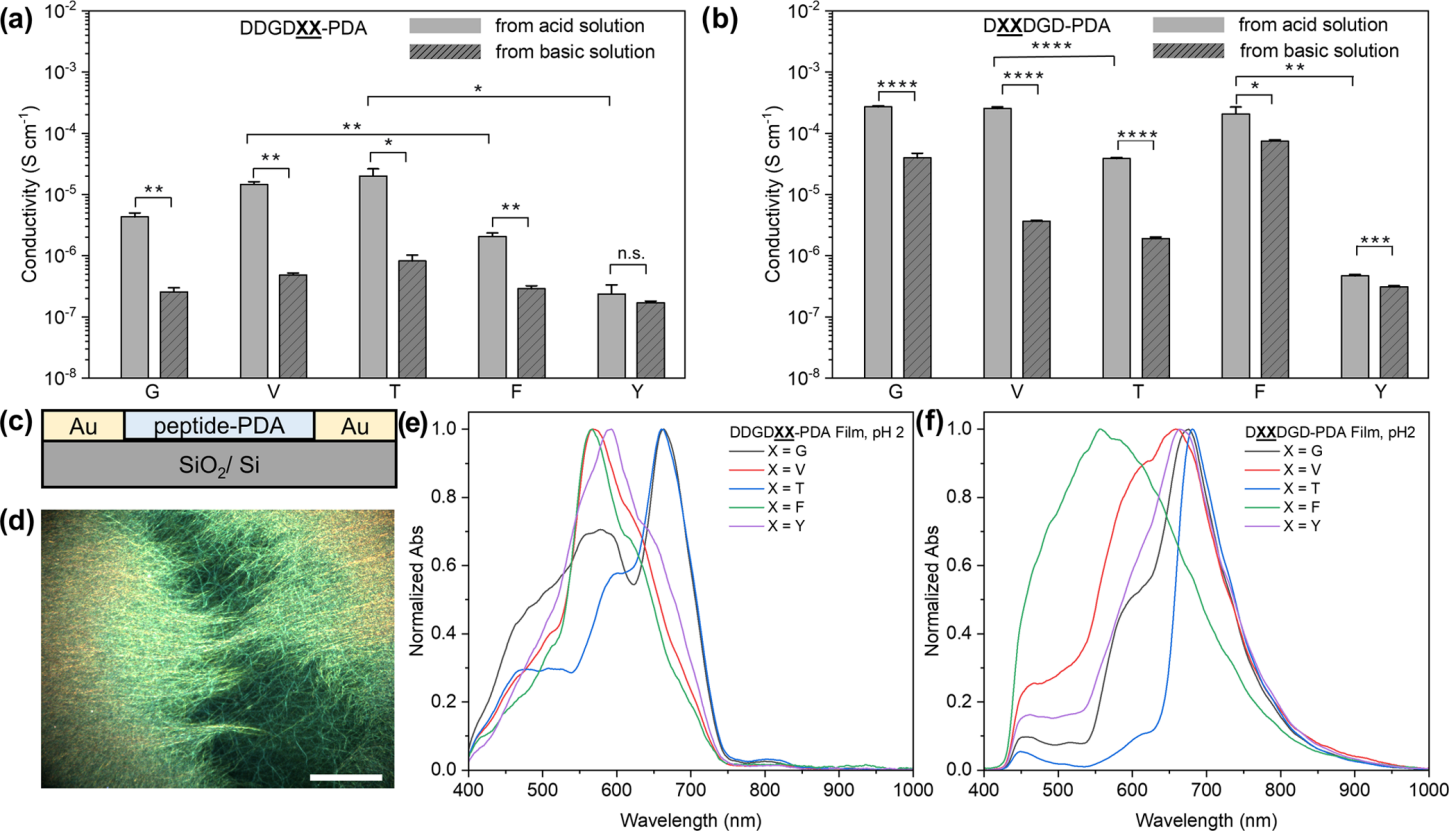
Electrical and optical properties of peptide-PDA films. The conductivity of (a) DDGD**XX**-PDA and (b) D**XX**DGD-PDA dried films from 1 mM peptide-PDA solution with varied pH conditions. Error bars = standard error of the mean; *n* ≥ 4. For the statistical analyses, *p-*values were determined using *t*-test (two-sample unequal variance test, one-tail distribution) See Tables S3 and S4 for detailed information; *p < 0.05; **p < 0.01; ***p < 0.001; ****p < 0.0001; n.s. (not significant; *p* > 0.05). (c) Schematic of conductivity measurement setup for peptide-PDA films. Scale bar = 50 μm. (d) Representative dark-field optical image of DDGD**TT**-PDA films made from pH 10 solution. Absorption spectra, taken from the hyperspectral scans of the area of interest that has the highest matching percentage, of (e) DDGD**XX**-PDA and (f) D**XX** DGD-PDA films made from 1 mM polymer solutions at pH 2.

PDAs have been previously used to dope and conjugate with other organic compounds for improved charge transport.^17−20,71,72^ Beyond understanding the impact of sequence design on the chain conformation and peptide-PDA nanostructures order, we also sought to understand how the bulk properties of the resulting polymeric films can be affected by conformation from sequence-tunable molecular volume and polarity in peptidic segments (Figure 7). Although PDAs have been utilized with other electronic materials due to their unique 1-D electronic nature,^17,18,71−73^ to the best of our knowledge, tuning the electrical properties of biomolecule-templated PDAs based on molecular design is not well studied. Here, we investigated whether the subtle yet systematic changes in the peptide template region, molecular polarity, and residue size will affect bulk peptide-PDA film properties such as electrical conductivity. Two-probe conductivity measurements were performed on polymer films by drop-casting 1 mM peptide-PDA solutions on SiO_2_/Si wafer with pre-patterned gold electrodes (Figure 7c). The film thickness was measured by atomic force microscopy (AFM) (Figures S58–S61). All peptide-PDA films made from pH 2 and pH 10 solutions showed measurable conductivity ranging from 10^–7^ to 10^–3^ S cm^–1^ (Figure 7a,b). The conductivity of peptide-PDA films made from pH 2 condition was significantly higher compared to the same peptide-PDA sample made from the pH 10 condition (except in the case of DDGD**YY**-PDA with the highest polydispersity among the library, Table S1), indicating the relatively more ordered domains in peptide-PDA films with significantly reduced intrachain electrostatic repulsion. These results are consistent with the expectation that global ionization of peptides results in bulk disorder due to electrostatic repulsion under basic conditions, hence decreasing conductivity.

Interestingly, the steric bulk of amino acids still impacted the conductivity of DDGD**XX-**PDA and D**XX**DGD**-**PDA films made under pH 2 conditions, specifically resulting in increased conductivity along with the decrease of the peptidic molecular volume (Figure 7a). This trend is evident when comparing the conductivity of FF-substituted thin film to the VV-substituted sample, as well as the YY- vs TT- substituted DDGD**XX-**PDA and D**XX**DGD**-**PDA films, indicating that the conductivity of peptide-PDAs can be tuned by molecular volume engineering of the polymer side chains/templating groups. These measurements also show that D**XX**DGD-PDAs generally have higher conductivity values than their less hydrophobic (i.e., higher MPI, Figure 2c) isomer series of DDGD**XX**-PDA. In addition, the higher conductivity of D**VV**DGD-PDA film than that of D**TT**DGD-PDA film, and the higher conductivity of D**FF**DGD-PDA film than that of D**YY**DGD-PDA at pH 2 condition support that the polarity/hydrophobicity of residues has more apparent effects in the bulk properties than those directly affected by chain-level conformation (Figure 7b). It also needs to be pointed out that the conductivity values of DDGD**YY**-PDA and D**YY**DGD-PDA with twisted conjugation backbone are the lowest in each group, hindered by the larger peptidic molecular volume and polarity. Overall, these results suggest that residue steric effect and hydrophobicity in peptide segments synergistically affect the order-dependent property of films such as conductivity at the bulk level. In particular, smaller molecular volumes of peptide moieties potentially lead to less steric repulsion between DA units and thus, resulting in PDA chains that create more planar conjugated backbone and thus more ordered domains associated with improved bulk charge transport of the films.^74,75^ On the other hand, interactions with the water solvent as the film dries are impacted by the inherent peptide polarity. By tuning peptidic molecular volume and polarity, we achieved film conductivities ranging from 10^–7^ to 10^–3^ S cm^–1^ and exhibited a general increase in conductivity with smaller peptide molecular volume and lower MPI. Based on the systematic molecular changes employed here, and from what we know about their chain conformation based on their photophysical properties, the results from the bulk electrical property characterization of films provided insights on using side chain engineering to modulate the conductivity of peptide-PDAs for future biosensing or bioelectronic applications.^76−79^

We further investigated the optical properties of the sequence-varied peptide-PDA films and their homogeneity using hyperspectral microscopy with dark-field imaging (Figures 7d and S62–S67). Sample films for this analysis were made by drop-casting 1 mM acidic and basic solutions directly to clean glass slides and then dried overnight under ambient conditions. Throughout the peptide-PDA library, the films exhibited an overall bathochromic shift in spectra compared to the absorption profiles recorded from the aqueous solutions (Figures 7e,f and S67a,b), which could be attributed to further aggregation brought by the drying process. It is also noticeable that the substitution nearest to the PDA region resulted in films with spectra that are more sensitive to the residue variation compared to when the substitution is at the periphery. After spectra collection and mapping multiple regions of interest per film, we performed spectral angle mapping (SAM) and assessed the homogeneity of the chromatic phase adapted across the films. For each film sample, we identified a spectral profile per film that was most represented within a defined region and calculated the percentage of spectral matching per area (Figures S62–S66). The percentage of spectral matching, which is indicative of both spectral homogeneity and surface roughness throughout the films, generally decreases with the increase of residue size for both DDGD**XX**-PDA at pH 2 and pH 10 solutions (Figure S67c,d) but not for D**XX**DGD-PDA (Figure S67e,f). Collectively, considering the results of the conductivity measurements and hyperspectral microscopy, we show that the subtle residue variations in the peptide template region are sufficient to modulate the bulk optical and electrical properties of peptide-PDA films. These measurements also strengthen the argument that the trends in conformation-dependent chromatic phases in solution, blue or red PDAs, are not strictly correlated to bulk film properties. More specifically, the chain conformation-dependent chromatic phases are influenced by molecular design in a distinct manner from the impacts in higher-level structural order, which further supports the need of studies that look into molecular design programmability of material properties at multiple length scales.

### Biocompatibility of Peptide-DA Monomers and Peptide-PDA Films

**Figure 8:**
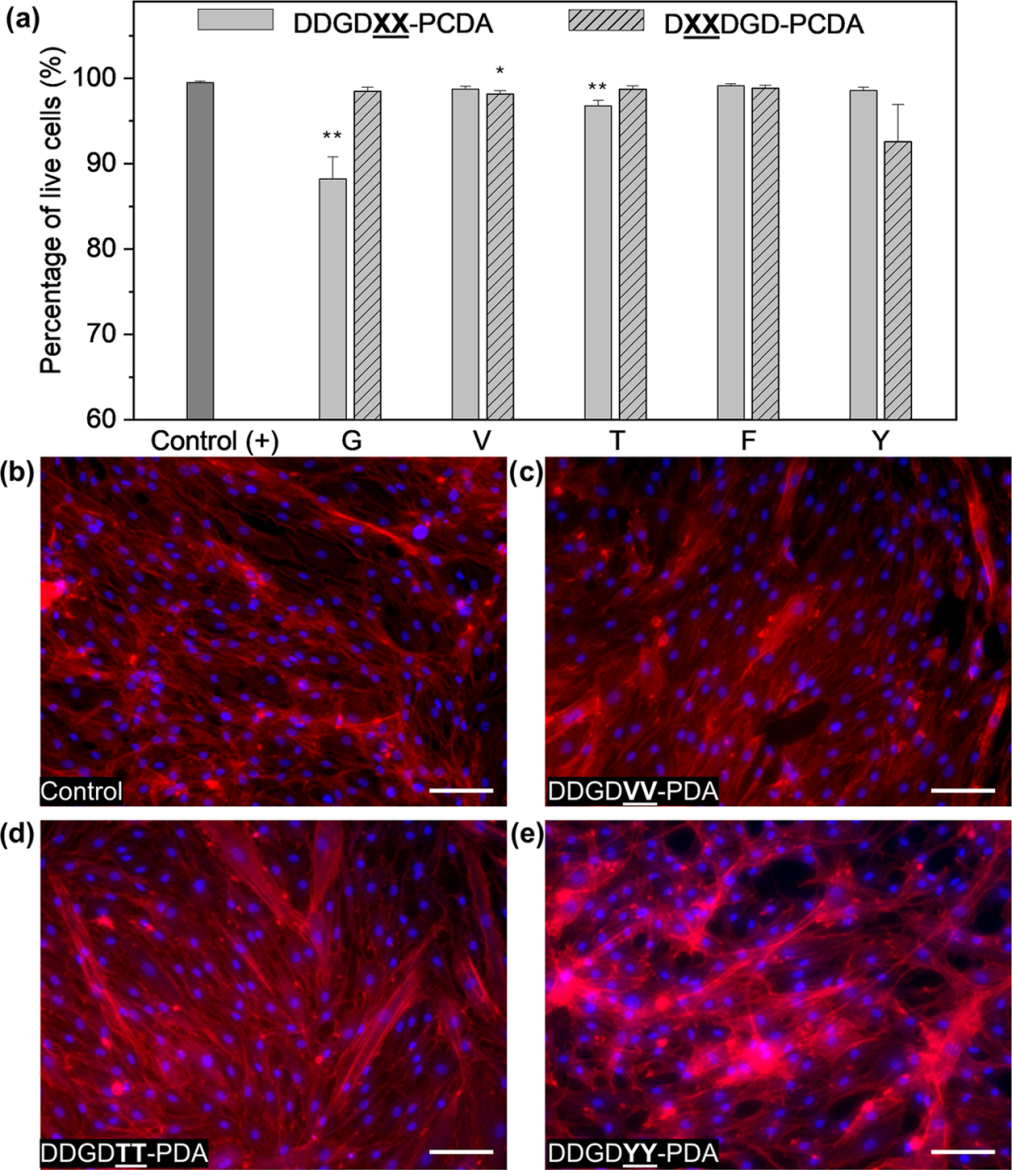
Interfacing peptide-PDA monomers and their polymerized films with HDFs. (a) Cellular viability of HDFs incubated with 1 mM peptide-PDA after 6 h of exposure. Error bars represent the standard error of the mean; *n* = 6. For the statistical analysis, unpaired *t*-test with Welch’s correction was performed; *p < 0.05, **p < 0.01 vs positive control. Representative images of HDFs incubated for 5 days on films made from 5 mM peptide-PDA solutions at pH 2; HDFs on (b) glass coverslips and (c) 5 mM DDGD**VV**-PDA, (d) 5 mM DDGD**TT**-PDA, (e) 5 mM DDGD**YY**-PDA. Cells were stained with DAPI (nucleus) and Phalloidin (F-actin); Scale bar = 100 μm.

Since peptide-PDAs have been demonstrated to have potential use for different biomedical applications,^34,40,42,80^ we evaluated the interaction of our library of peptide-DA monomers and polymerized films with a model human skin cell line, human dermal fibroblasts (HDFs). Through these experiments, we assessed how varying the peptide sequence templates impact the feasibility of these PDAs to be utilized for applications such as cutaneous tissue engineering or sensing at the interface of dermal tissues.^46,81,82^ To assess how sequence variation differentially impacts cell viability, acute exposure of peptide-DAs was performed by incubating HDFs with 1 mM monomer dispersions in an electrolytic, buffered medium for 6 h (Figures S68 and S69). Except for the HDFs exposed to DDGD**GG**-DA, all other peptide-DAs show ≥90% viability, demonstrating the biocompatibility of peptide-DAs under physiologically relevant conditions (Figure 8a). The size of nanomaterials is often one of the most crucial physicochemical factors in determining the cytocompatibility of nanostructures. It is worth noting that the sequence with the lowest molecular volume (DDGD**GG**-) had the lowest cell viability within our peptide-PDA library. In addition, at higher concentrations, the polymerized assembly can form a film serving as a substrate for HDF growth *in vitro*. Here, we prepared 5 mM peptide-DA solutions that were assembled with the aid of the slow hydrolysis of glucono-δ-lactone (10 mg/mL) to make uniform films. All polymers from our library successfully formed polymerized films via UV irradiation, but only those resulting from DDGD**VV**-, DDGD**TT**-, and DDGD**YY**-PDA resulted in films that are stable in cell culture media for at least 5 days. These are the sequences with substitution domains nearest to the PDA region, bearing mid-sized residues (V and T) and a bulky, but −OH-containing Y residue. This observation shows the importance of peptidic design, particularly the balance between the attractive and repulsive interactions within the peptide moieties, when considering building scaffolds that rely on hierarchical 1-D assembly for polymer chain formation and chain entanglements to form stable films (or even hydrogels for future applications). After culturing HDFs on the VV-, TT-, and YY-substituted peptide-PDA films for 5 days, HDF monolayers formed on peptide-PDA films similar to those seeded on control glass coverslips (Figures 8b–d and S70). However, the cellular morphologies and apparent density of actin stress fibers qualitatively observed for DDGD**YY**-PDA, which has the largest monomer volume, and poorest surface homogeneity (Figures 8e, S67, and S70) within the DDGD**XX** series, were different from the control and the other peptide-PDA films. While a more deterministic set of design rules to predict this biointerfacing behavior would require a larger library of peptides to screen, our results herein set the foundation for programming cellular viability and cellular phenotype via peptide design for bioscaffolds built from peptide-PDAs materials, which will be investigated in detail in the future.

## Conclusions

In this study, we demonstrate the ability of systematic peptide sequence templating of amphiphilic DAs to rationally tune the properties of the resulting PDAs as a chromatically adaptive, π-conjugated polymer that can be utilized under physiologically relevant conditions. We built a library of hexameric peptide-DA monomers with dipeptide substitution domains that help delineate the contributions of sterics and residue polarity to the multiscale structure and behavior of peptide-PDA materials. Our results show that steric repulsion, especially near the PDA region, has more torsional impact on the conformation of the PDA chains than the inherent hydrophobicity of peptides. These findings demonstrated significant differences in properties based on the positional isomers of peptide-polymer PDAs studied herein. Photophysical measurements show that substitutions with bulky residues generally lead to the twisted, red phase PDA. We also show that changes in the secondary structure of the peptide moieties translate the changes in the degree of excitonic chirality experienced by the PDA chains. A transition from β-sheet-like structures to random coils upon changing from acidic to basic conditions guided the PDAs to adapt a more twisted chain along with disordered structures due to electrostatic repulsion. Molecular simulations showed that the impact of peptide variation is more pronounced on intramolecular hydrogen bonding within the peptide region rather than on the polymer–solvent hydrogen bonding. The subtle systematic substitutions on the peptide segments resulted in measurable changes in bulk properties such as electrical conductivity, which is known to be dependent on the order of polymer ensembles in film and was established to have distinct trends from how the chain conformation-dependent chromatic phase and electronic structure was affected by sequence design in solution state. Moreover, the systematic sequence variation on the peptide template was sufficient to observe changes in the interaction of peptide-DA monomer suspensions and peptide-PDA films with human dermal fibroblasts. In conclusion, the ability of peptide sequences to template the aqueous assembly of diacetylene monomers toward a specific chromatic phase can be leveraged to modulate the secondary structure, as well as bulk order of these peptide-polymer biomaterials—demonstrating an approach to finely and distinctly tune properties at multiple length scales. These experimental findings on controllable material properties across length scales offer insights into the molecular programmability of the behavior of these peptide-polymer conjugates as functional biomaterials in physiologically relevant aqueous environments.

## Supporting Information Available

Peptide-DA synthesis and peptide-PDA polymerization protocol in aqueous conditions; molecular characterization; nanostructure characterization; quantitative analysis of molecular volume and MPI; additional absorbance, PL, and CD spectra; molecular simulation methods and additional data; supplementary results from hyperspectral scans, conductivity measurements of peptide-PDA films, and AFM measurement of film thickness; and representative images from the cell viability assay (PDF)
