# Supplementary material for: Biomimetic Sequence-Templating Approach toward a Multiscale Modulation of Chromogenic Polymer Properties
Source: Macromolecules. 2023 Jun 15;56(12):4526–40. doi: 10.1021/acs.macromol.3c00403 (PMC10311629; doi:10.1021/acs.macromol.3c00403)
Supplement: Supplementary file 1 — ma3c00403_si_001.pdf [file ma3c00403_si_001.pdf]

Supporting Information

Biomimetic Sequence-Templating Approach toward a Multiscale Modulation of Chromogenic Polymer Properties

Yuyao Kuang[1], Ze-Fan Yao[1,2], Sujeung Lim[1], Catherine Ngo[1], Megan Alma Rocha[3], Dmitry A. Fishman[3], Herdeline Ann M. Ardoña[1,2,3,4]*

[1]Department of Chemical and Biomolecular Engineering, Samueli School of Engineering, University of California, Irvine, CA 92697

[2]Department of Biomedical Engineering, Samueli School of Engineering, University of California, Irvine, CA 92697

[3]Department of Chemistry, School of Physical Sciences, University of California, Irvine, CA 92697

[4]Sue & Bill Gross Stem Cell Research Center, University of California, Irvine, CA 92697

*Corresponding author: Herdeline Ann M. Ardoña

E-mail: hardona@uci.edu

Table of Contents

Materials and Methods	S3
Monomer NMR characterization	S10
Monomer ESI-MS characterization	S20
Analytical HPLC traces	S30
Polymer NMR and SEC characterization	S33
Calculation of molecular volume and molecular polarity index	S46
Spectral characterization and TEM images	S48
Molecular simulation	S55
AFM characterization of peptide-PDA films	S61
Optical properties of peptide-PDA films	S67
Fluorescence images of cells on PDA films	S73
References	S76

Materials

All amino acids and Wang resin loaded with Asp (OtBu) were obtained from Advanced ChemTech. The following reagents were obtained from Oakwood Chemicals: *N*-methyl-2-pyrrolidone (NMP), benzotriazol-1-yl-oxytripyrrolidinophosphonium hexafluorophosphate (PyBOP), trifluoroacetic acid (TFA), triisopropylsilane, and diisopropylethylamine (DIPEA). DIPEA was degassed by sparging with nitrogen for 30 ~ 90 min over 4 Å molecular sieves. 10,12-pentacosadiynoic acid (PCDA) was obtained from Sigma-Aldrich. Dichloromethane (DCM), methanol, acetonitrile (ACN), acetic acid, ammonium hydroxide, potassium hydroxide (KOH), ethyl ether (Et_2O), hydrochloric acid (HCl), 4′,6-diamidino-2-phenylindole (DAPI), Alexa Fluor 546 Phalloidin, bovine serum albumin (BSA), Triton-X, LIVE/DEAD assay kit, and 1× phosphate buffered saline (PBS) were obtained from Fisher Scientific. 16% paraformaldehyde (PFA) was bought from Electron Microscopy Sciences. Fibroblast growth medium (Ready-to-use) (PromoCell) contains 1 ng mL^{-1} basic fibroblast growth factor and 5 µg mL^{-1} insulin in 500 mL basal medium. All commercially available chemicals were used without further purification unless otherwise noted.

Methods

Synthesis of peptide-diacetylene monomers and of peptide-PDA polymers. All peptides were synthesized using a Liberty Blue Automated Microwave Peptide Synthesizer based on standard Fmoc solid-phase technique with Wang resin pre-loaded with the terminal amino acid. To conjugate resin-bound peptides to PCDA, the method proposed by Diegelmann et al. for peptide-diacetylene (peptide-DA) monomers was followed.[1] The resin was then washed with NMP, methanol, and DCM. After completely drying under vacuum, the resin was cleaved by mixing with 15 mL of a mixture of TFA, water, and triisopropylsilane with the ratio of 95:2.5:2.5 for 2-3 hours.

The resin was washed with 10 mL of DCM and then filtered. The collected solution was reduced to about 2 mL with a Buchi R-100 rotovap. The remaining solution was precipitated by adding 40 mL cold Et_2O. After 10 min of 4000 rpm centrifugation, the supernatant was decanted. Then the peptide pellet was dried with air and resuspended in water with a minimal amount of ammonium hydroxide, and then lyophilized.

Purification of peptide-DA monomers. The purified product was obtained by reverse phase-HPLC using Agilent 1260 Infinity II with a Zorbax Eclipse XDB-C8 column (21.2 × 150 mm, particle size 7 μm). The mobile phase used was 0.1 wt% ammonium formate in water (pH = 10) (solvent A) and acetonitrile (solvent B). Samples were purified using a gradient of 90%A/10%B to 20%A/80%B and signals were monitored at 220 nm. The purity of the samples was further confirmed through the analytical HPLC traces with a Zorbax Eclipse XDB-C8 column (4.6 × 150 mm, particle size 5 μm).

ESI-MS characterization for purified peptide-DA monomers. The purified peptide-DAs were dissolved in Milli-Q filtered water to 0.1 mg/mL. Minimum amount of 1M KOH was added for the solubility. Mass spectra were collected with Waters Acquity UPLC H-Class System equipped with Acquity QDa mass detector in negative mode. In the collected mass spectra, all unlabeled minor peaks are lower than 0.5% compared with the intensities of the highest peak in each spectra, indicating the high purity of DA monomers. These minor peaks are highly likely to be resulting from the background noise signals of the instruments.

^{1}H-NMR spectra of peptide-DA monomers and peptide-PDA polymers. ^{1}H-NMR spectra of peptide-DA monomers and peptide-PDA polymers were collected using a Bruker Avance 500 MHz system. For monomeric samples, 10 mg/mL peptide-DAs were dissolved in D_2O with minimum amount of NaOD for solubility. All monomer NMR spectra were collected with 96 scans. For polymeric samples, 1 mM peptide-DA monomers were polymerized using UV irradiation (254 nm, 6 W) for 40 min at pH 2. After dialysis and lyophilization, 10 mg/mL peptide-PDAs were resuspended in D_2O with minimum amount of NaOD for solubility. Then, ^{1}H-NMR spectra of peptide-PDA polymer samples were collected with 144 scans to increase signal-to-noise ratio. The NMR peaks of peptide-PDA are broadened and less defined as compared to the monomeric equivalent, as expected.

Peptide-polymer molecular weight characterization. A multi-detector gel permeation chromatography/size exclusion chromatography (GPC/SEC) system (Viscotek 305 TDA system, Malvern Analytical, Malvern, United Kingdom) was used to characterize the molecular weight of peptide-polymer samples. A PN3211 UV/Vis detector was attached in tandem to the inline system and measured the absorption at 450 nm. A PL aquagel-OH MIXED-H column with a flow rate of 0.5 mL/min was used with 0.1% ammonium formate as the eluent. Poly(ethylene oxide) standards were used for SEC calibration.

Transmission electron microscopy (TEM). Samples were prepared by dropping 10 μL solution onto 200 mesh copper grids with Formvar film and 4nm carbon coating followed by 30 s adsorption. Then grids were washed with Millipore water and stained with a 1% uranyl acetate solution for 30 s. The grids were dried with filter paper. TEM was performed with JEOL JEM-

2100F equipped with CCD camera at accelerating voltage of 200 kV. TEM images were analyzed and visualized using DigitalMicrograph software and ImageJ.

UV-Vis absorption spectroscopy. The UV-Vis absorption spectra of peptide-PDAs were recorded using a Cary 100 UV-Vis spectrophotometer. Spectroscopic samples were made by diluting a peptide-DA solution with Millipore water to 1 mM. The sample was self-assembled by switching pH to 2 with 1 M HCl. Then UV-Vis spectra were recorded for solutions placed in a cuvette with 1 mm pathlength. Samples were then polymerized with short wave UV-irradiation (254 nm, 6 W) for 40 min. The distance between cuvette and UV lamp was around 10 cm. Then UV-vis spectra of polymer solution in an acidic environment were tested. After this, the minimal amount of 1 M KOH was added to switch pH to 10. Then UV-vis spectra of polymer solution in basic environment was tested.

Photoluminescence spectroscopy. The photoluminescence spectra of peptide-PDAs were recorded with Cary Eclipse fluorescence spectrometer for 1 mM peptide-PDA solutions under a basic aqueous environment. Samples were excited with their respective wavelength of maximum absorption. Both excitation slit and emission slit widths were set at 10 nm. The value of PMT detector voltage was set at 1000.

Circular dichroism (CD). The CD spectra were obtained by Jasco J-810 spectropolarimeter. Spectroscopic samples were prepared with Milli-Q filtered water with 1M KOH and 1M HCl to switch pH, similar to the sample preparation for UV-Vis and PL measurements.

Darkfield hyperspectral microscopy collection and spectral angle mapping (SAM). A hyperspectral microscope with CytoViva Enhanced Darkfield Illuminator was used. Samples in a dry film state were prepared by adding 80 μL 1 mM sample solution into a 0.5 cm × 0.5 cm area on the glass slides. Darkfield images were taken with 50× objective and 50 ms exposure time for the region of interest, and then scanned to get hyperspectral images (HSI). SAM was performed using the Envi software with 0.1 radians.

Molecular simulation. Initial geometries of the peptides were generated using Avogadro (https://avogadro.cc). The geometries of the peptide were optimized using ORCA with density functional theory (DFT) at the B3LYP-D3/def2-SVP level.[2] After the peptide was connected to PCDA, geometries of peptide-DA were optimized using GFN-xTB2 method due to more than 100 atoms and conformation flexibility of long alkyl chains.[3] Final single-point calculations with optimized structures were performed using ORCA with density functional theory (DFT) at the B3LYP-D3/def2-SVP level. Molecular polarity index and molecular volume were calculated using Multiwfn program with the DFT-produce wavefunctions.[4,5] To build the polymer system, the DA part was connected to the peptide structures and then a single polymer chain (decamer) was constructed with 10 repeating units with methylene as the end group. The decamer polymer chain was placed in the center of a periodic boundary conditions (PBC) box while water molecules were used to fill the box. The built-in OPLS-2005 force field was adopted to describe the interactions between atoms.[6] During the simulations, Nose-Hoover chain thermostat (relaxation time: 1 ps), Martyna-Tobias-Klein barostat (relaxation time: 2 ps), and timestep of 2 fs were used. The short-range electrostatics and van der Waals interactions were truncated at 0.9 nm. The long-range electrostatic interactions were summed using particle mesh Ewald (PME) method. The simulated

systems were undergone a standard relaxation procedure. Then, 10-ns production simulations were performed under the NPT ensemble at 300 K and 1 bar with a sampling interval of 5 ps. The last 5-ns snapshots were collected for the structural analysis. All-atom molecular dynamics (MD) simulations were performed using Desmond and Maestro academic version.[7] The visualization and analysis of simulated structures were performed using Mercury and VMD softwares.[8,9] Hydrogen bonds were counted using the "moderate-strength" definition when the donor and acceptor distance is shorter than 3.2 Å and the donor and acceptor angle is below 50°.[10] The above computational work utilized the infrastructure for high-performance and high-throughput computing, research data storage and analysis, and scientific software tool integration built, operated, and updated by the Research Cyberinfrastructure Center (RCIC) at the University of California, Irvine (UCI). The RCIC provides cluster-based systems, application software, and scalable storage to directly support the UCI research community. https://rcic.uci.edu

Conductivity measurements. Peptide-PDA solutions were prepared at a concentration of 1 mM at pH 2 under UV irradiation for 40 min. Then, the polymer solutions at pH 2 or pH 10 were dropcasted to a SiO_2/Si substrate with pre-patterned gold electrodes and dried under ambient conditions. I~V curves were measured to calculate the conductivities under ambient conditions using Keithley 4200 SCS semiconductor parameter analyzer. Film thickness was determined to calculate the conductivities using atomic force microscopy (AFM). AFM was performed using an Anton Paar Tosca 400 AFM using the tapping mode under ambient conditions. A silicon cantilever with a resonant frequency around 280 kHz was used during the scans. AFM images were analyzed and visualized using Gwyddion software.

Cell viability test for peptide-DA monomers interfaced with HDFs. Human dermal fibroblasts (HDFs, passage number ≤ 10) were seeded on 12-well plates. At ~60% confluency, HDFs were then incubated with 0.1 mM peptide-DA suspended in 250 μL medium. After 6 h incubation, the peptide suspension was aspirated, then the wells were washed once with Tyrode's solution. This step was followed by the addition of 250 μL LIVE/DEAD Assay Reagent (5 μL Calcein AM and 30 μL Ethidium homodimer-1 dissolved in 10 mL Tyrode's solution), which was incubated with the treated HDF wells for 30 min, as well as the controls (positive control: no treatment; negative control: exposed to methanol for 30 mins). The stained HDF wells were imaged using a BZ-X800 Keyence fluorescence microscope.

Utilizing peptide-PDA films for cell seeding. Solutions of 5 mM peptide-DA with 10 mg/mL glucono-δ-lactone (GdL) were dropcasted on glass cover slips, and then polymerized with 254 nm UV irradiation for 30 min. HDFs (passage number ⩽ 10) were seeded on peptide-PDA-coated glass cover slips. After seeding HDFs on films, the cell culture medium was changed every 2 days. After 5 days of incubation, HDF were fixed with 4% PFA and stored in 4°C fridge. After aspirating out PBS from samples, 0.05% Triton-X in 1X PBS was added to each sample for 10 minutes of permeabilization. Then, samples were washed with PBS for three times, followed by incubation with 5% BSA in 1X PBS for 30 minutes at room temperature. After washing three times with 0.5% BSA in 1X PBS, DAPI (blue; 1:500 dilution) and Alexa Fluor 546 Phalloidin (red; 1:200 dilution) was prepared in 1% BSA in 1X PBS, and then added to the sample for 1 h incubation. After washing three times with 0.5% BSA and 1X PBS, films were mounted onto glass slides and imaged using a BZ-X800 Keyence fluorescence microscope.

NMR characterization of peptide-DA monomers

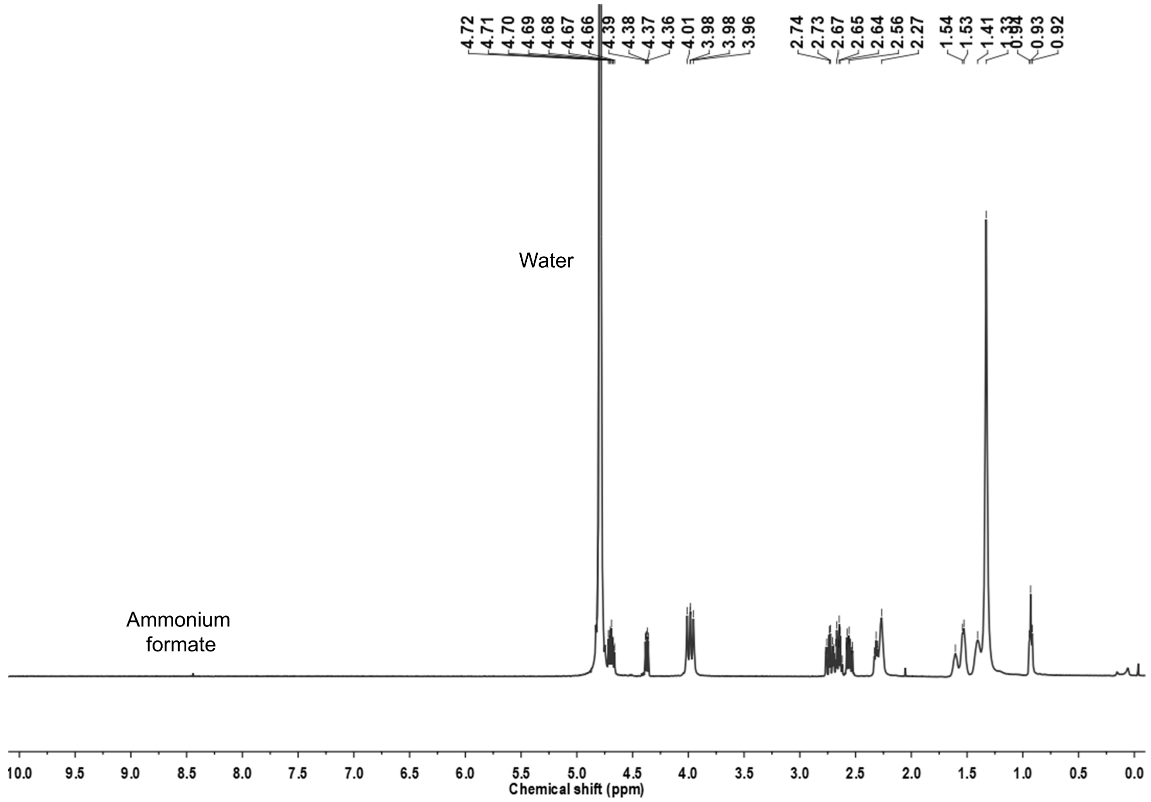

Figure S1. DDGD**GG**-DA characterization by [1] H-NMR (500 MHz, D_2O) δ, ppm: 4.36-4.39, 4.66-4.72 (m, Asp-αH, 3H), 3.96-4.01 (t, *J* = 13.93 Hz, Gly-αH, 6H), 2.53-2.77 (m, Asp-βH, 6H), 2.27-2.33 (m, PCDA-H, 6H), 1.33-1.60 (m, PCDA-H, 32H), 0.92-0.94 (t, *J* = 6.30 Hz, PCDA-H, 3H).

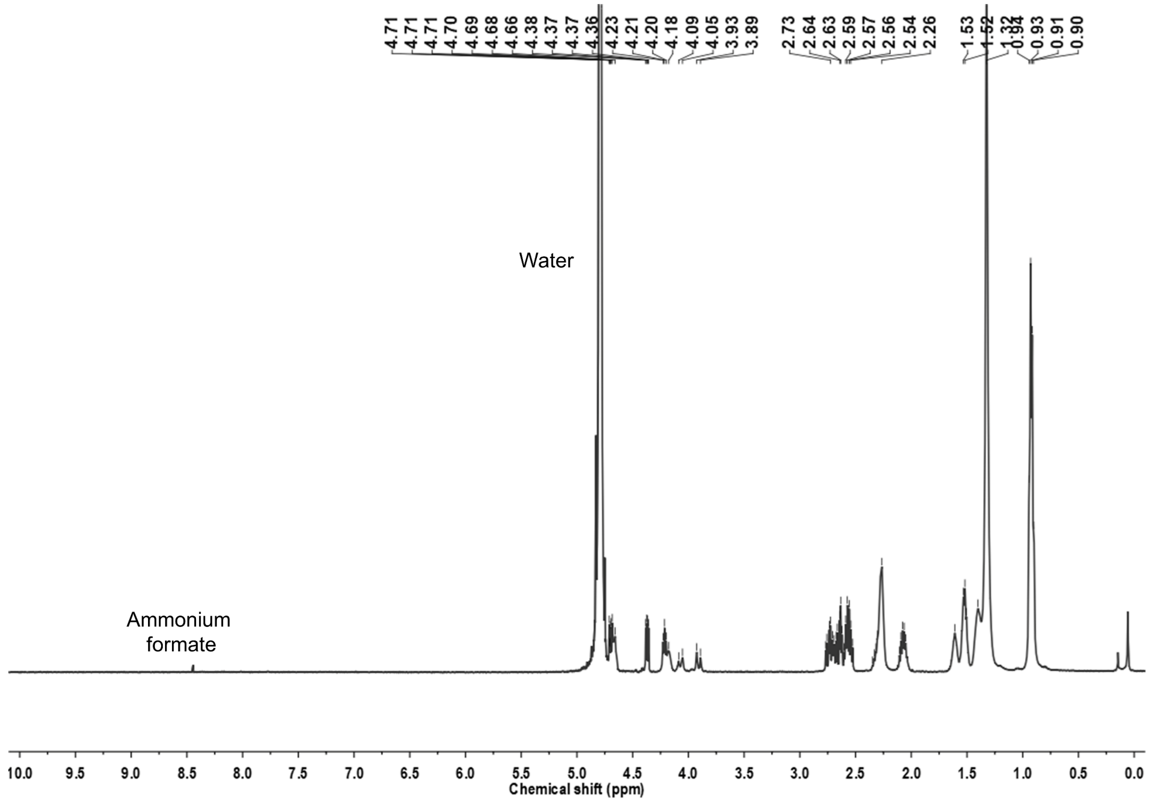

Figure S2. DDGD<u>**VV**</u>-DA characterization by ^{1}H-NMR (500 MHz, D_2O) δ, ppm: 4.36-4.38, 4.66-4.71, (m, Asp-αH, 3H), 4.18-4.23 (m, Val-αH, 2H), 4.05-4.09 (d, J = 16.85 Hz, Gly-αH, 1H), 3.89-3.93 (d, J = 16.65 Hz, Gly-αH, 1H), 2.53-2.77 (m, Asp-βH, 6H), 2.05-2.11, 2.26-2.35(m, PCDA-H, 6H), 1.32-1.61 (m, PCDA-H, 32H), 0.90-0.94 (m, PCDA-H, 3H; Val-γH, 12H).

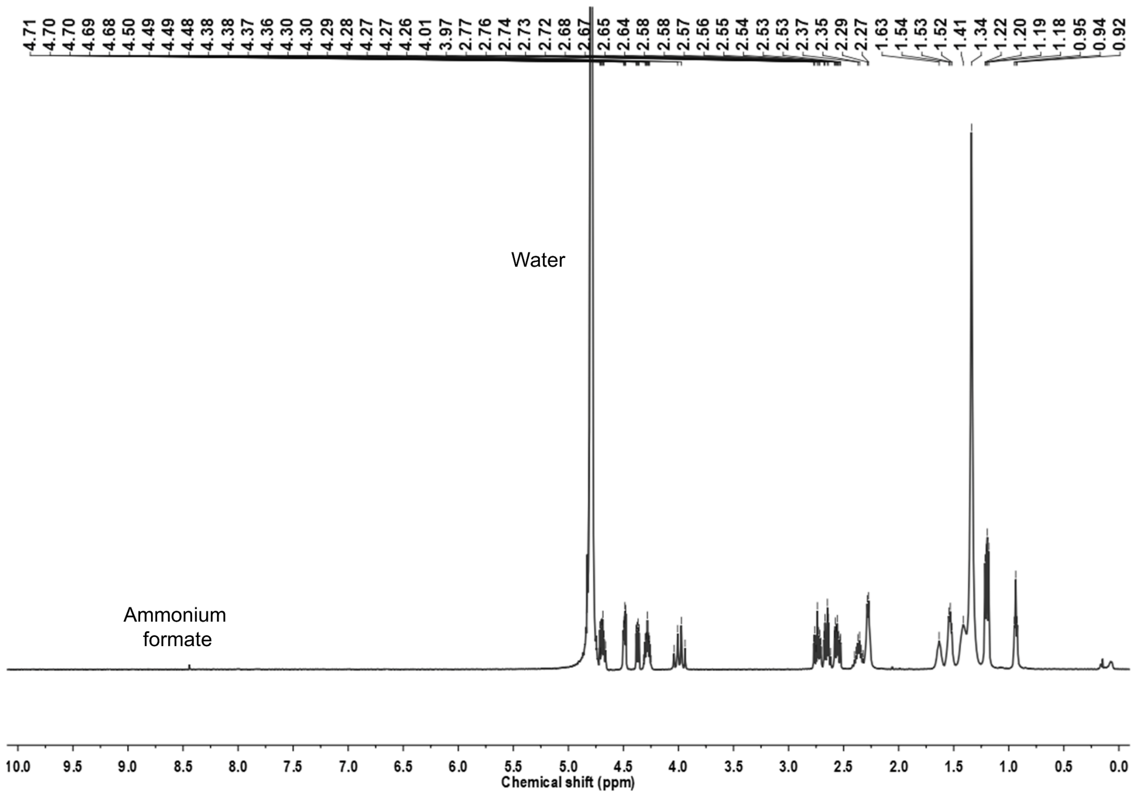

Figure S3. DDGD**TT**-DA characterization by [1] H-NMR (500 MHz, D_2O) δ, ppm: 4.36-4.38, 4.48-4.49, 4.66-4.71, (m, Asp-αH, 3H), 4.25-4.32 (m, Thr-αH, 2H), 3.94-4.04 (q, *J* = 16.75 Hz, Gly-αH, 2H), 2.53-2.58, 2.62-2.68, 2.71-2.77 (m, Asp-βH, 6H), 2.27-2.29, 2.34-2.40 (m, PCDA-H, 6H), 1.34-1.63 (m, PCDA-H, 32H), 1.18-1.22 (m, Thr-γH, 6H), 0.92-0.95 (t, *J* = 6.48 Hz, PCDA-H, 3H).

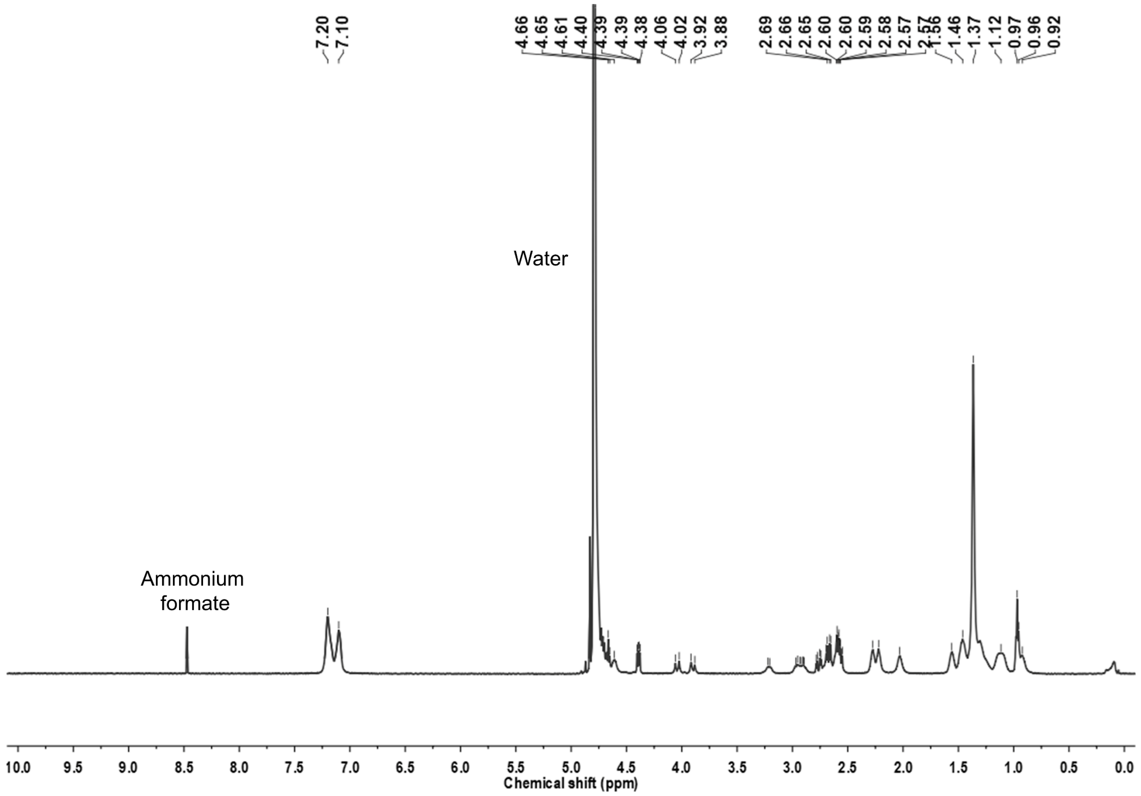

Figure S4. DDGD**FF**-DA characterization by ^{1}H-NMR (500 MHz, D_2O) δ, ppm: 7.10-7.20 (m, Phe-aromatic H, 10H), 4.38-4.40, 4.61-4.66 (m, Asp-αH, 3H, Phe-αH, 2H), 3.88-3.92, 4.02-4.06 (m, Gly-αH, 2H), 2.74-2.78, 2.89-2.97, 3.21-3.22 (m, Phe-βH, 4H), 2.55-2.69 (Asp-βH, 6H), 2.22-2.27 (d, J = 26.2 Hz, PCDA-H, 4H), 2.03 (s, PCDA-H, 2H), 1.12-1.56 (m, PCDA-H, 32H), 0.92-0.97 (m, PCDA-H, 3H).

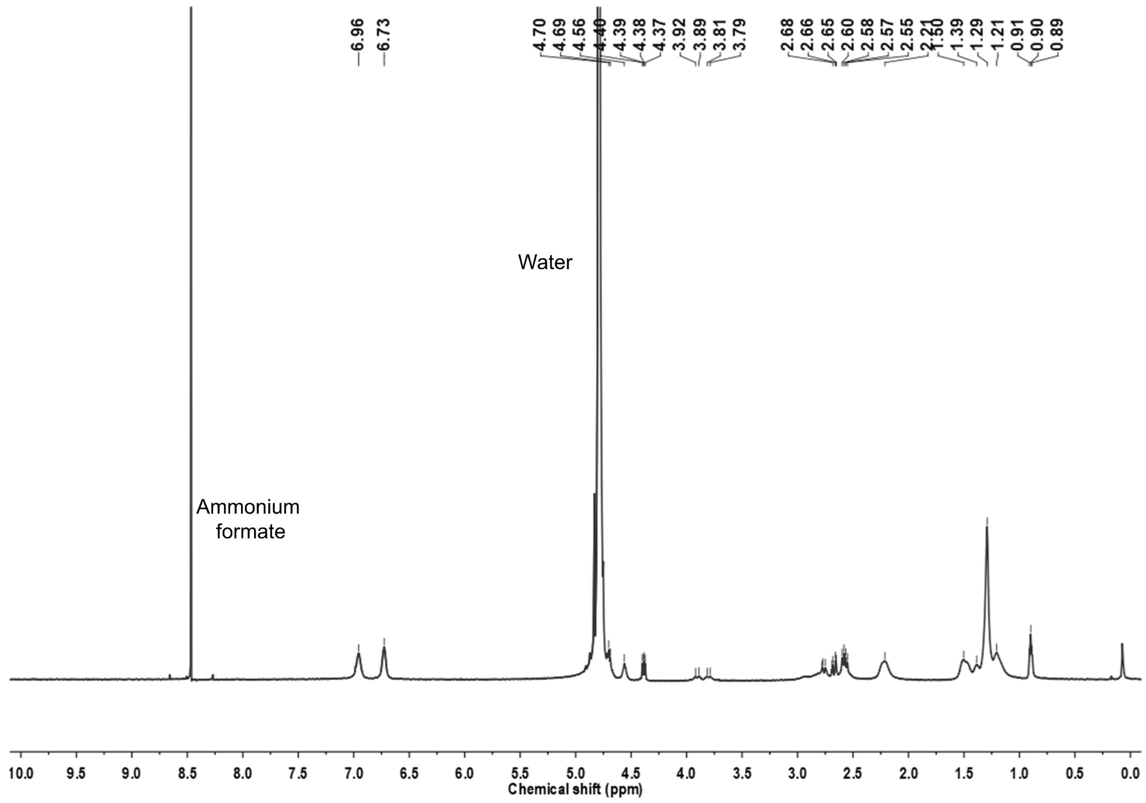

Figure S5. DDGD**YY**-DA characterization by ^{1}H-NMR (500 MHz, D_2O) δ, ppm: 6.96 (s, Tyr-2,6H, 4H), 6.73 (s, Tyr-3,5H, 4H), 4.56, 4.69-4.70(m, Asp-αH, 3H, Tyr-αH, 2H), 4.37-4.40 (d, J = 12.4 Hz, Gly-αH, 1H), 3.79-3.92 (d, J = 14.5 Hz, Gly-αH, 1H), 2.55-2.78 (m, Asp-βH, 6H, Tyr-βH, 4H), 2.21 (m, PCDA-H, 6H), 1.21-1.50 (m, PCDA-H, 32H), 0.89-0.91 (m, PCDA-H, 3H).

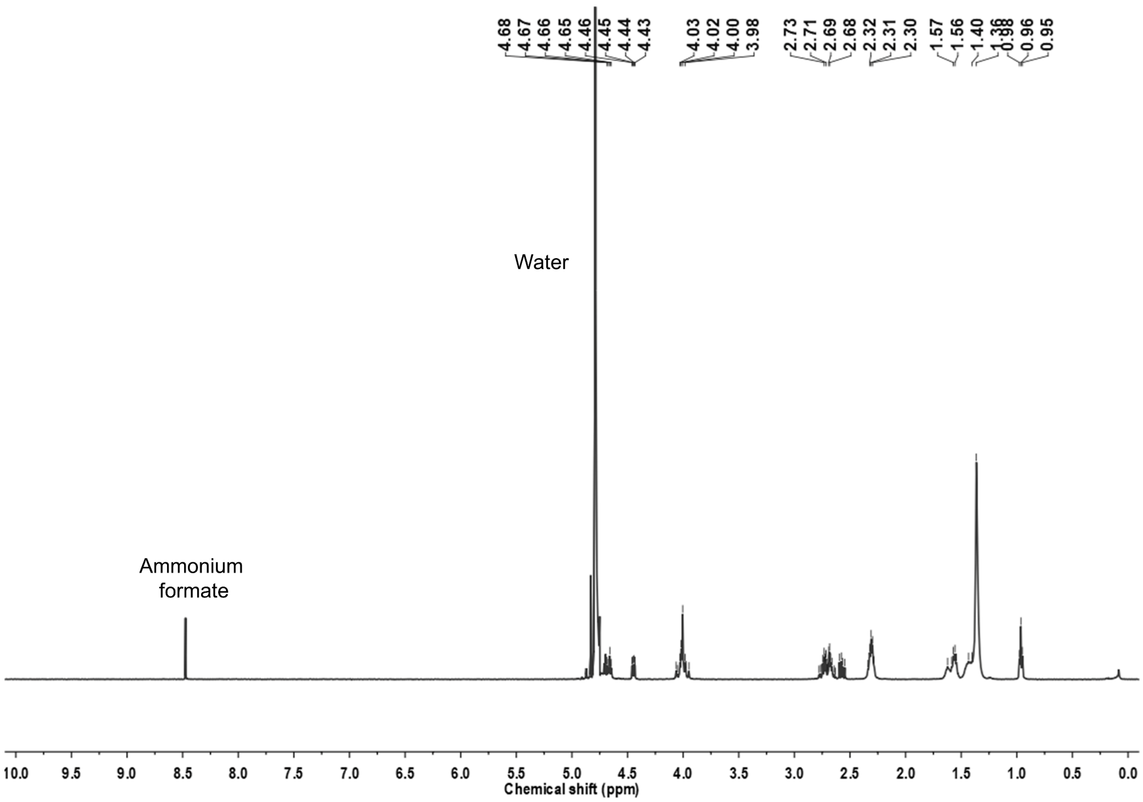

Figure S6. D**GG**DGD-DA characterization by ^{1}H-NMR (500 MHz, D_2O). δ, ppm: 4.65-4.68 (m, Asp-αH, 2H), 4.43-4.46 (q, *J* = 4.3 Hz, Asp-αH, 1H), 3.95-4.06 (m, Gly-αH, 6H), 2.55-2.78 (m, Asp-βH, 6H), 2.28-2.34 (m, PCDA-H, 6H), 1.36-1.43, 1.54-1.62 (m, PCDA-H, 32H), 0.95-0.98 (m, PCDA-H, 3H).

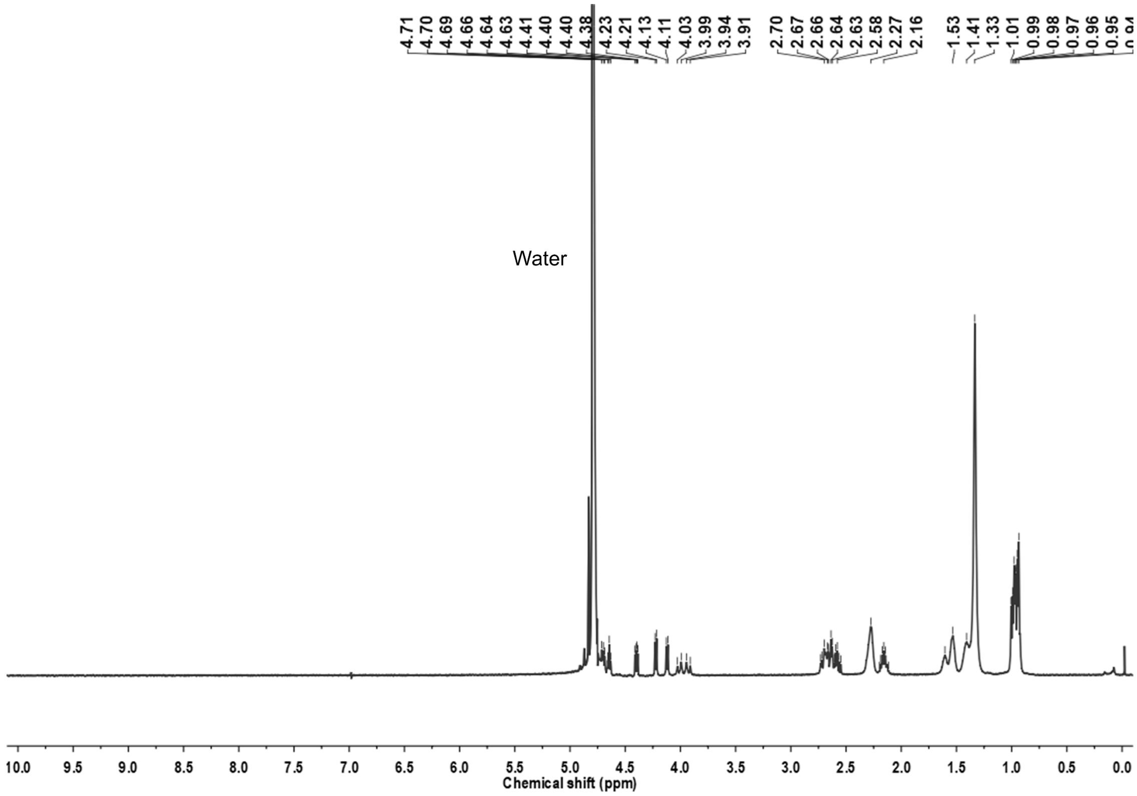

Figure S7. D**VV**DGD-DA characterization by ^{1}H-NMR (500 MHz, D_2O) δ, ppm: 4.38-4.41, 4.63-4.66, 4.69-4.71 (m, Asp-αH, 3H), 4.21-4.23 (m, Val-αH, 1H),, 4.11-4.13 (d, *J* = 7.8 Hz, Val-αH, 1H), 3.91-4.03 (m ,Gly-αH, 2H), 2.55-2.73 (s, Asp-βH, 6H), 2.11-2.20, 2.27 (m, PCDA-H, 6H), 1.33-1.60 (m, PCDA-H, 32H), 0.94-1.01 (m, PCDA-H, 3H; Val-γH, 12H).

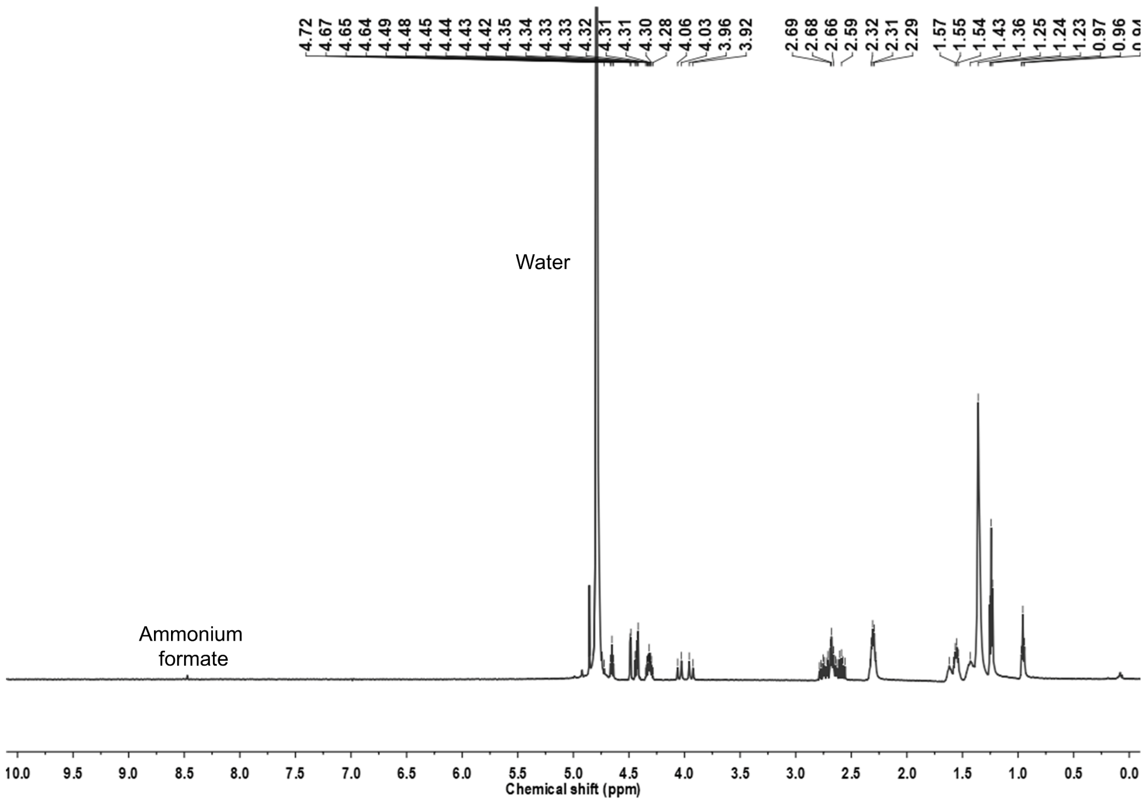

Figure S8. D**TT**DGD-DA characterization by ^{1}H-NMR (500 MHz, D_2O) δ, ppm: 4.72 (m, Asp-αH, 1H), 4.64-4.67 (t, *J* =6.55 Hz , Asp-αH, 1H) 4.48-4.49 (d, *J* = 9.58 Hz, Asp-αH, 1H), 4.28-4.37, 4.42-4.45 (m, Thr-αH, 2H), 4.03-4.06 (d, *J* =17 Hz, Gly-αH, 1H), 3.92-3.96 (d, *J* = 17.1 Hz, Gly-αH, 1H), 2.56-2.79 (m, Asp-βH, 6H), 2.28-2.33 (m, PCDA-H, 6H), 1.36-1.62 (m, PCDA-H, 32H), 1.23-1.25 (m, Thr-γH, 6H), 0.94-0.97 (t, *J* = 6.73 Hz, PCDA-H, 3H).

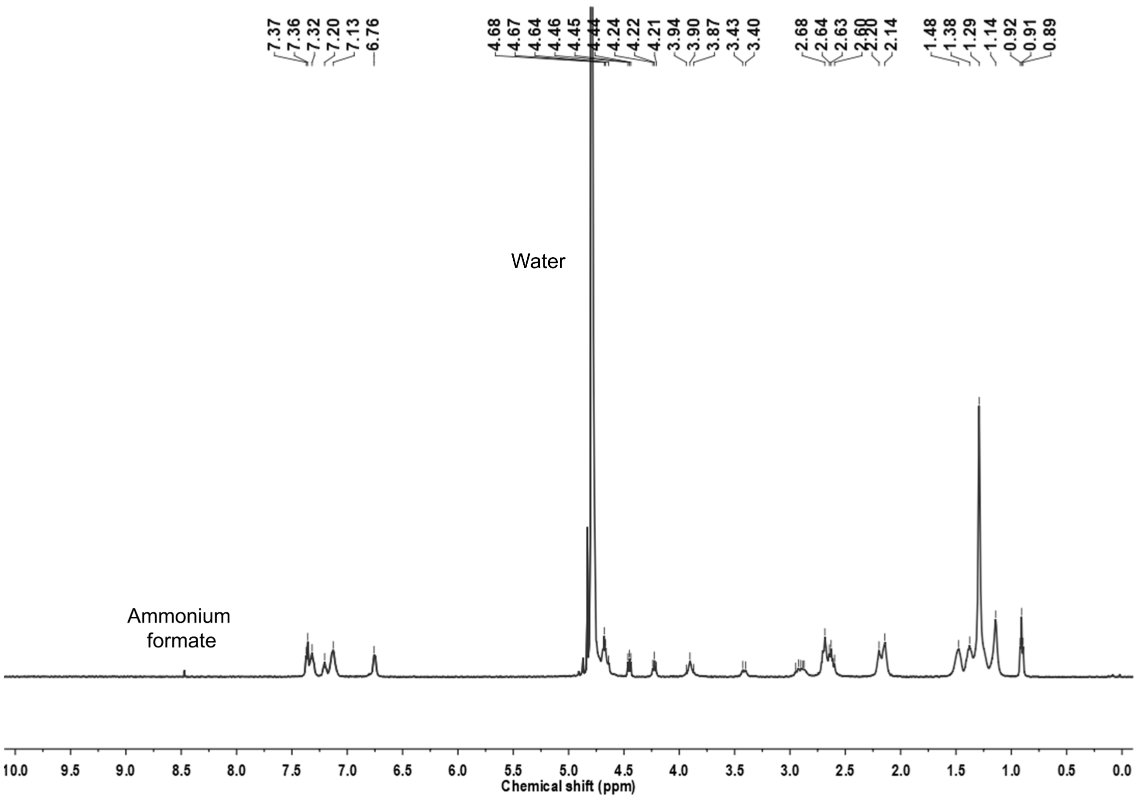

Figure S9. D**FF**DGD-DA characterization by [1] H-NMR (500 MHz, D_2O) δ, ppm: 7.32-7.37 (m, Phe-3, 5H, 4H), 7.13-7.20 (m, Phe-2, 6H, 4H), 6.76 (m, Phe-4H, 2H), 4.64-4.68(m, Asp-αH, 3H), 4.44-4.46 (t, *J* = 6.80 Hz, Phe-αH, 1H), 4.21-4.24 (t, *J* =7.88 Hz, Phe-αH, 1H), 3.87-3.94 (t, *J* = 16.88 Hz, Gly-αH, 2H), 3.40-3.43 (d, *J* = 12.60 Hz, Phe-βH, 1H,) 2.87-2.95 (m, Phe-βH, 2H,) 2.60-2.68 (m, Phe-βH, 1H, Asp-βH, 6H), 2.14-2.20 (m, PCDA-H, 6H), 1.14-1.48 (m, PCDA-H, 32H), 0.89-0.92 (t, *J* = 6.38 Hz, PCDA-H, 3H).

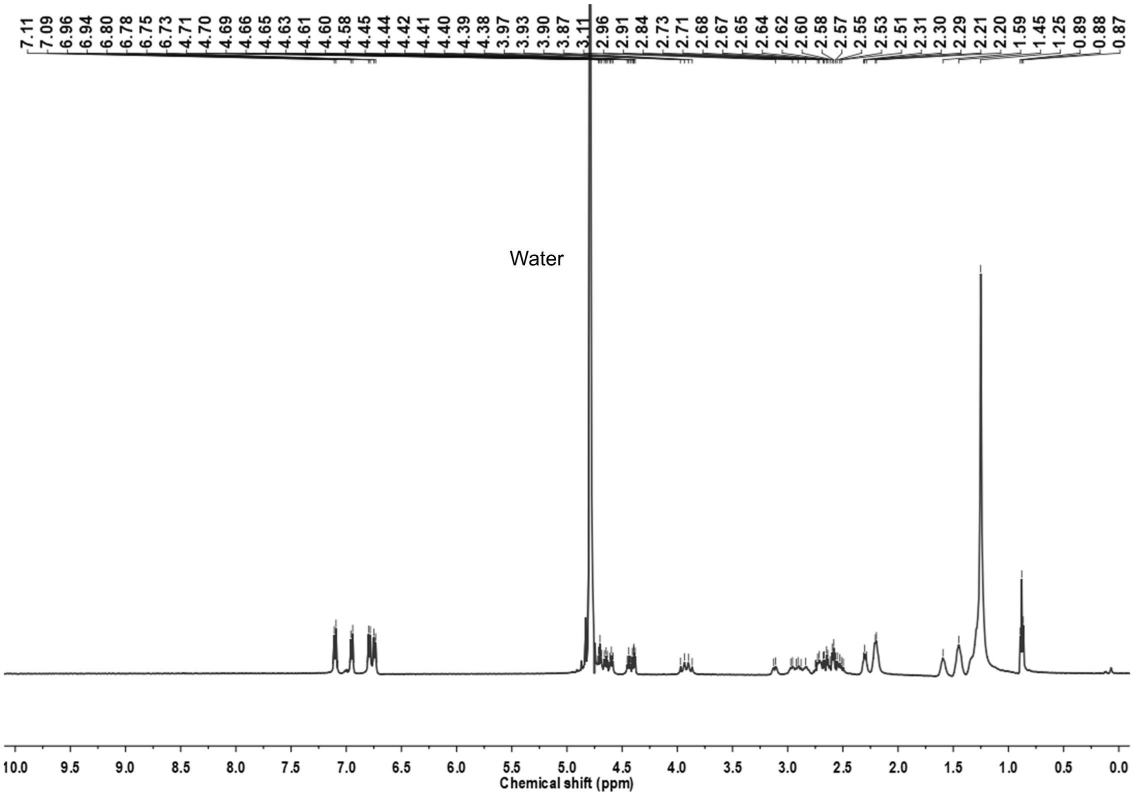

Figure S10. D**YY**DGD-DA characterization by [1] H-NMR (500 MHz, D_2O) δ, ppm: 6.94-6.96, 7.08-7.11 (m, Tyr-2,6H, 4H), 6.73-6.80 (m, Tyr-3,5H, 4H), 4.69-4.71 (t, *J* = 6.58 Hz, Asp-αH, 1H), 4.63-4.66 (t, *J* = 6.95 Hz, Asp-αH, 1H), 4.58-4.61 (t, *J* = 7.05 Hz, Asp-αH, 1H), 4.38-4.45 (m, Tyr-αH, 2H), 3.87-3.97 (q, *J* = 17.65 Hz, Gly-αH, 2H), 2.84-2.97, 3.11-3.13 (m, Tyr-βH, 4H), 2.50-2.75 (m, Asp-βH, 6H), 2.20-2.21, 2.29-2.30 (m, PCDA-H, 6H), 1.25-1.59 (m, PCDA-H, 32H), 0.87-0.89 (t, *J* = 6.90 Hz, PCDA-H, 3H).

Characterization of peptide-DA monomers by ESI-MS

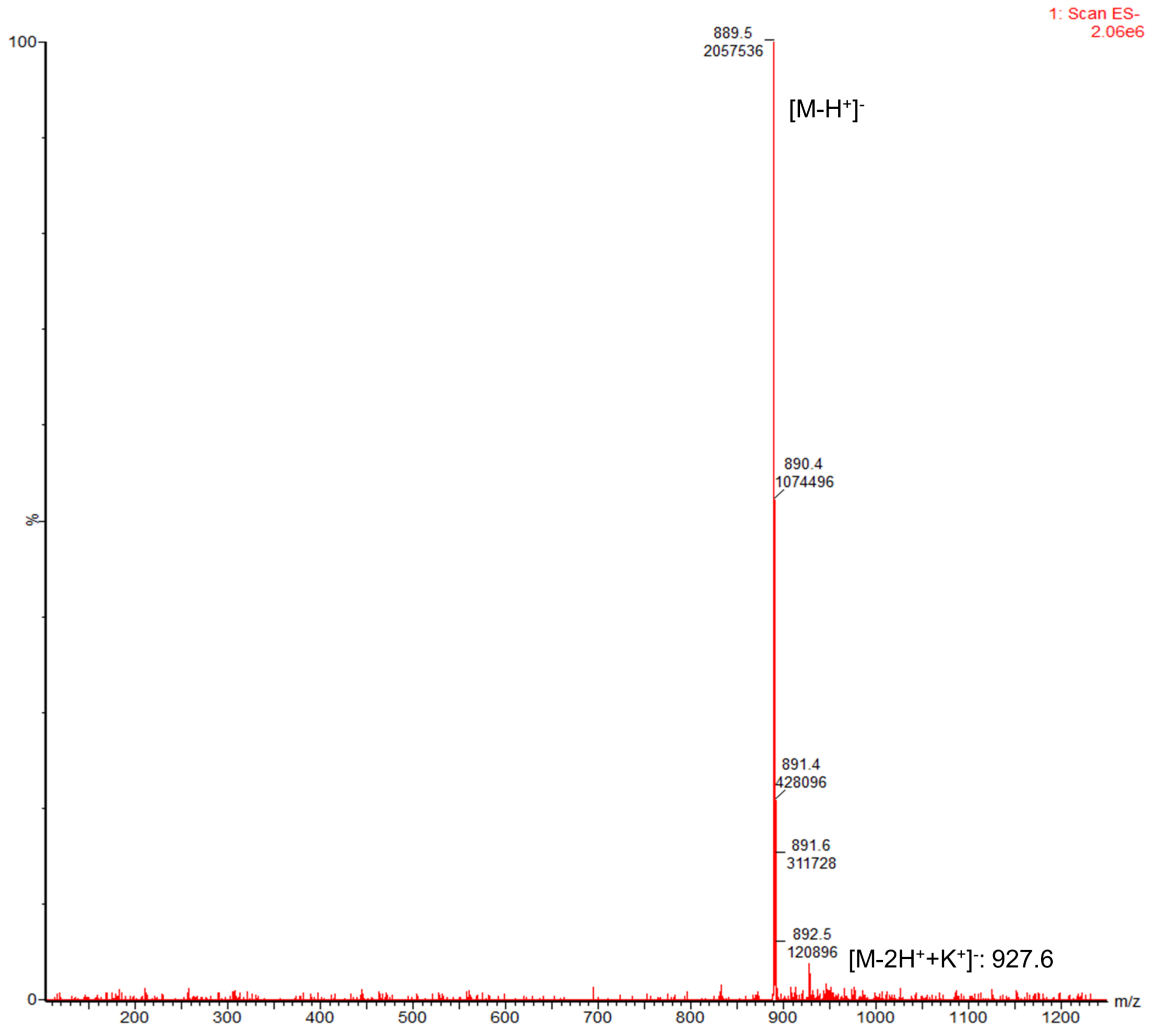

Figure S11. DDGD**GG**-DA characterization by ESI-MS.

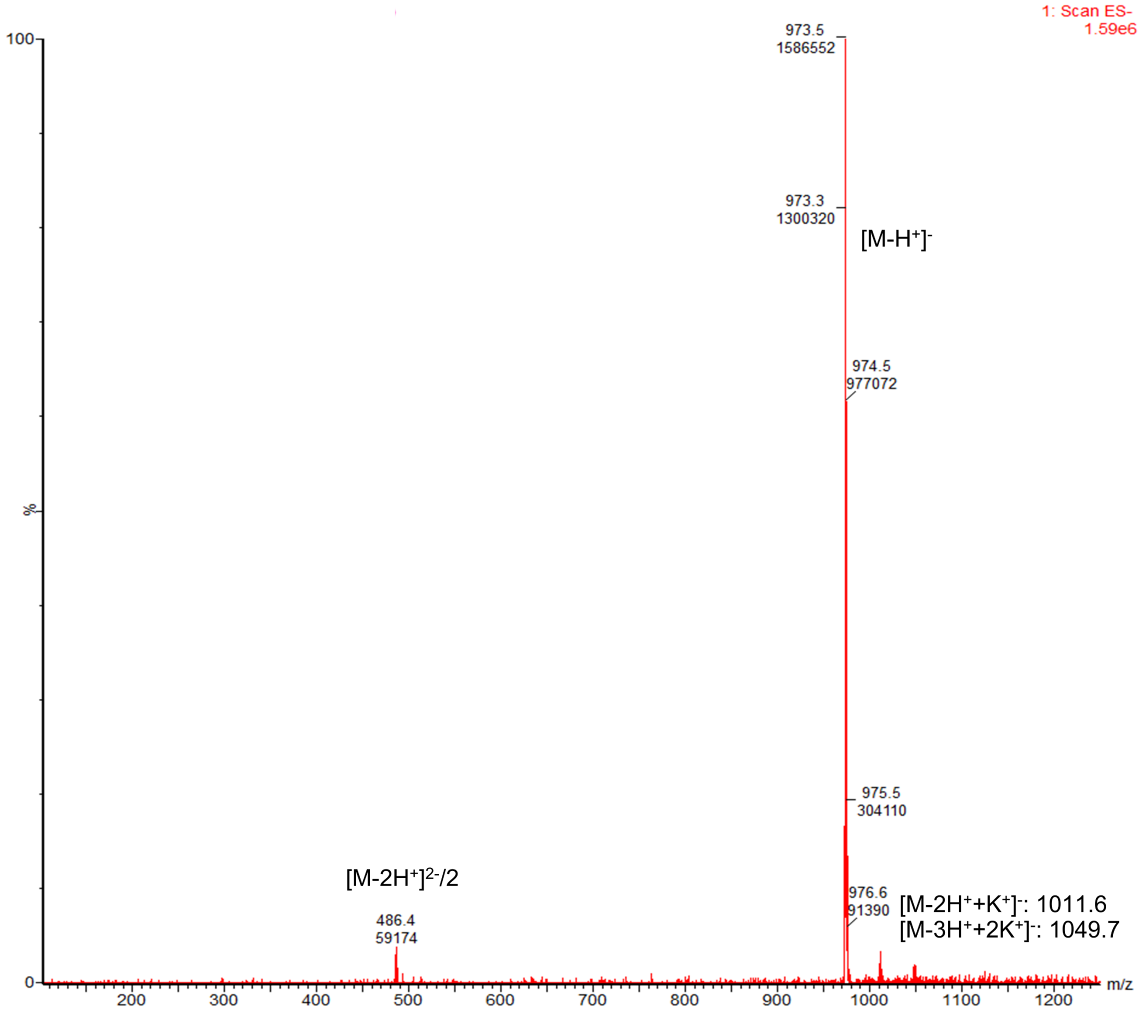

Figure S12. DDGD**VV**-DA characterization by ESI-MS.

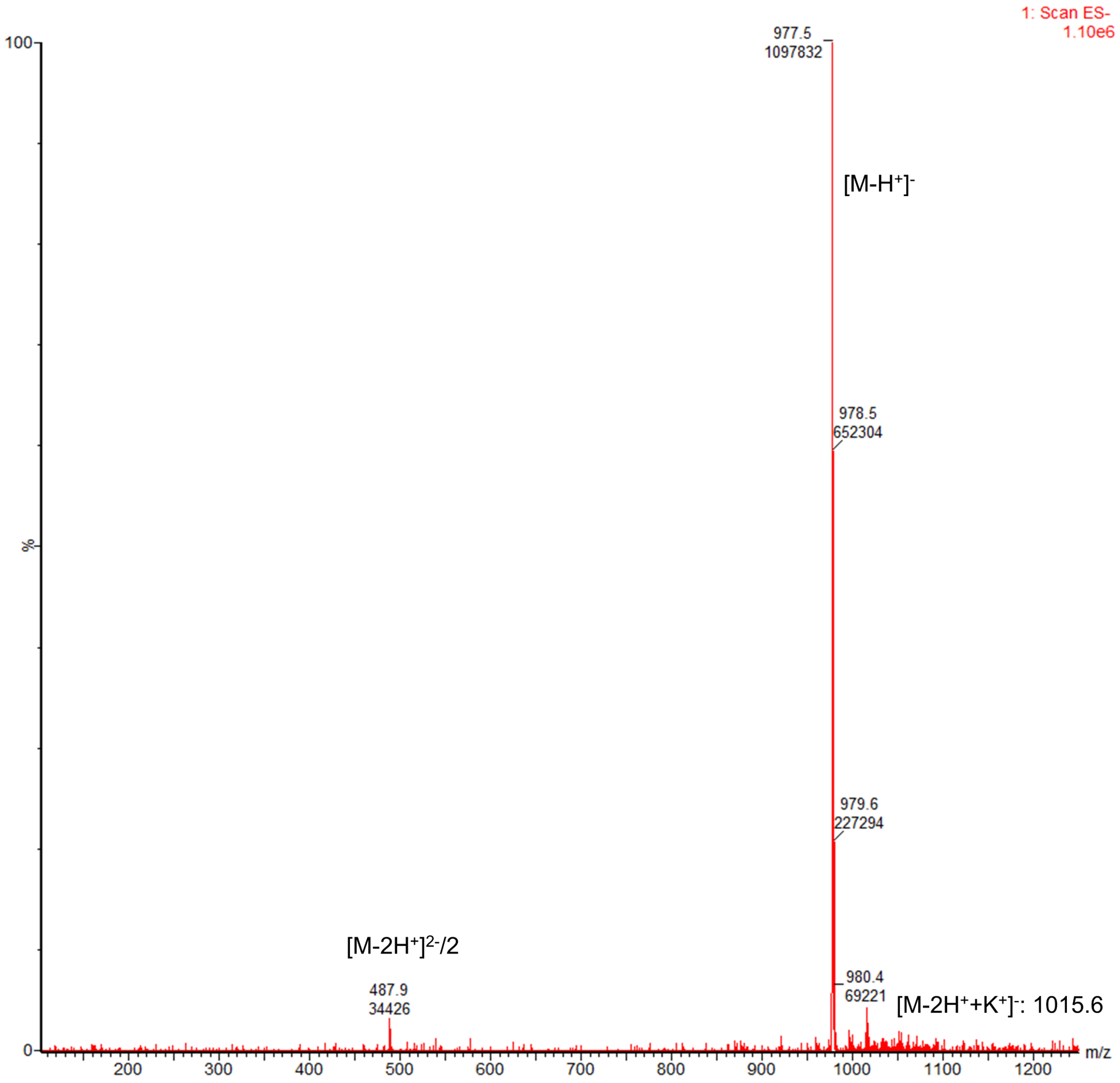

Figure S13. DDGD**TT**-DA characterization by ESI-MS.

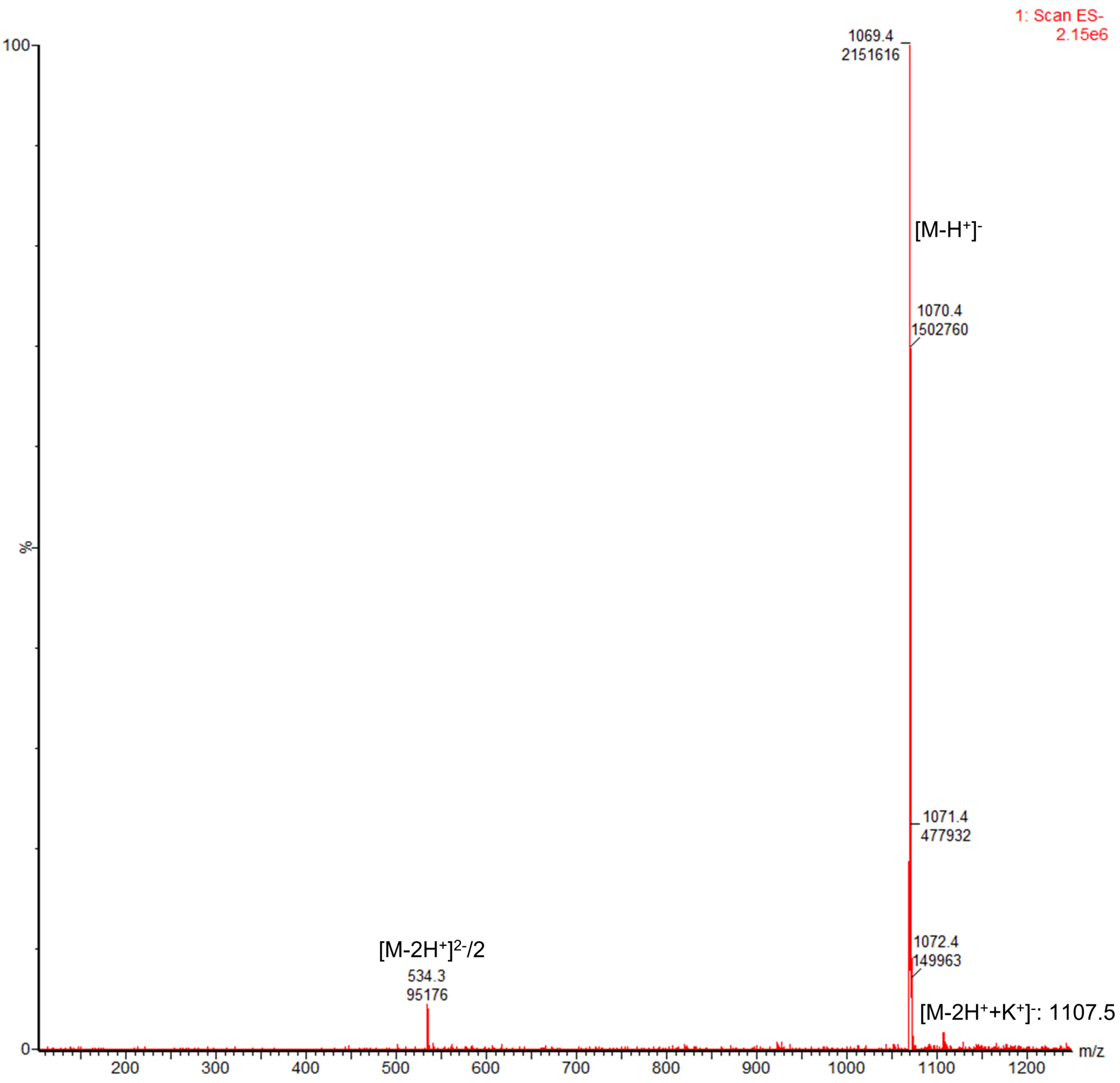

Figure S14. DDGD**FF**-DA characterization by ESI-MS.

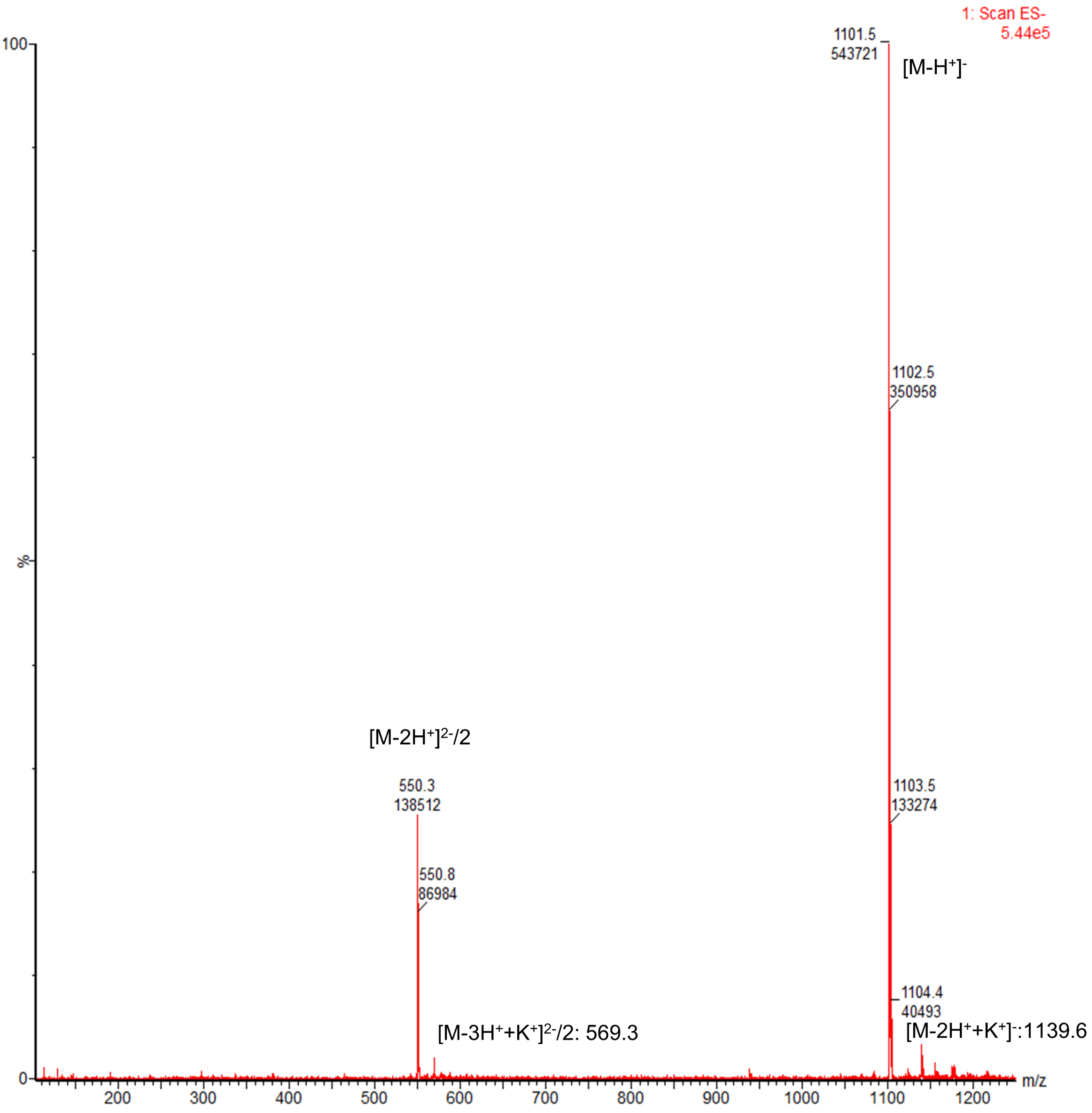

Figure S15. DDGD**<u>YY</u>**-DA characterization by ESI-MS.

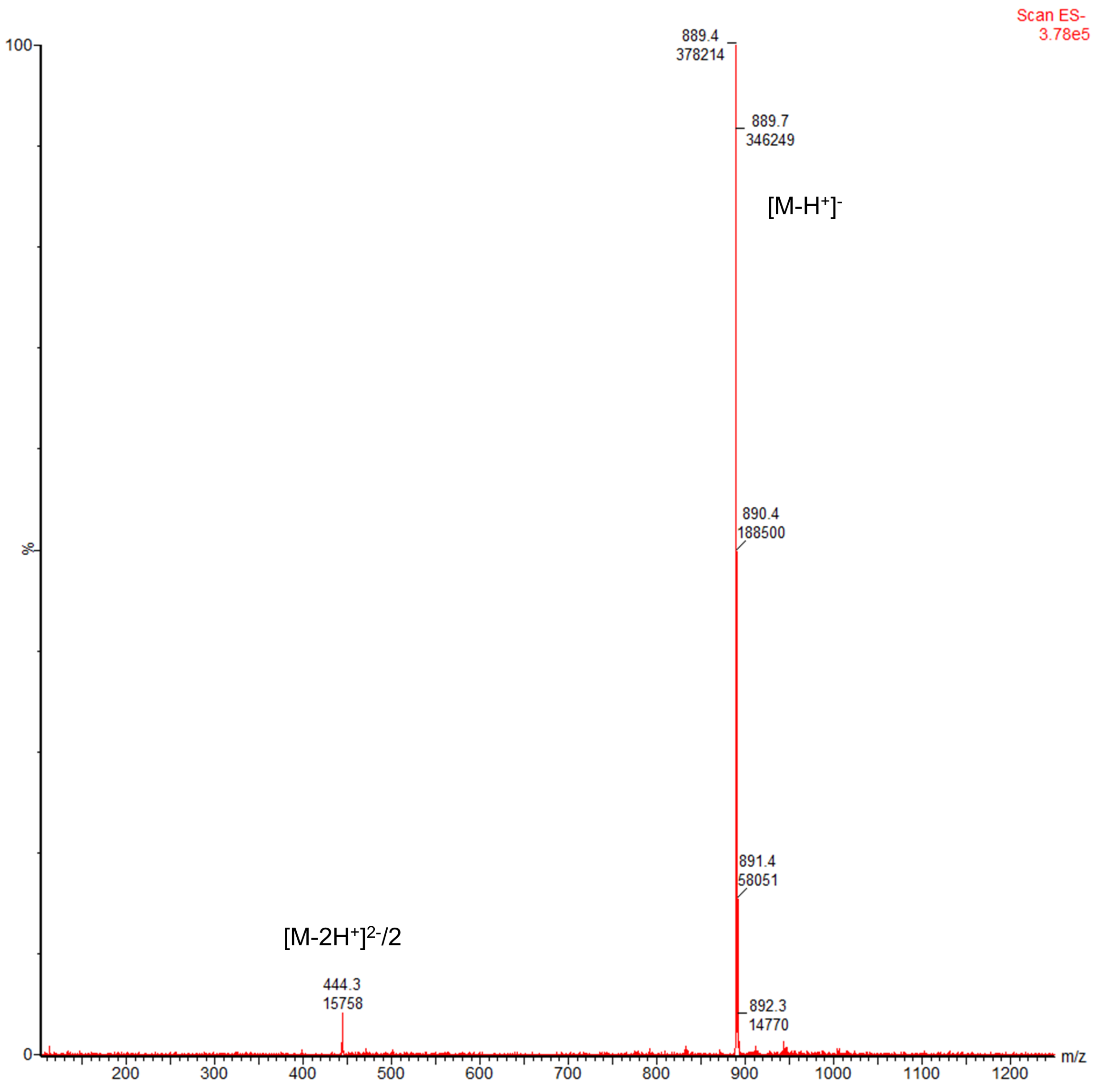

Figure S16. D**GG**DGD-DA characterization by ESI-MS.

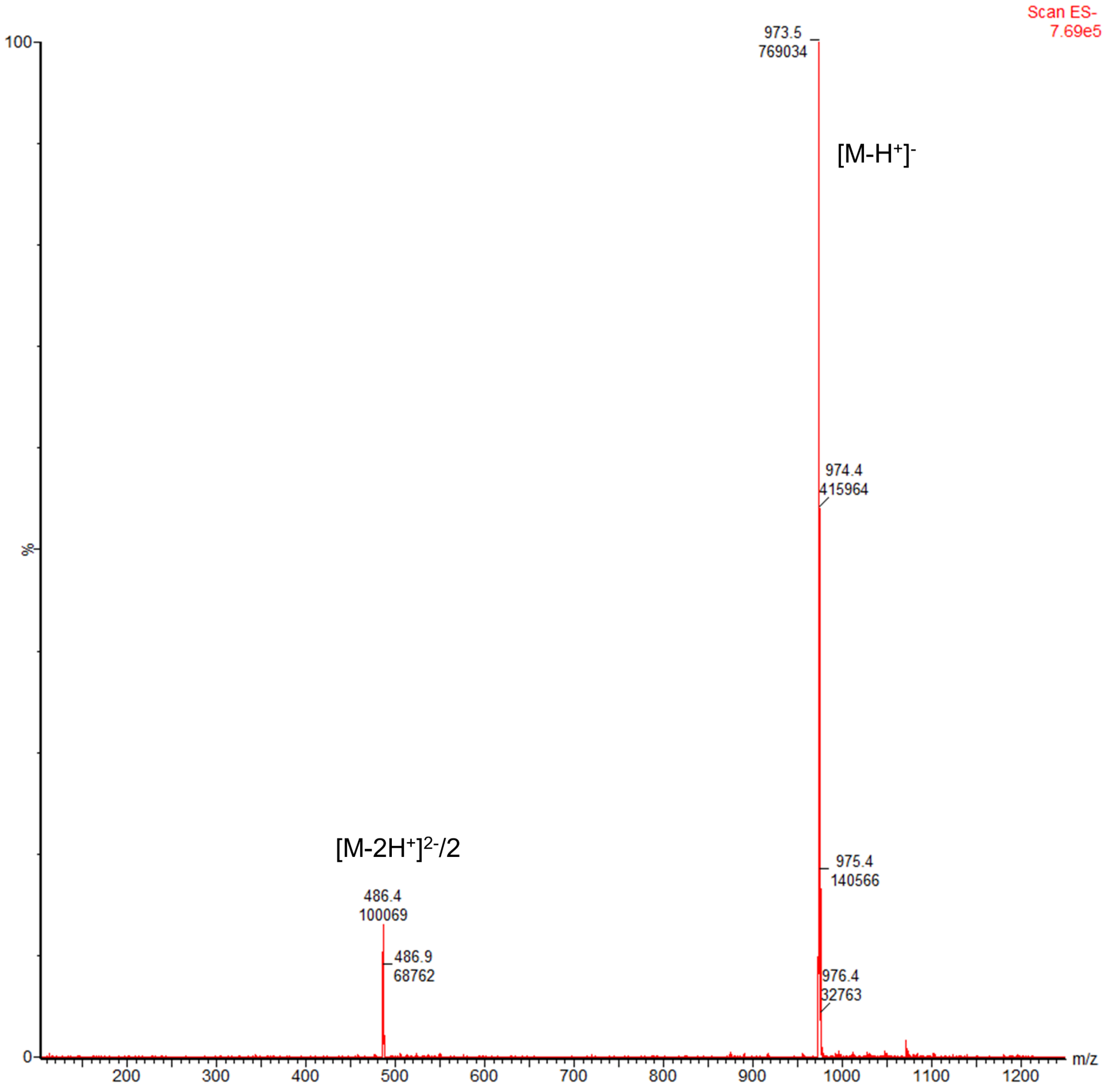

Figure S17. D<u>**VV**</u>DGD-DA characterization by ESI-MS.

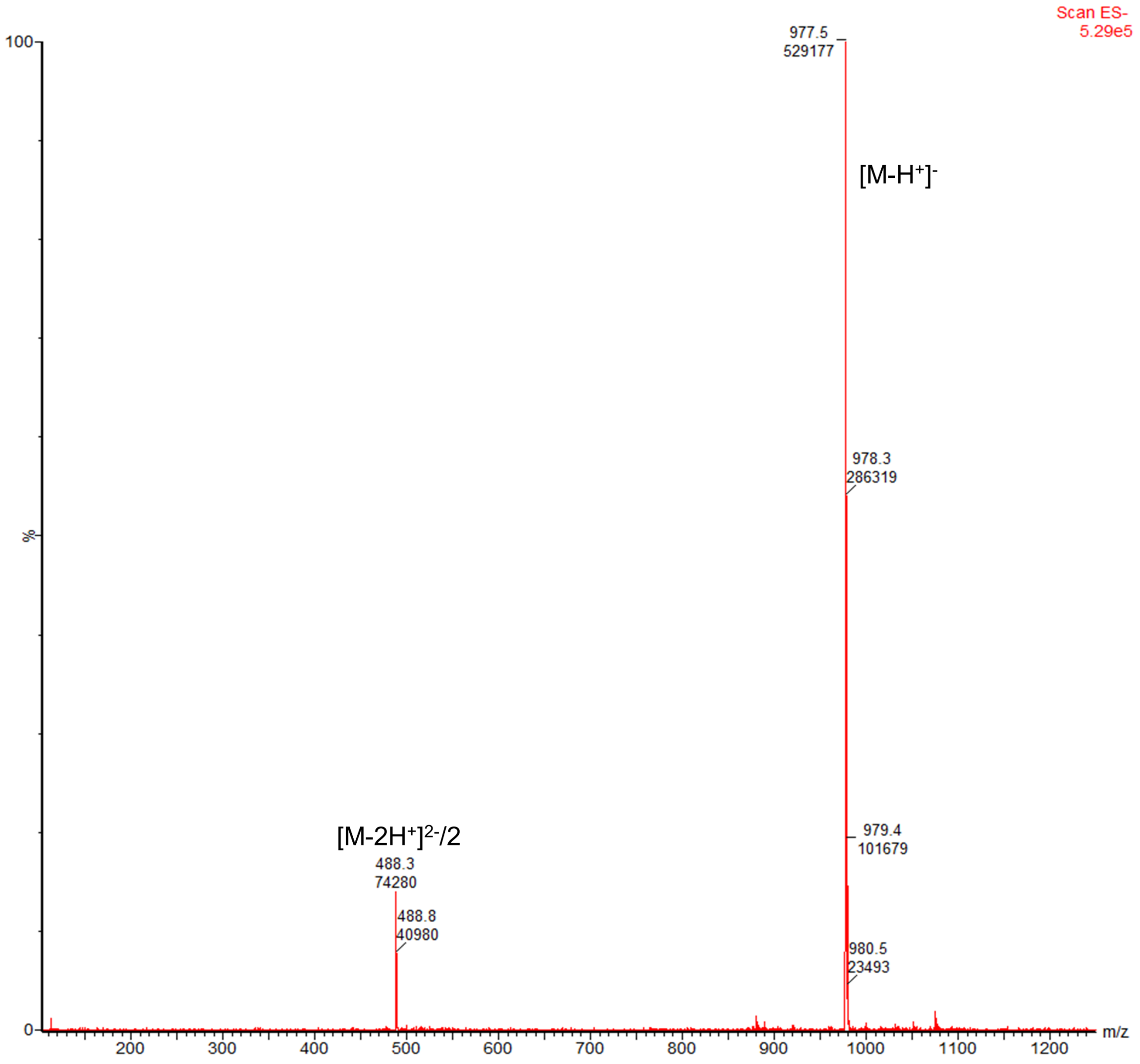

Figure S18. D<u>**TT**</u>DGD-DA characterization by ESI-MS.

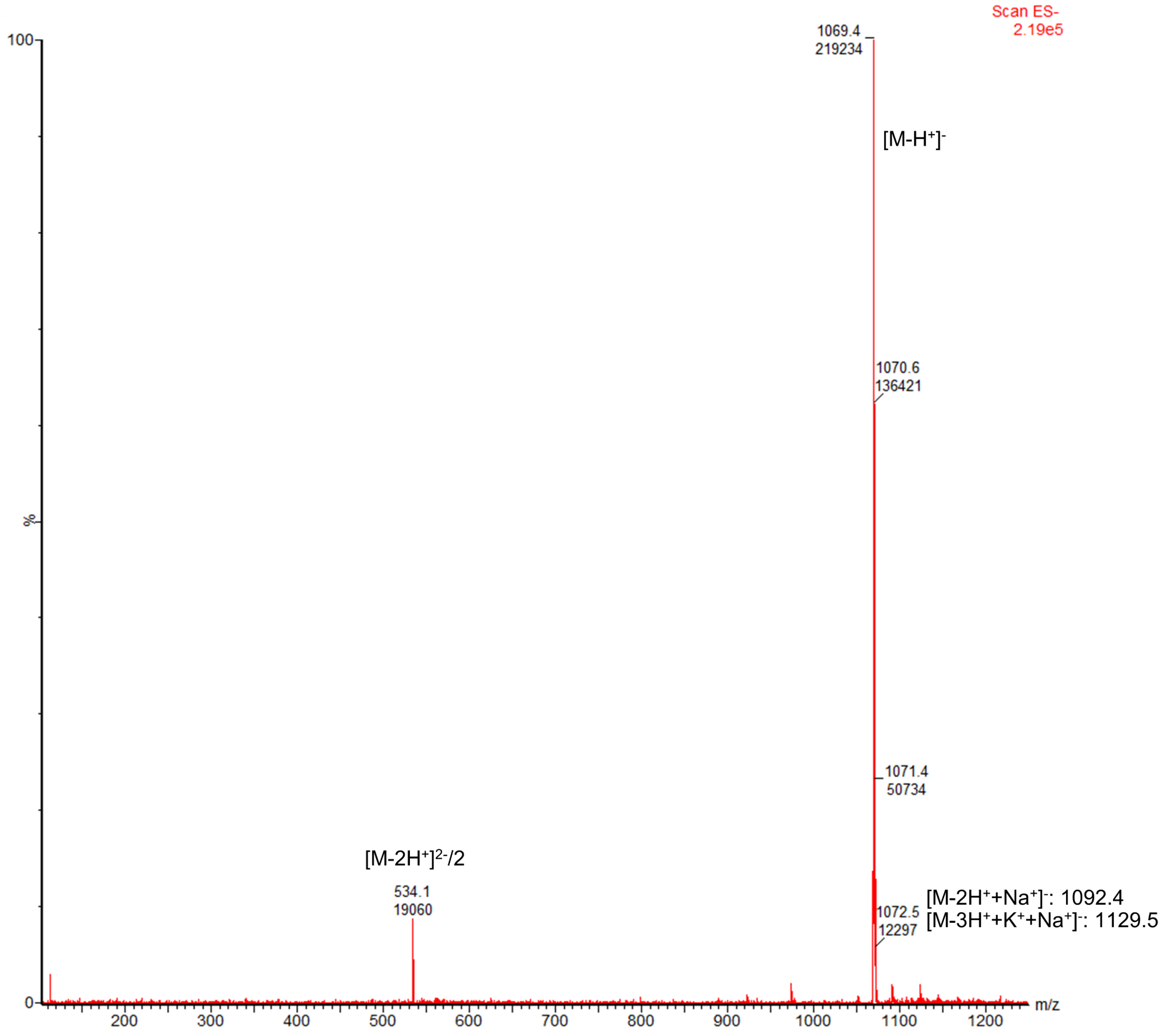

Figure S19. D**<u>FF</u>**DGD-DA characterization by ESI-MS.

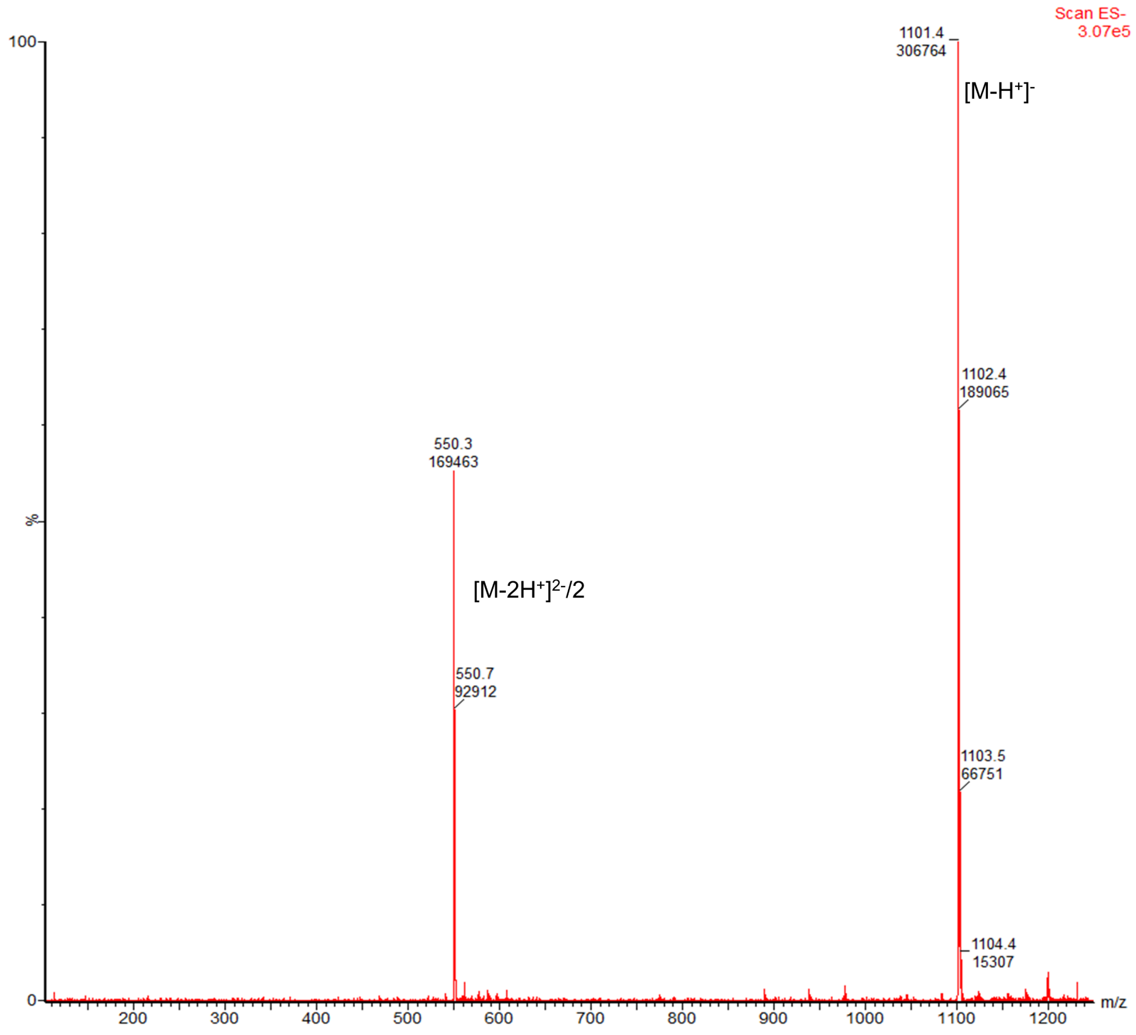

Figure S20. D<u>**YY**</u>DGD-DA characterization by ESI-MS.

Characterization of peptide-DA monomers with analytical HPLC

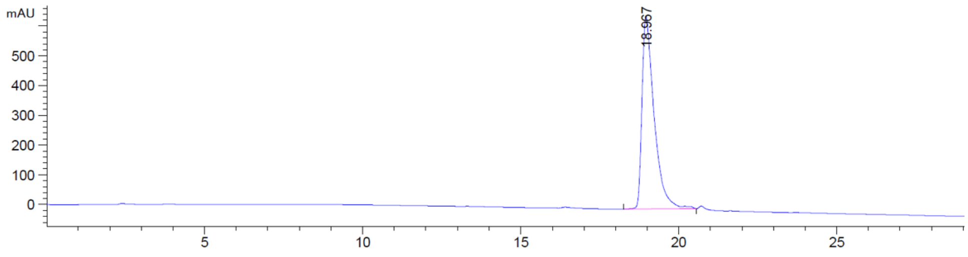

Figure S21. DDGD**GG**-DA characterization by analytical HPLC aSt 220 nm.

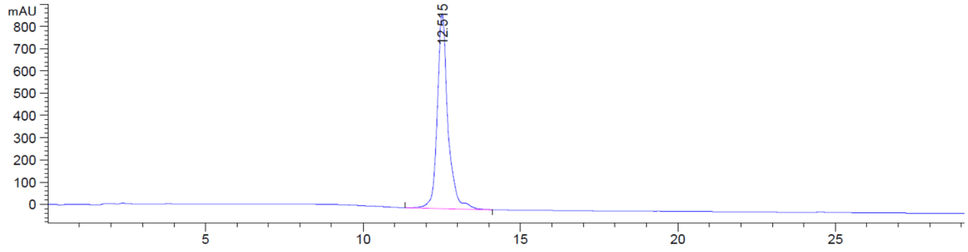

Figure S22. DDGD**VV**-DA characterization by analytical HPLC at 220 nm.

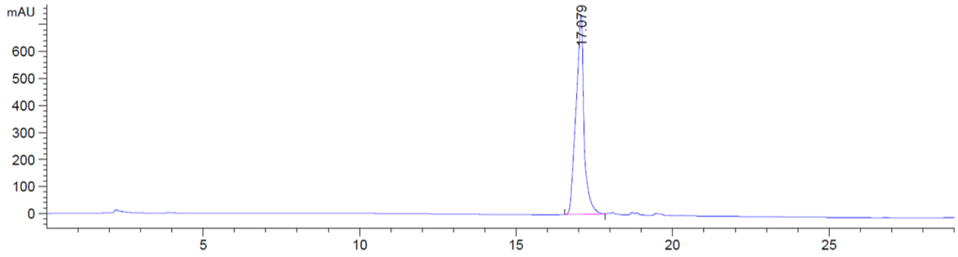

Figure S23. DDGD**TT**-DA characterization by analytical HPLC at 220 nm.

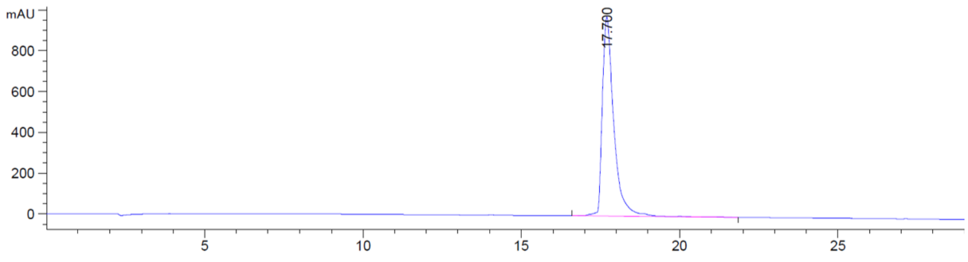

Figure S24. DDGD**FF**-DA characterization by analytical HPLC at 220 nm.

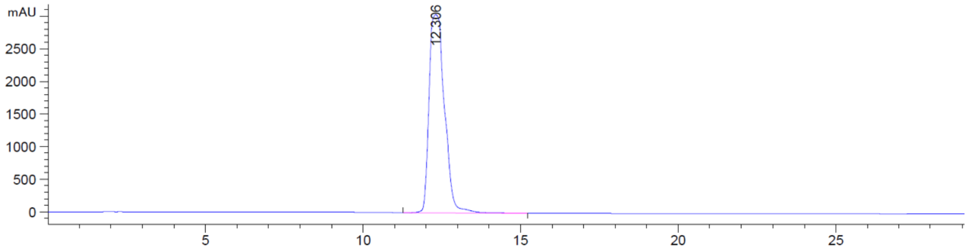

Figure S25. DDGD**YY**-DA characterization by analytical HPLC at 220 nm.

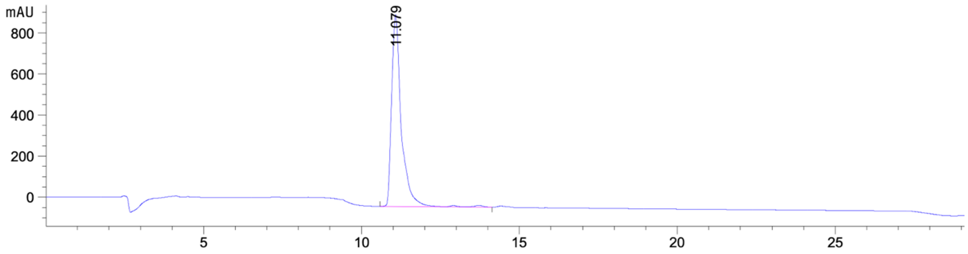

Figure S26. D**GG**DGD-DA characterization by analytical HPLC at 220 nm.

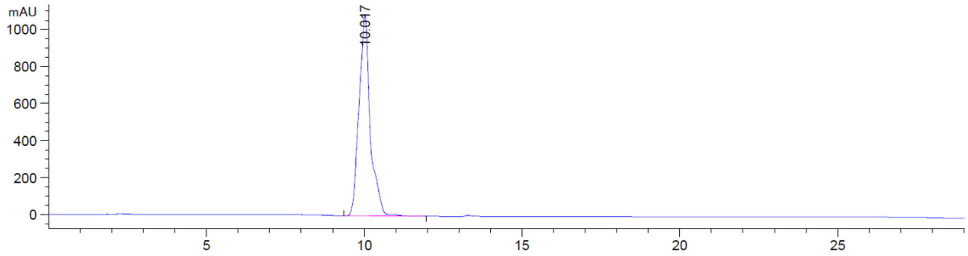

Figure S27. D**VV**DGD-DA characterization by analytical HPLC at 220 nm.

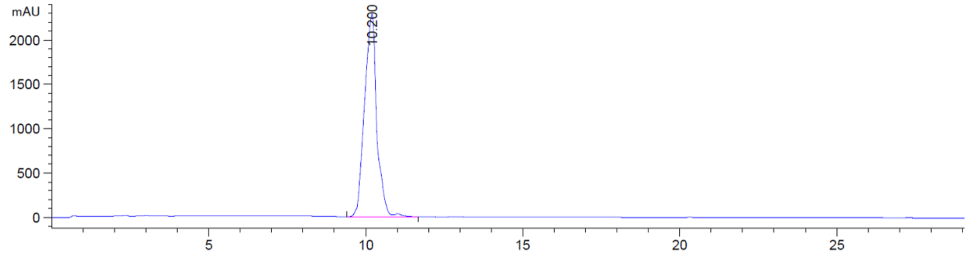

Figure S28. D**TT**DGD-DA characterization by analytical HPLC at 220 nm.

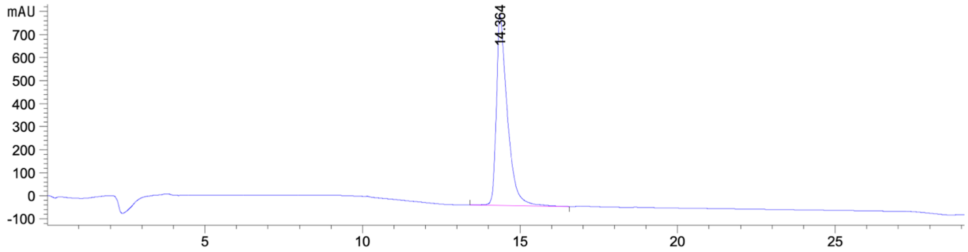

Figure S29. D**FF**DGD-DA characterization by analytical HPLC at 220 nm.

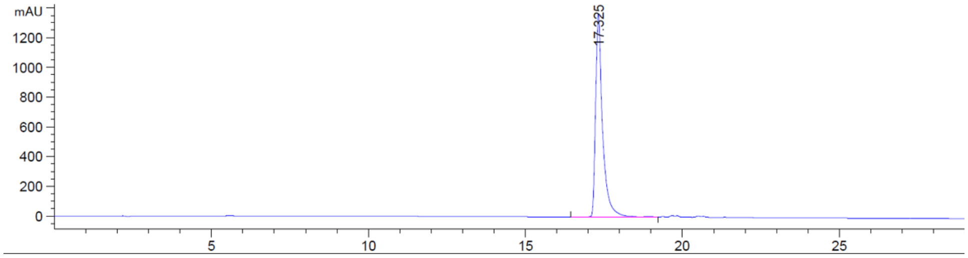

Figure S30. D**YY**DGD-DA characterization by analytical HPLC at 220 nm.

NMR characterization of synthesized peptide-PDA polymers

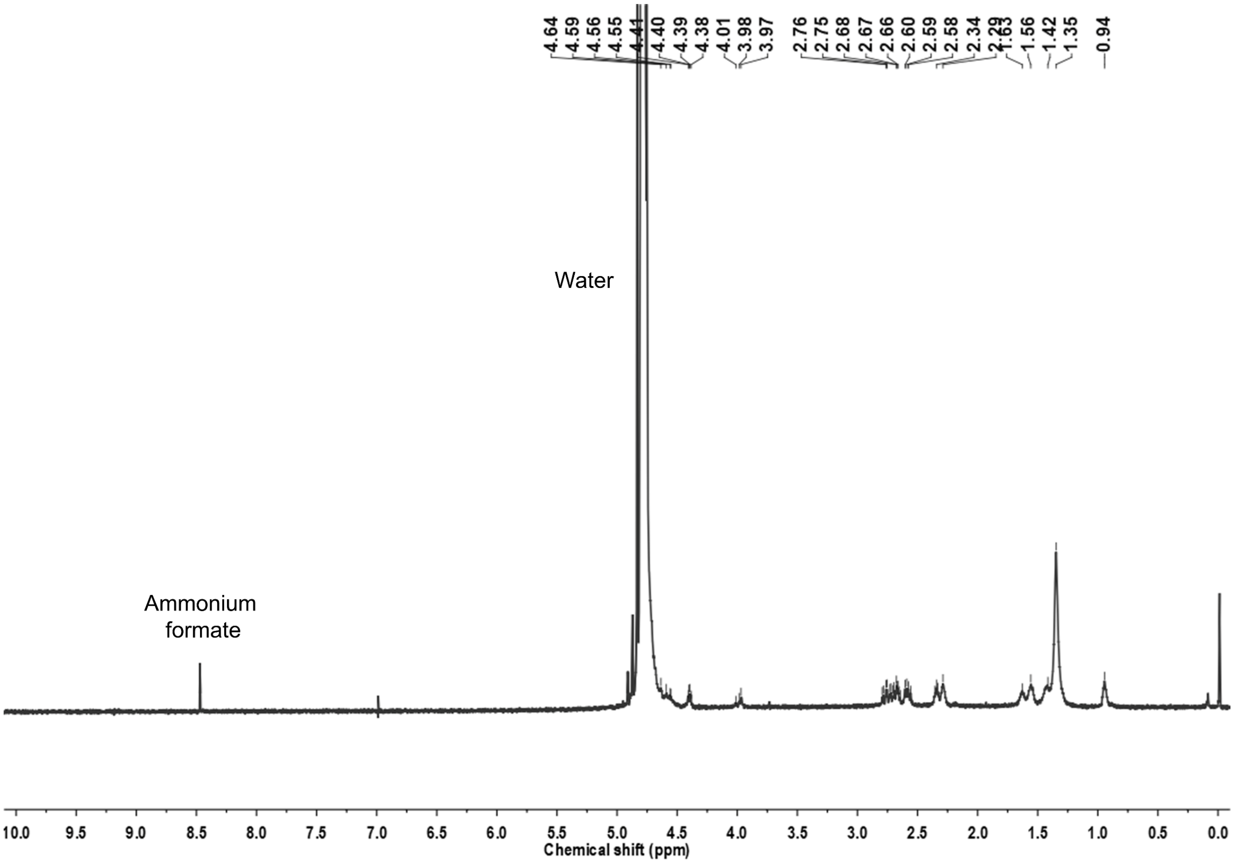

Figure S31. DDGD**GG**-PDA characterization by [1] H-NMR (500 MHz, D_2O) δ, ppm: 4.38-4.41, 4.55-4.64 (m, Asp-αH, 3H), 3.97-4.01 (m, Gly-αH, 6H), 2.56-2.79 (m, Asp-βH, 6H), 2.29-2.35 (m, PDA-H, 6H), 1.35-1.63 (m, PDA-H, 32H), 0.94 (s, PDA-H, 3H).

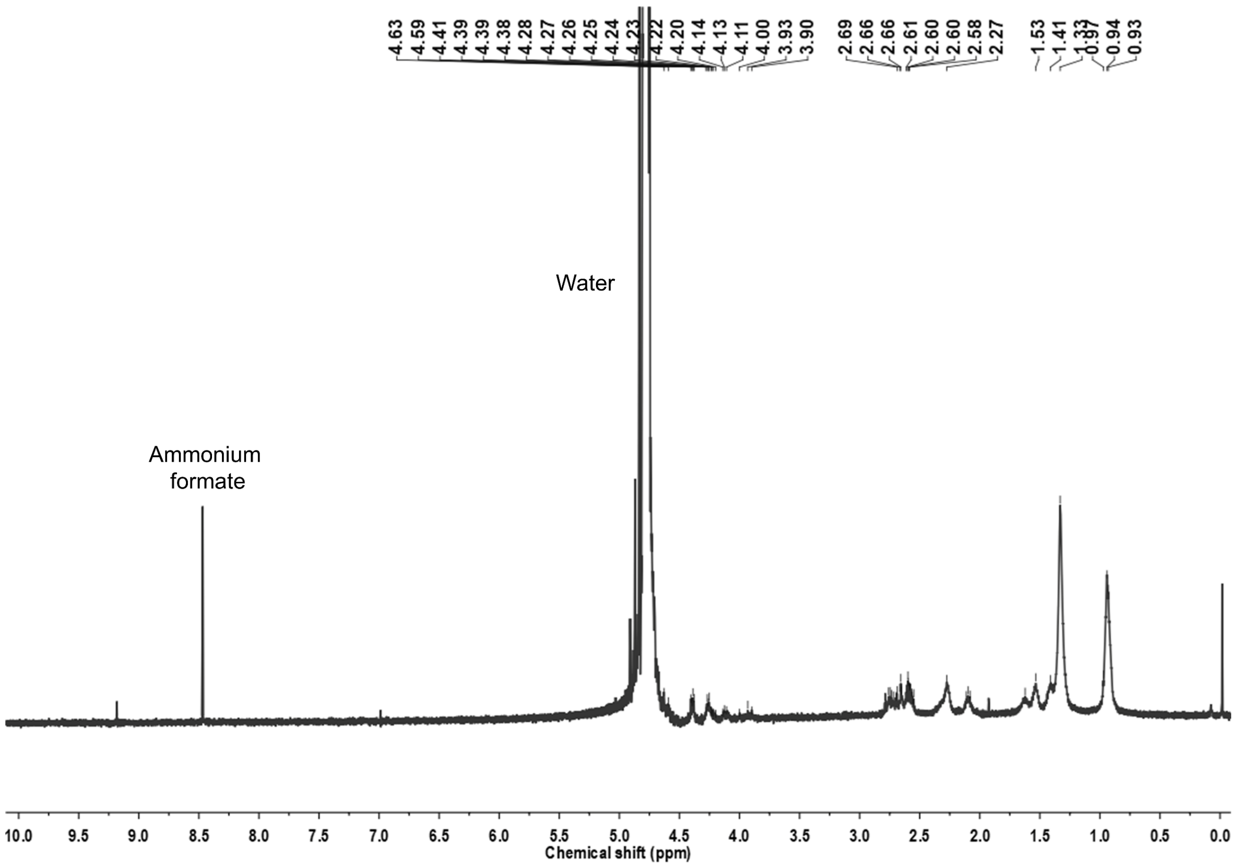

Figure S32. DDGD<u>**VV**</u>-PDA characterization by ^{1}H-NMR (500 MHz, D_2O) δ, ppm: 4.39-4.41, 4.59-4.63 (m, Asp-αH, 3H), 4.20-4.28 (m, Val-αH, 2H), 3.90-4.14 (m, Gly-αH, 2H), 2.55-2.79 (m, Asp-βH, 6H), 2.08-2.27 (m, PDA-H, 6H), 1.33-1.62 (m, PDA-H, 32H), 0.93-0.97 (m, PDA-H, 3H; Val-γH, 12H).

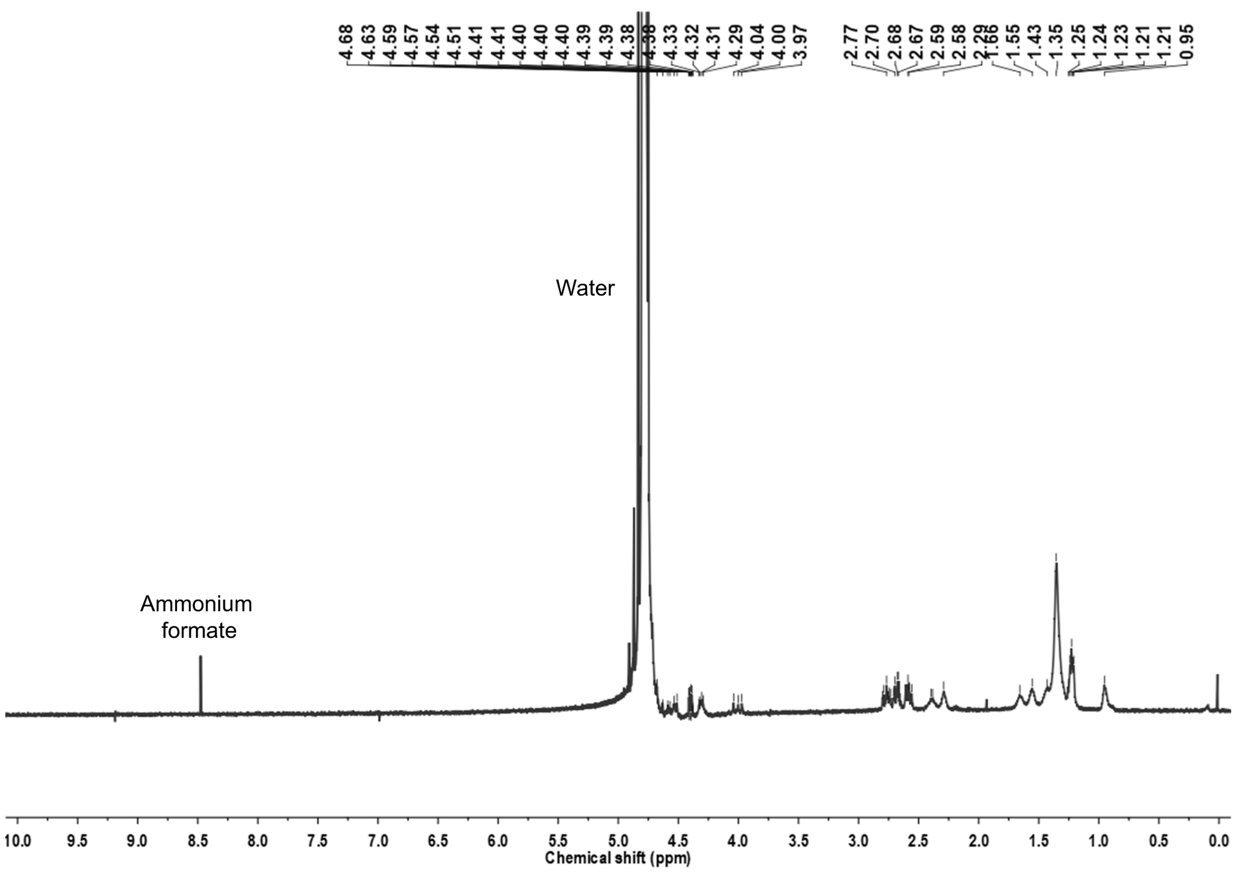

Figure S33. DDGD**TT**-PDA characterization by ^{1}H-NMR (500 MHz, D_2O) δ, ppm: 4.38-4.41, 4.51-4.68, (m, Asp-αH, 3H), 4.29-4.33 (m, Thr-αH, 2H), 3.97-4.04 (m, Gly-αH, 2H), 2.56-2.80 (m, Asp-βH, 6H), 2.29-2.40 (m, PDA-H, 6H), 1.35-1.66 (m, PDA-H, 32H), 1.21-1.25 (m, Thr-γH, 6H), 0.95 (s, PDA-H, 3H).

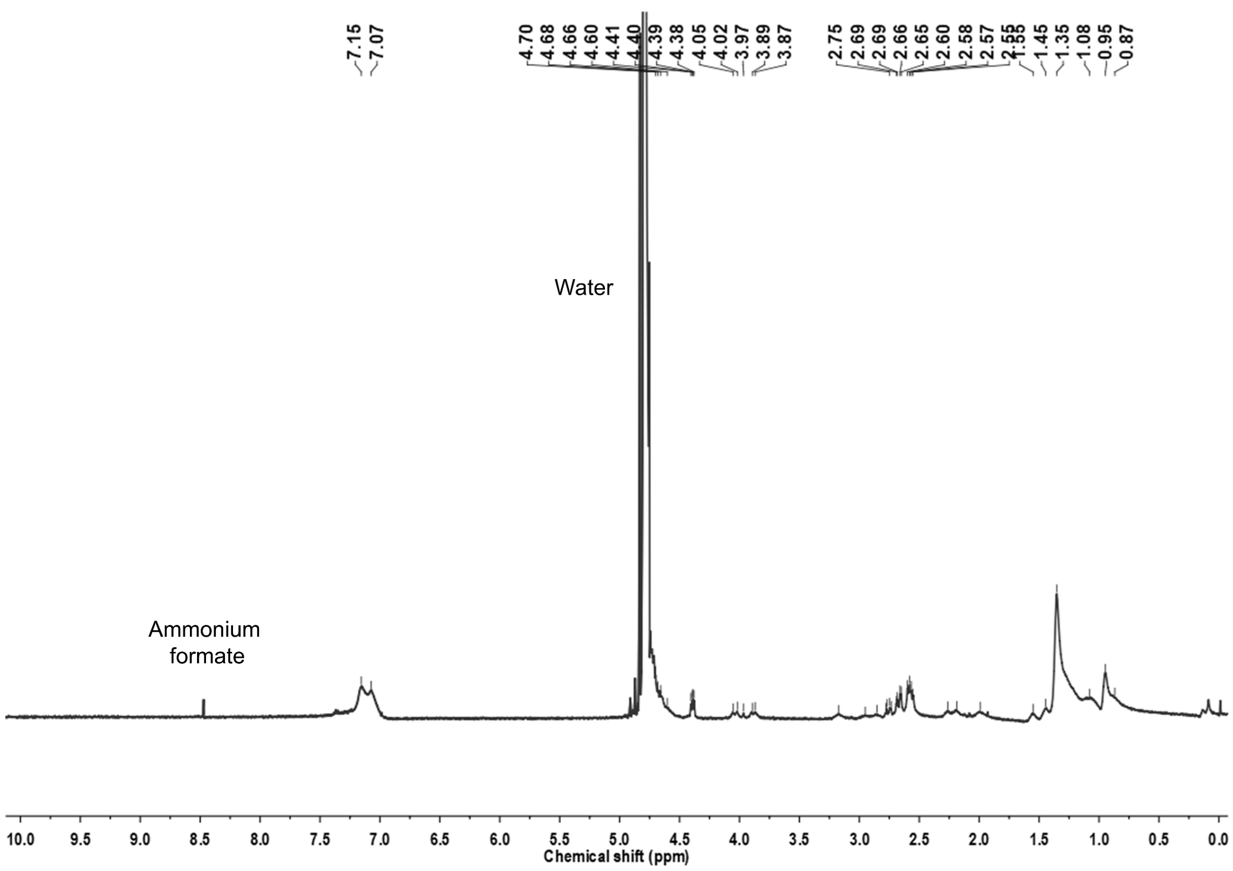

Figure S34. DDGD**FF**-PDA characterization by [1] H-NMR (500 MHz, D_2O) δ, ppm: 7.07-7.15 (m, Phe-aromatic H, 10H), 4.38-4.41, 4.60-4.70 (m, Asp-αH, 3H, Phe-αH, 2H), 3.87-4.05 (m, Gly-αH, 2H), 2.85-2.95, 3.17 (m, Phe-βH, 4H), 2.55-2.78 (Asp-βH, 6H), 2.19-2.26 (m, PDA-H, 4H), 1.99 (s, PDA-H, 2H), 1.08-1.55 (m, PDA-H, 32H), 0.87-0.95 (m, PDA-H, 3H).

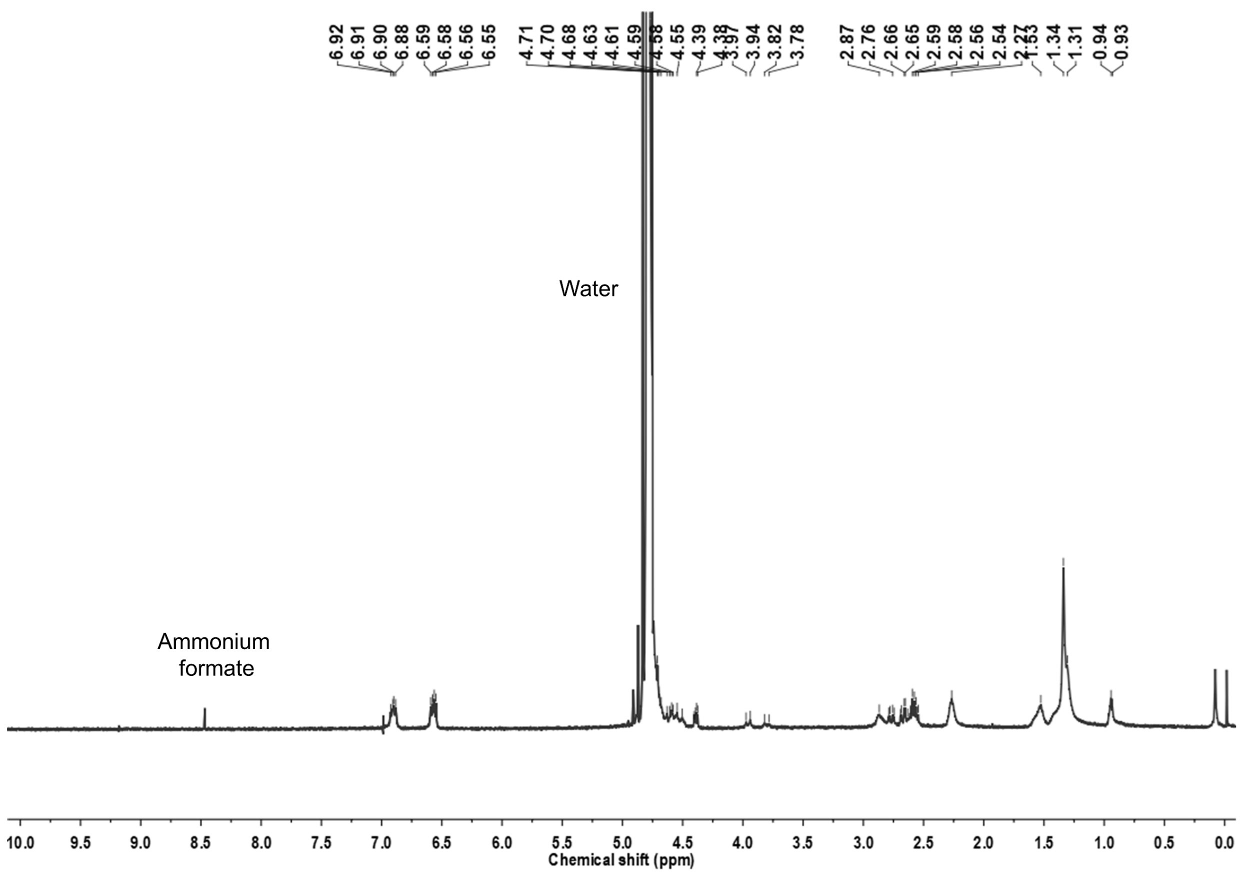

Figure S35. DDGD<u>**YY**</u>-PDA characterization by ^{1}H-NMR (500 MHz, D_2O) δ, ppm: 6.88-6.92 (m, Tyr-2,6H, 4H), 6.55-6.59 (m, Tyr-3,5H, 4H), 4.50-4.71 (m, Asp-αH, 3H, Tyr-αH, 2H), 3.78-3.97, 4.38-4.40, (m, Gly-αH, 2H), 2.54-2.87 (m, Asp-βH, 6H, Tyr-βH, 4H), 2.27 (m, PDA-H, 6H), 1.31-1.53 (m, PDA-H, 32H), 0.93-0.94 (m, PDA-H, 3H).

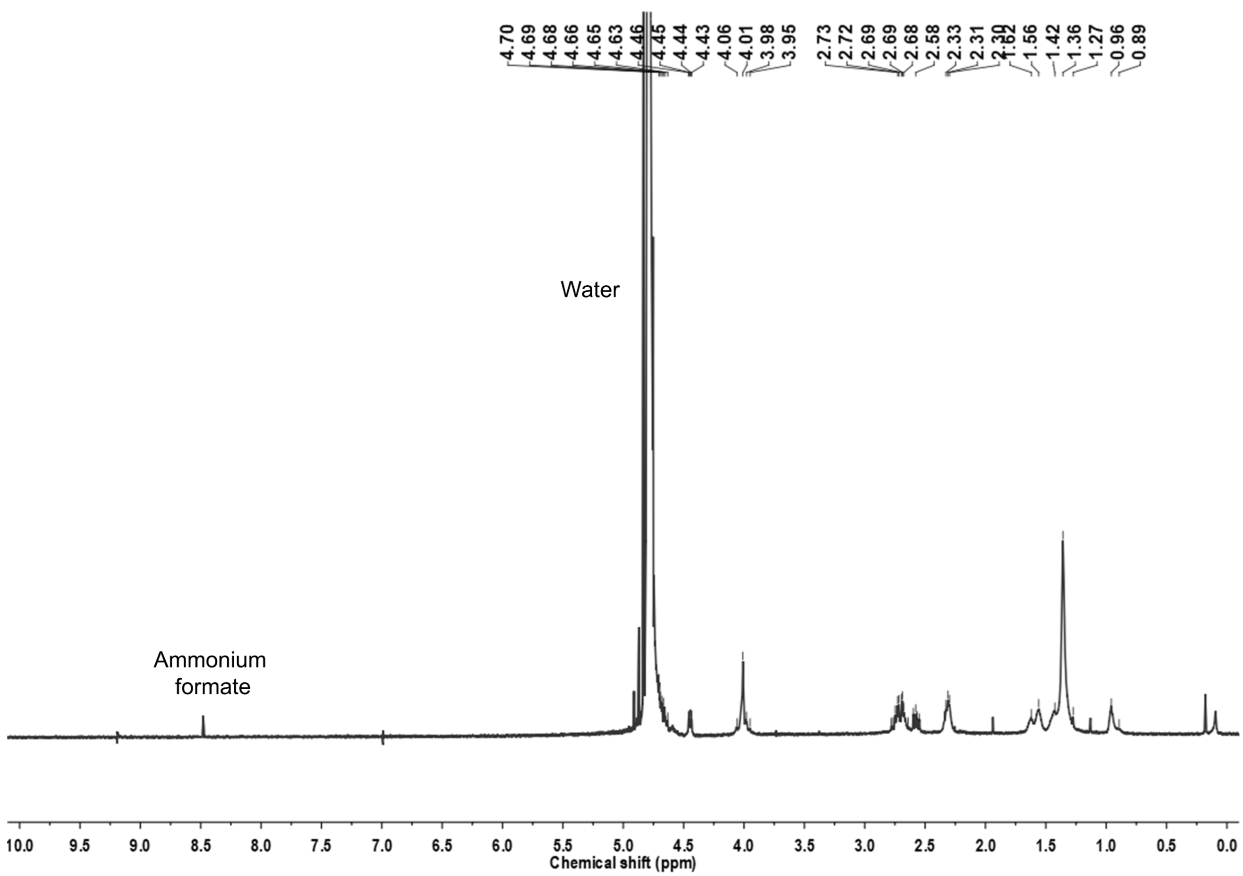

Figure S36. D<u>**GG**</u>DGD-PDA characterization by 1 H-NMR (500 MHz, D2O). δ, ppm: 4.43-4.46, 4.63-4.70 (m, Asp-αH, 3H), 3.95-4.06 (m, Gly-αH, 6H), 2.55-2.60, 2.64-2.78 (m, Asp-βH, 6H), 2.30-2.34 (m, PDA-H, 6H), 1.27-1.42, 1.56-1.62 (m, PDA-H, 32H), 0.89-0.96 (m, PDA-H, 3H).

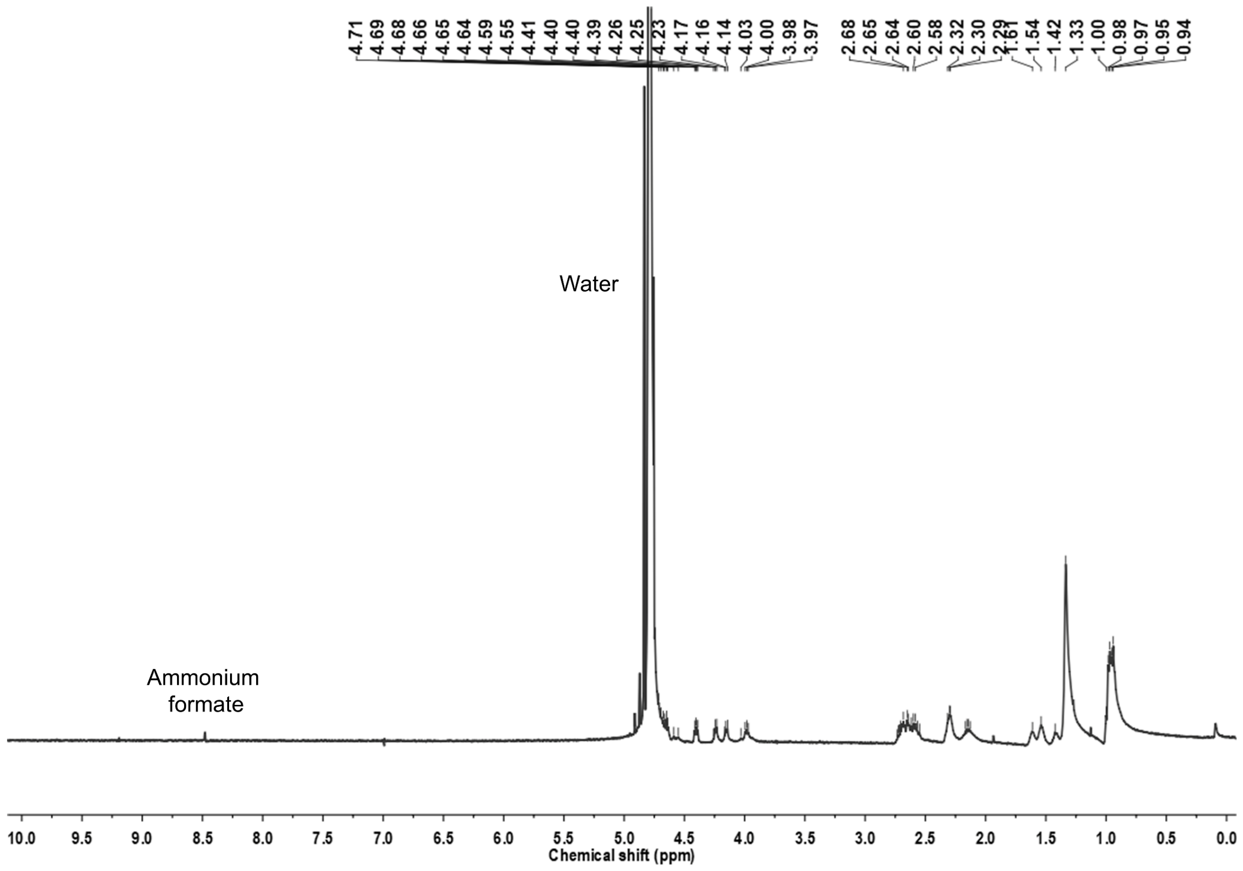

Figure S37. D<u>**VV**</u>DGD-PDA characterization by 1 H-NMR (500 MHz, D2O) δ, ppm: 4.39-4.41, 4.55-4.71 (m, Asp-αH, 3H), 4.14-4.17, 4.23-4.26 (m, Val-αH, 2H), 3.97-4.03 (m, Gly-αH, 2H), 2.55-2.74 (m, Asp-βH, 6H), 2.13-2.27, 2.29-2.32 (m, PDA-H, 6H), 1.33-1.61 (m, PDA-H, 32H), 0.94-1.00 (m, PDA-H, 3H; Val-γH, 12H).

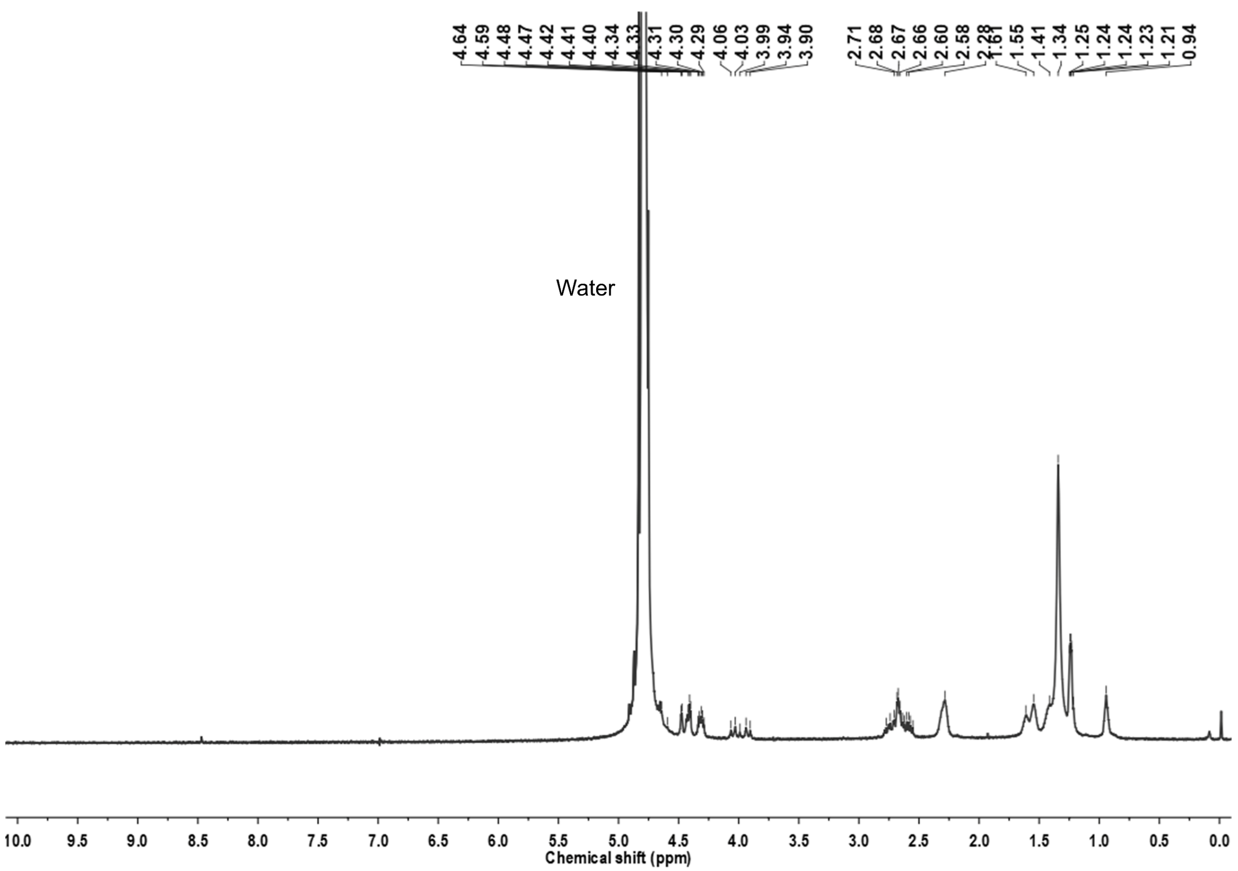

Figure S38. D**TT**DGD-PDA characterization by 1 H-NMR (500 MHz, D2O) δ, ppm: 4.47-4.48, 4.59-4.64 (m, Asp-αH, 3H), 4.29-4.34, 4.40-4.42 (m, Thr-αH, 2H), 3.90-4.06 (m, Gly-αH, 2H), 2.55-2.77 (m, Asp-βH, 6H), 2.28 (s, PDA-H, 6H), 1.34-1.61 (m, PDA-H, 32H), 1.21-1.25 (m, Thr-γH, 6H), 0.94 (s, PDA-H, 3H).

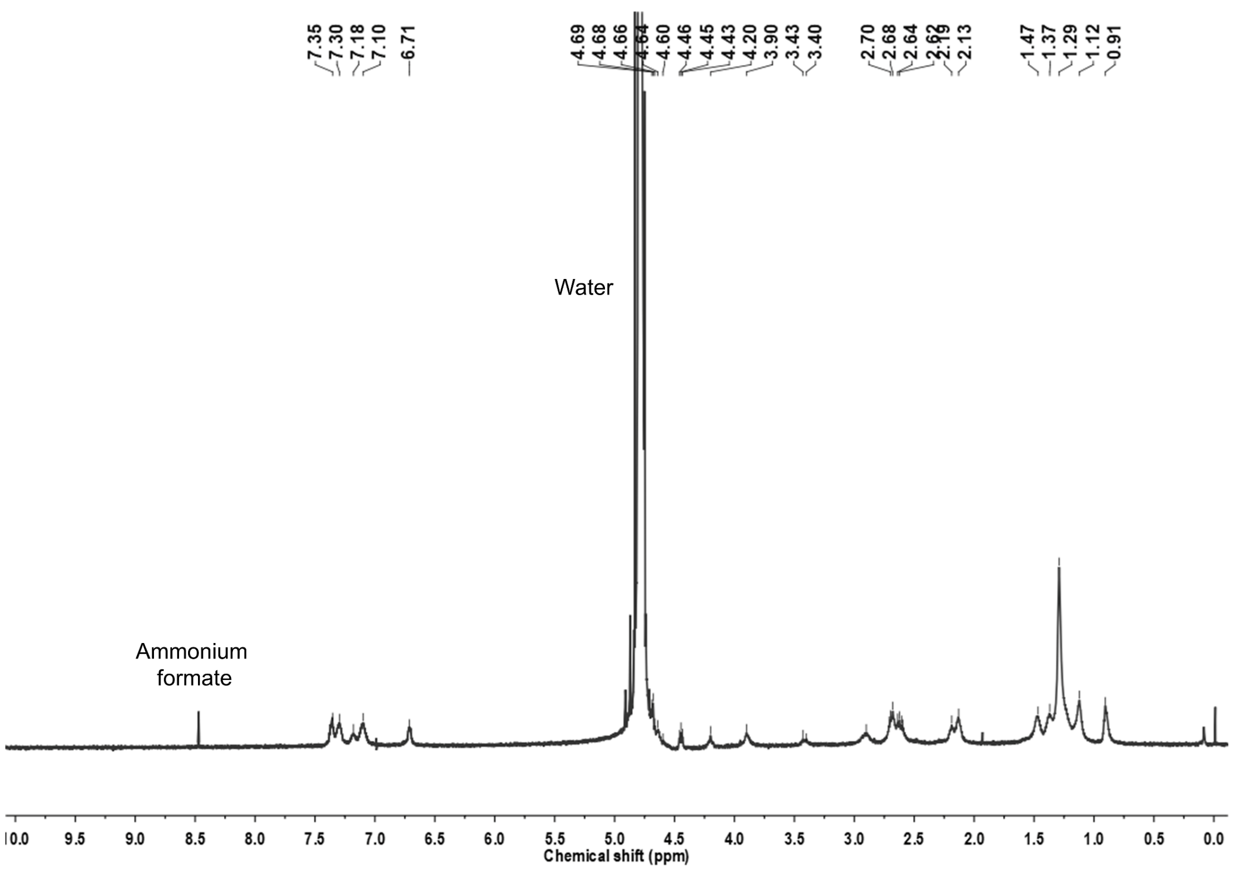

Figure S39. D<u>**FF**</u>DGD-PDA characterization by 1 H-NMR (500 MHz, D2O) δ, ppm: 7.30-7.35 (m, Phe-3, 5H, 4H), 7.10-7.18 (m, Phe-2, 6H, 4H), 6.71 (m, Phe-4H, 2H), 4.60-4.69(m, Asp-αH, 3H), 4.20, 4.43-4.46 (m, Phe-αH, 2H), 3.90 (s, Gly-αH, 2H), 3.40-3.43 (m, Phe-βH, 1H,) 2.90 (s, Phe-βH, 2H,) 2.59-2.71 (m, Phe-βH, 1H, Asp-βH, 6H), 2.13-2.19 (m, PDA-H, 6H), 1.12-1.47 (m, PDA-H, 32H), 0.91 (s, PDA-H, 3H).

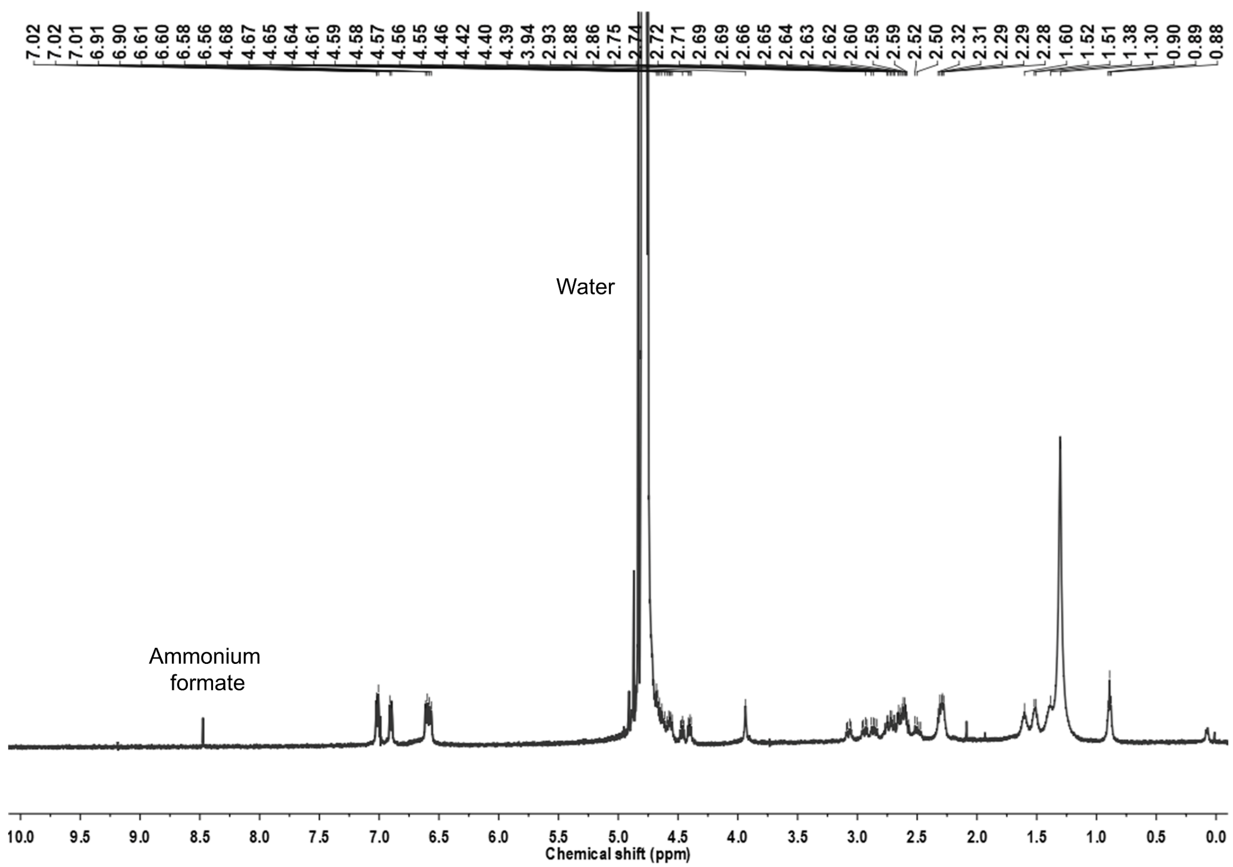

Figure S40. D<u>**YY**</u>DGD-PDA characterization by 1 H-NMR (500 MHz, D2O) δ, ppm: 6.90-6.91, 7.01-7.02 (m, Tyr-2,6H, 4H), 6.56-6.91 (m, Tyr-3,5H, 4H), 4.55-4.68 (m, Asp-αH, 3H), 4.39-4.42, 4.44-4.48 (m, Tyr-αH, 2H), 3.94 (s, Gly-αH, 2H), 2.84-2.88, 2.92-2.96, 3.05-3.09 (m, Tyr-βH, 4H), 2.47-2.77 (m, Asp-βH, 6H), 2.28-2.32 (m, PDA-H, 6H), 1.30-1.60 (m, PDA-H, 32H), 0.88-0.90 (m, PDA-H, 3H).

Characterization of synthesized peptide-PDA polymers with SEC

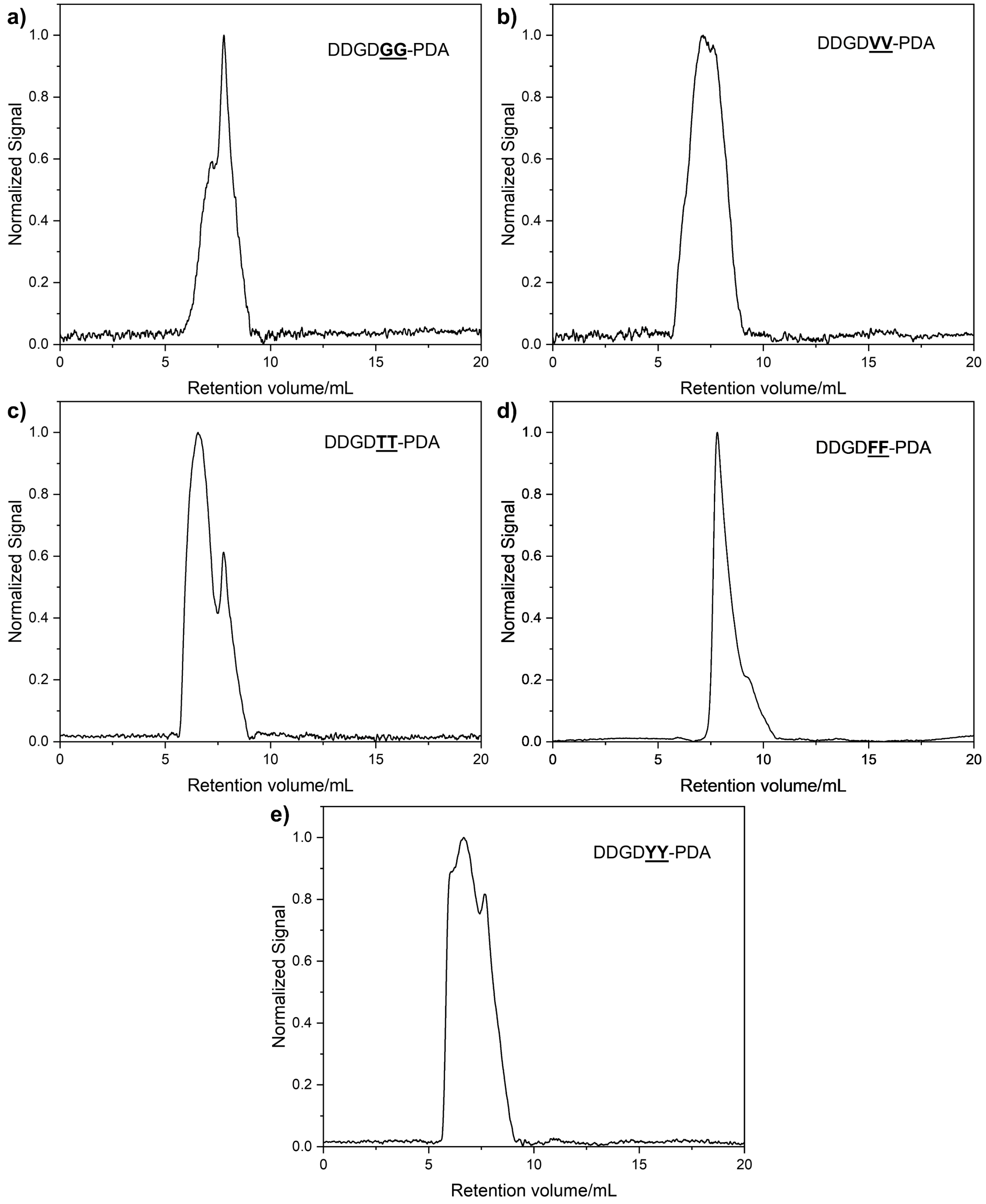

Figure S41. SEC curve of 1 mg/mL (a) DDGD**GG**-PDA, (b) DDGD**VV**-PDA, (c) DDGD**TT**-PDA, (d) DDGD**FF**-PDA, and (e) DDGD**YY**-PDA dissolved in 0.1% ammonium formate buffer.

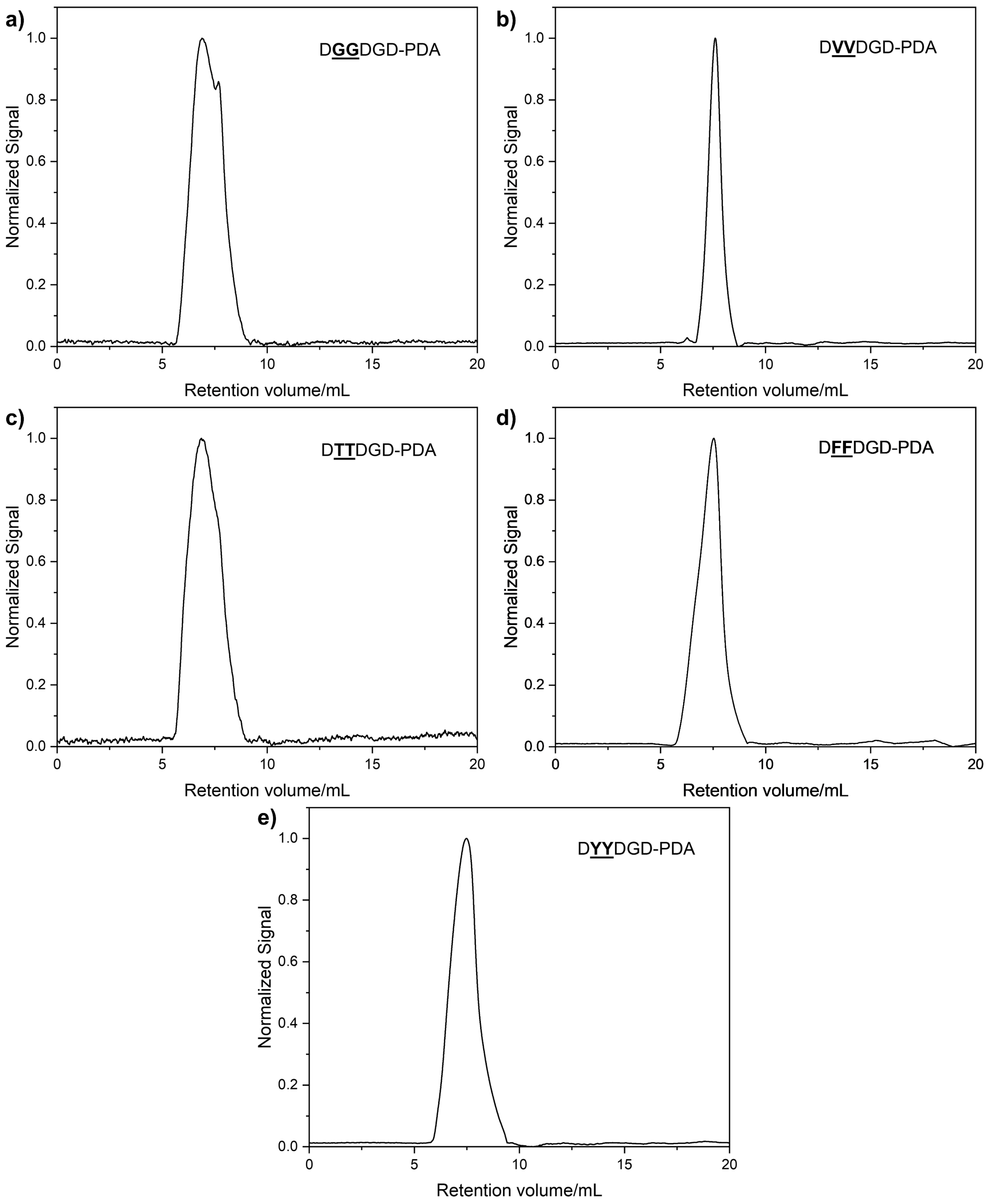

Figure S42. SEC curve of 1 mg/mL (a) D**GG**DGD-PDA, (b) D**VV**DGD-PDA, and (c) D**TT**DGD-PDA (d) D**FF**DGD-PDA, and (e) D**YY**DGD-PDA dissolved in 0.1% ammonium formate buffer.

Table S1. Summary of M_n, M_w and PDI of peptide-PDA samples.

Sample	M_n/Dalton	M_w/Dalton	PDI
DDGD**GG**-PDA	1.44×10^6	1.16×10^7	8.04
DDGD**VV**-PDA	2.95×10^6	2.81×10^7	9.53
DDGD**TT**-PDA	4.72×10^6	5.53×10^7	11.72
DDGD**FF**-PDA	2.01×10^5	1.36×10^6	6.77
DDGD**YY**-PDA	3.16×10^6	5.83×10^7	18.45
D**GG**DGD-PDA	3.83×10^6	3.19×10^7	8.32
D**VV**DGD-PDA	3.10×10^6	5.42×10^6	1.75
D**TT**DGD-PDA	4.42×10^6	4.52×10^7	10.21
D**FF**DGD-PDA	3.49×10^6	1.95×10^7	5.59
D**YY**DGD-PDA	1.96×10^6	1.64×10^7	8.37

Calculation of molecular volume and molecular polarity index (MPI)

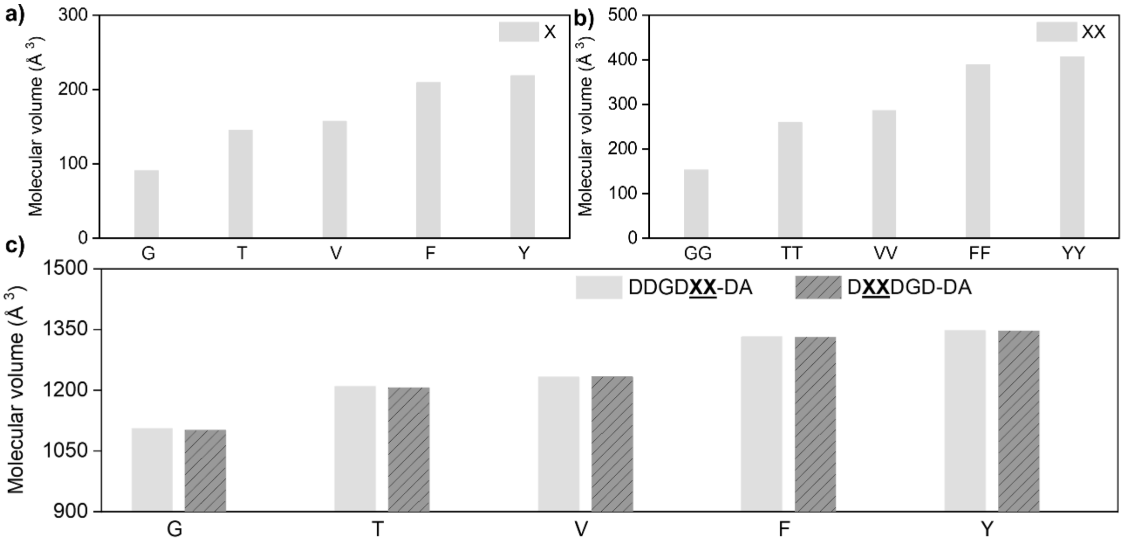

Figure S43. Molecular volume of the involved (a) single amino acid, (b) XX dipeptide, and (c) DDGD**XX**-DA and D**XX**DGD-DA (X = G, V, T, F, and Y).

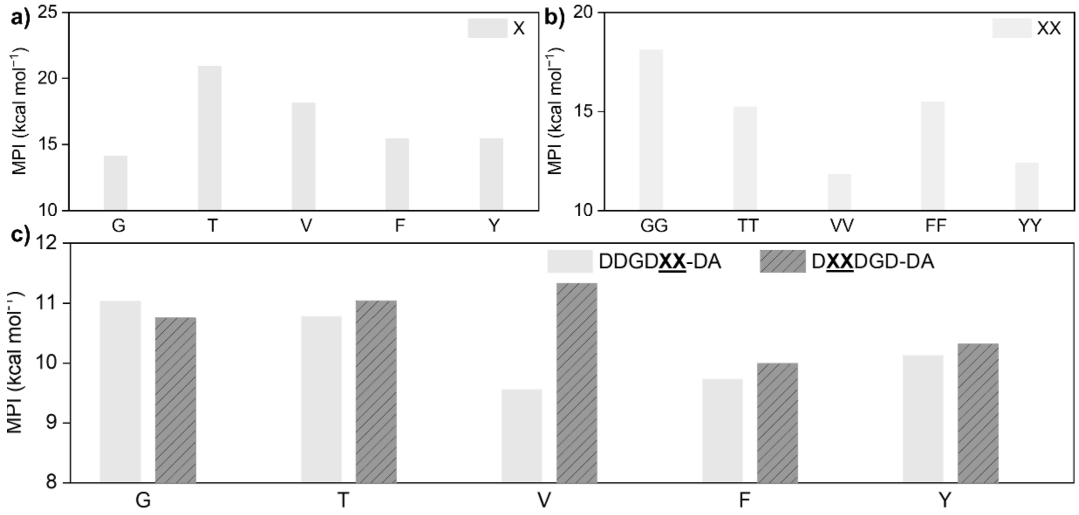

Figure S44. MPI of the involved (a) single amino acid, (b) XX dipeptide, and (c) DDGD**XX**-DA and D**XX**DGD-DA (X = G, V, T, F, and Y)

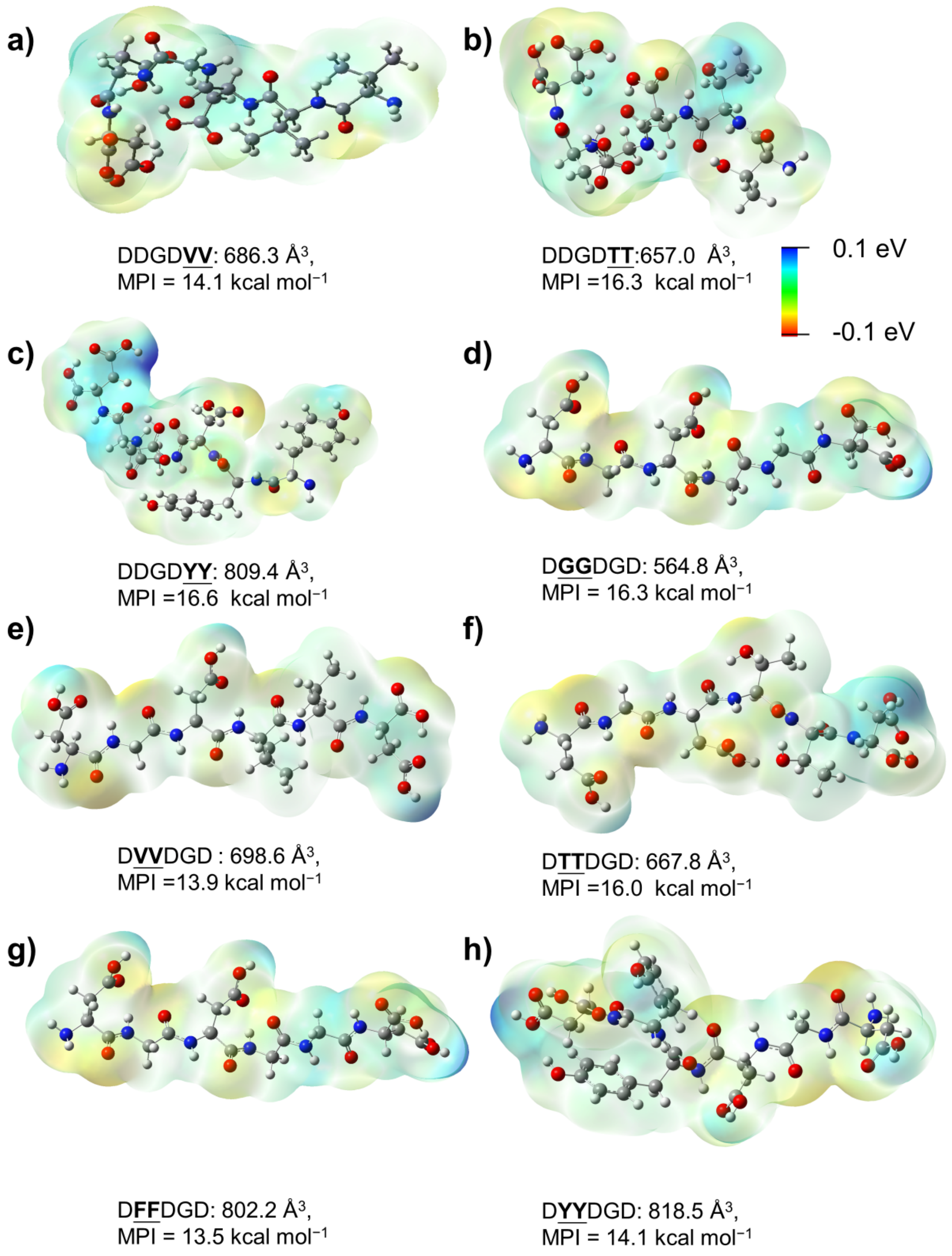

Figure S45. Electrostatic potential surface mapping of (a) DDGD**VV**, (b) DDGD**TT**, (c) DDGD**YY**, (d) D**GG**DGD, (e) D**VV**DGD, (f) D**TT**DGD, (g) D**FF**DGD, and (h) D**YY**DGD.

Spectral characterization and TEM images

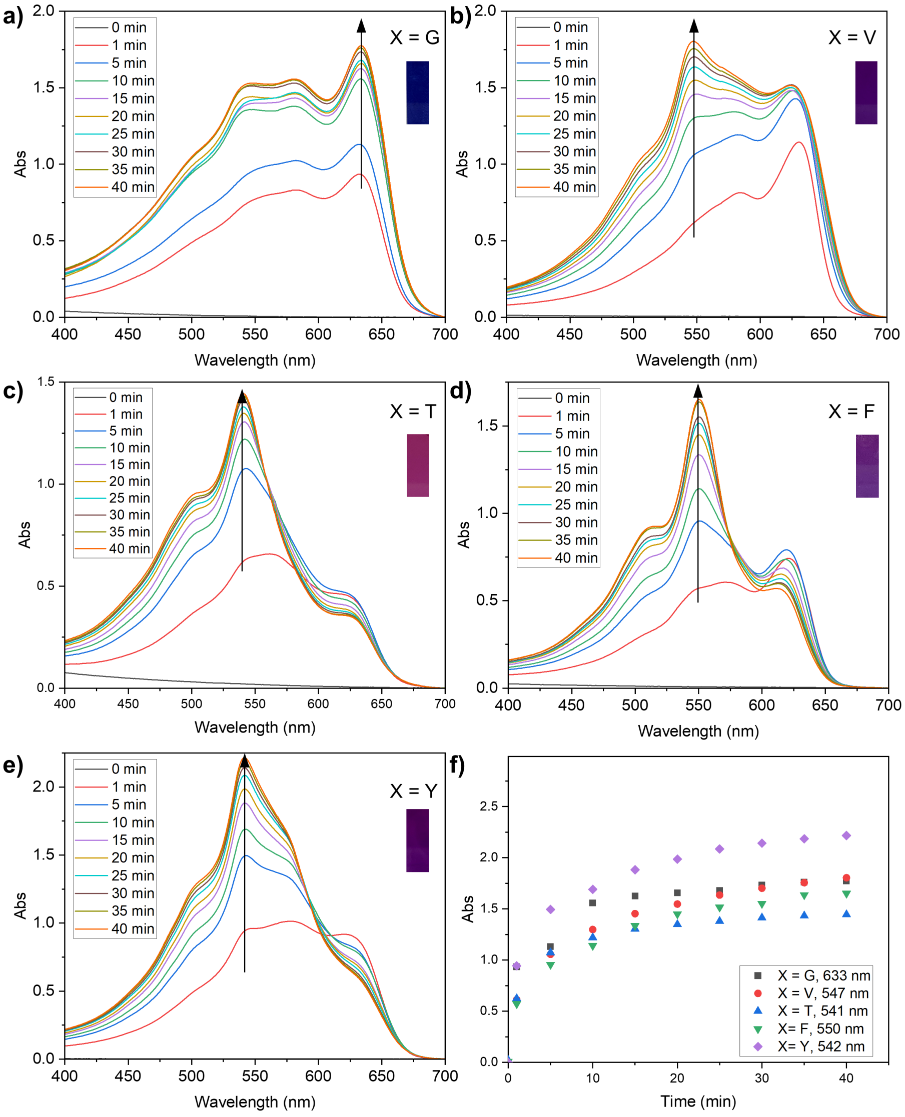

Figure S46. Absorption peak evolution for DDGD**XX**-PDA at different polymerization times. DDGD**XX**-PDA (X = G, V, T, F, and Y) polymerized with 254 nm UV-irradiation from 0 to 40 min: (a) DDGD**GG**-PDA, (b) DDGD**VV**-PDA, (c) DDGD**TT**-PDA, (d) DDGD**FF**-PDA, and (e) DDGD**YY**-PDA. Arrows represent increasing irradiation time. (f) Absorption evolution of the final λ_{max} for each sample at different irradiation times.

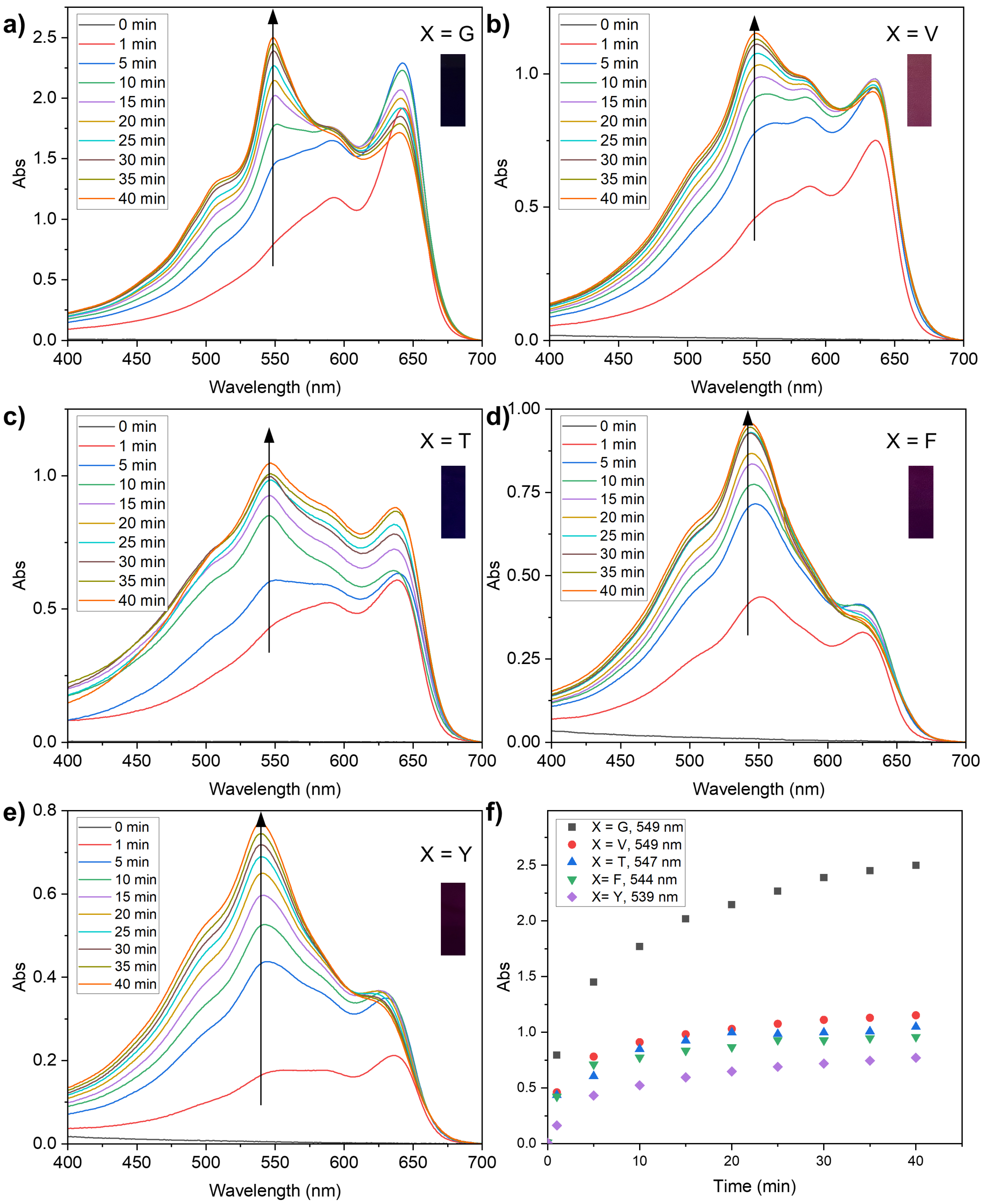

Figure S47. Absorption peak evolution for D**XX**DGD-PDA at different polymerization times. D**XX**DGD-PDA (X=G, V, T, F, and Y) polymerized with 254-nm UV-irradiation from 0 to 40 min: (a) D**GG**DGD-PDA, (b) D**VV**DGD-PDA, (c) D**TT**DGD-PDA, (d) D**FF**DGD-PDA, and (e) D**YY**DGD-PDA). Arrows represent increasing irradiation time. (f) Absorption evolution of the final λ_{max} for each sample at different irradiation times.

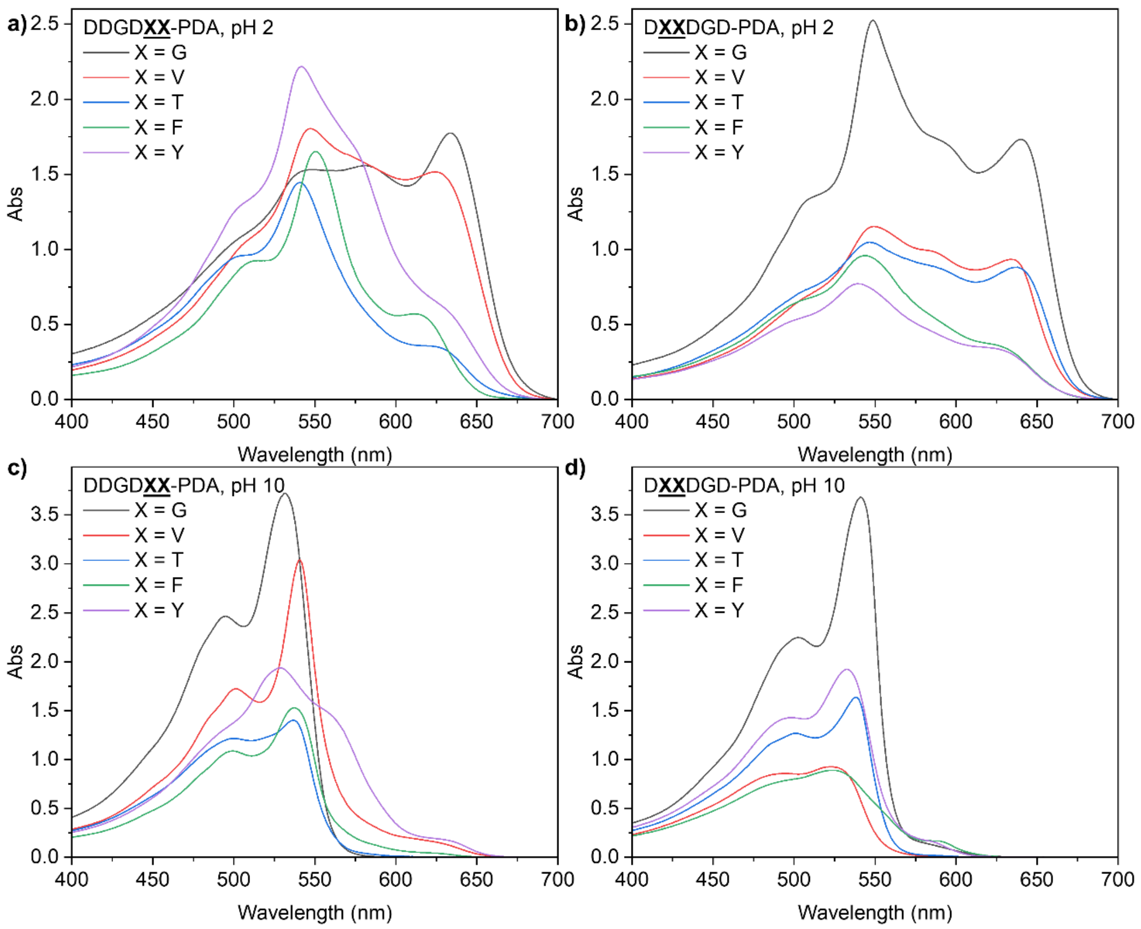

Figure S48. UV-vis spectra of peptide-PDA with 254 nm UV irradiation for 40 min at different pH conditions. (a) DDGD**XX**-PDA and (b) D**XX**DGD-PDA (X = G, V, T, F, and Y) polymerized with 254 nm UV irradiation at pH 2 for 40 min. UV-vis spectra of (c) DDGD**XX**-PDA and (d) D**XX**DGD-PDA (X = G, V, T, F, and Y) polymer solution at pH 10.

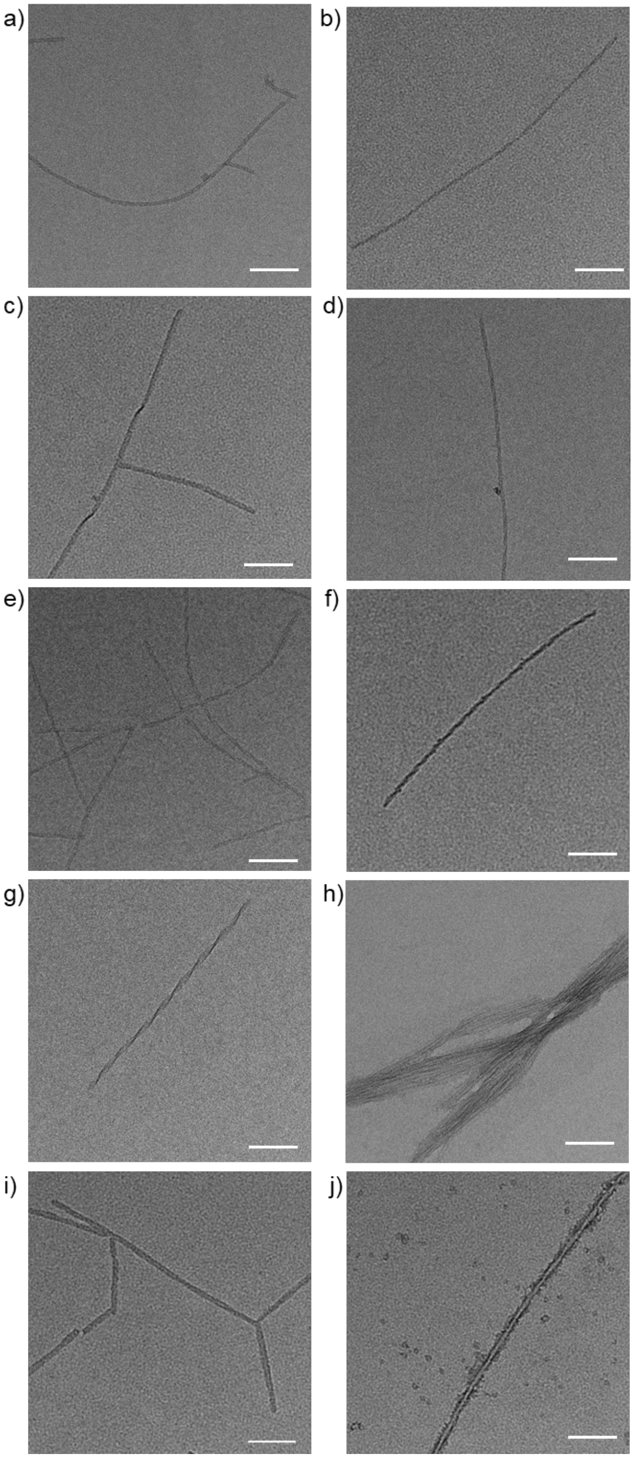

Figure S49. TEM images of (a)-(e) DDGD**XX**-DA assemblies at pH 2 and (f)-(j) DDGD**XX**-PDA polymer at pH 10 (X = G, V, T, F, and Y). Scale bar = 100 nm.

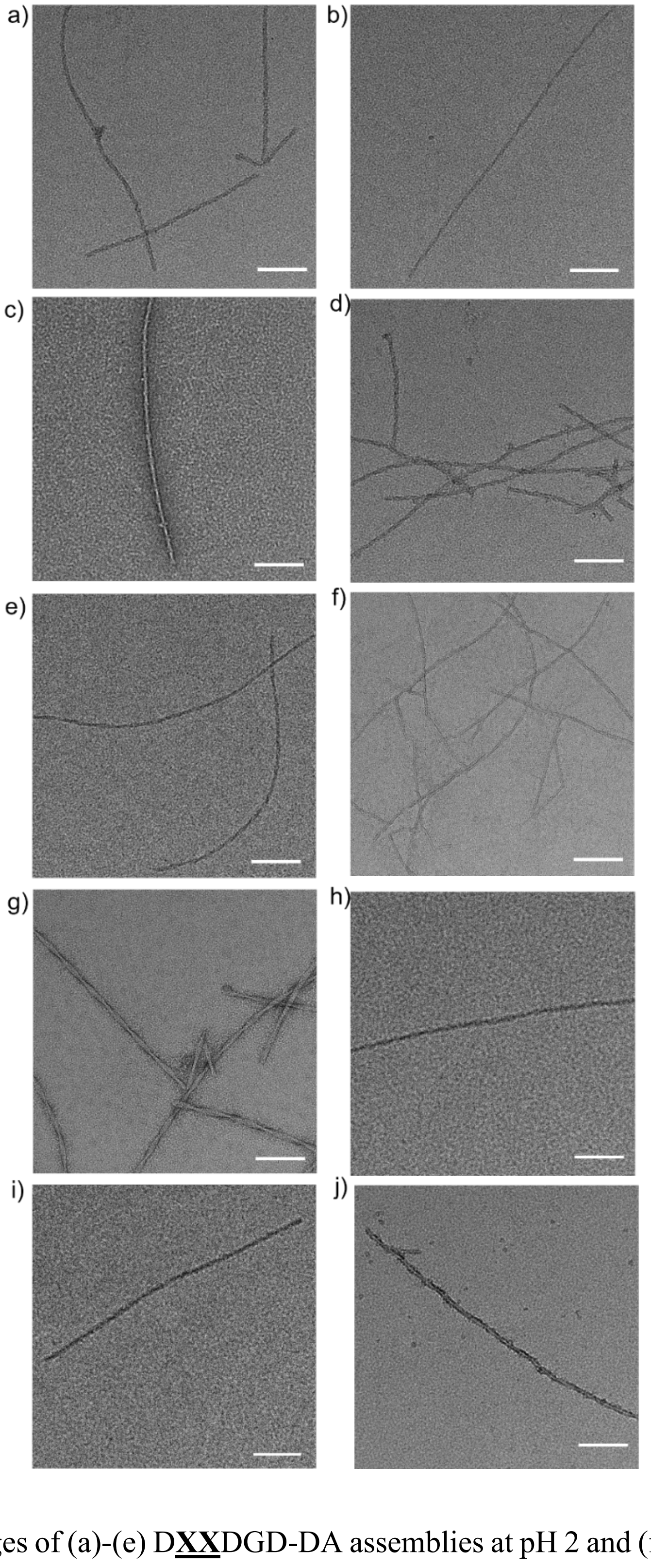

Figure S50. TEM images of (a)-(e) D**XX**DGD-DA assemblies at pH 2 and (f)-(j) D**XX**DGD-PDA polymer at pH 10 (X = G, V, T, F, and Y). Scale bar = 100 nm.

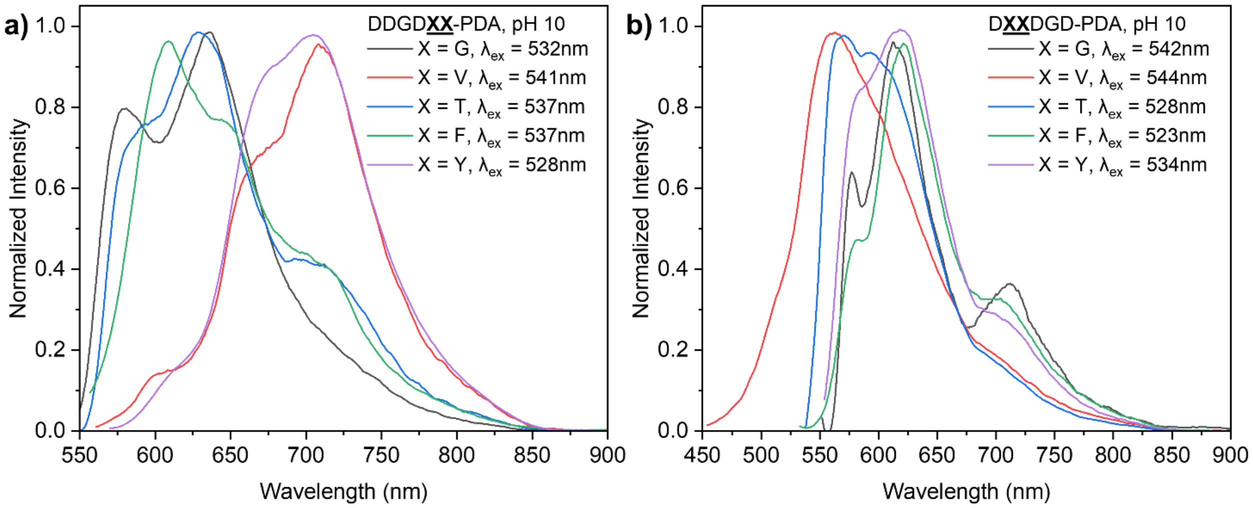

Figure S51. Photoluminescence spectra of (a) DDGD**XX**-PDA and (b) D**XX**DGD-PDA (X = G, V, T, F, and Y) polymerized with 40 min 254-nm UV-irradiation at pH 2, then switched to pH 10.

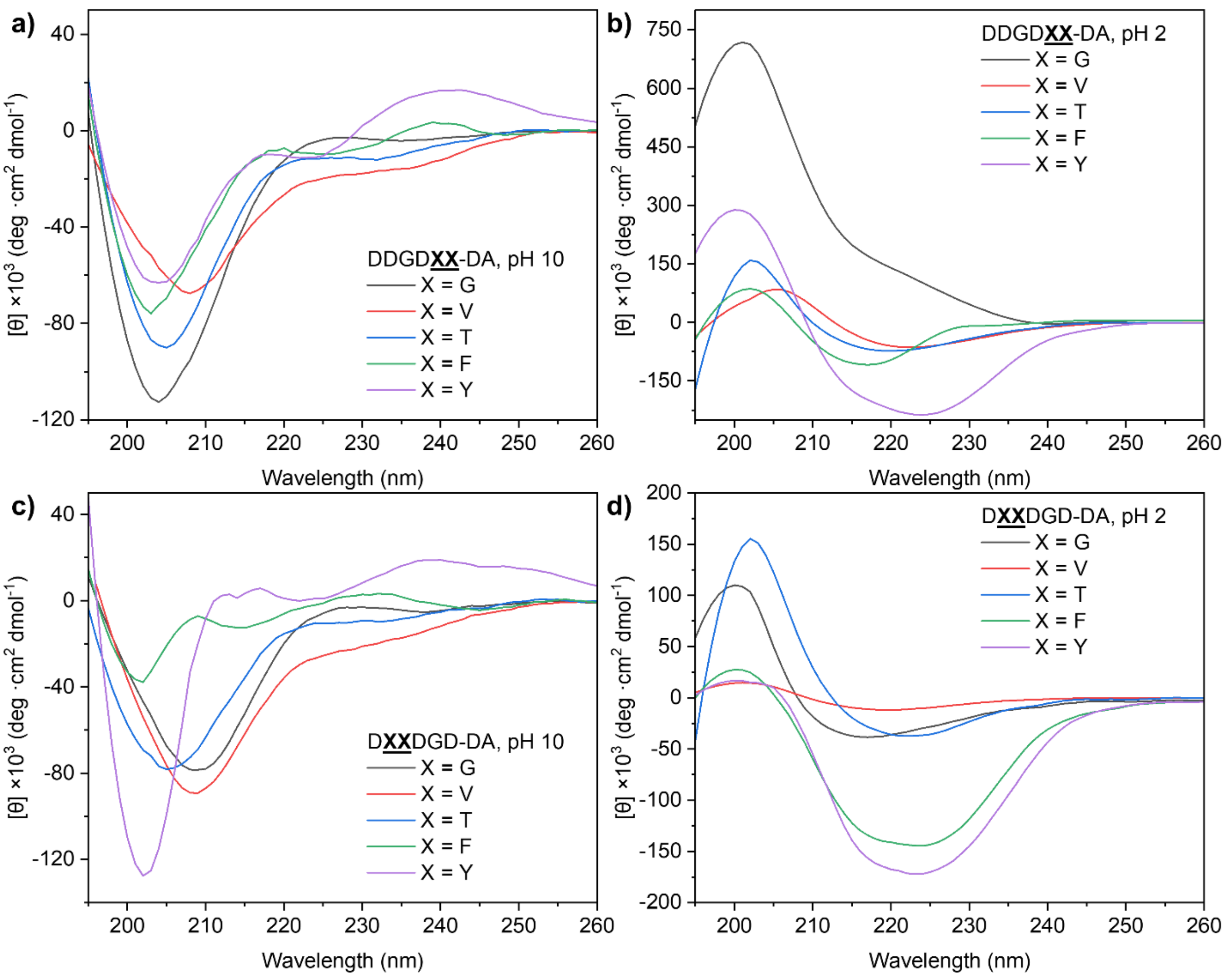

Figure S52. Monitoring the secondary structure of monomeric peptide-DA self-assemblies under different pH conditions. CD spectra of (a) DDGD**XX**-DA and (c) D**XX**DGD-DA (X = G, V, T, F, and Y) self-assemblies at pH 10. CD spectra of (b) DDGD**XX**-DA and (d) D**XX**DGD-DA (X = G, V, T, F, and Y) self-assemblies at pH 2.

Molecular simulation

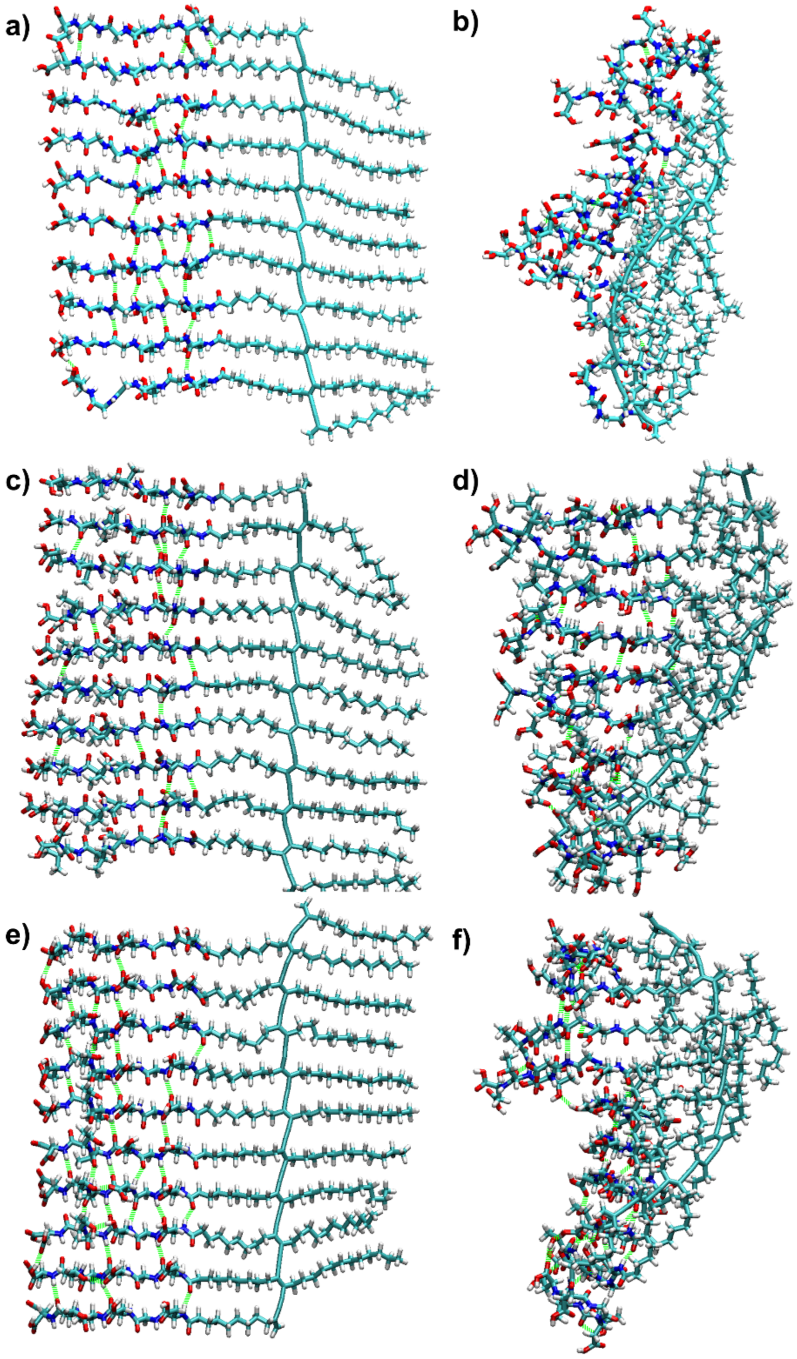

Figure S53. Molecular structures after energy relaxation of peptide-PDA (decamer). D**GG**DGD-PDA simulated at (a) 0 ns and (b) 10 ns, D**VV**DGD-PDA simulated at (c) 0 ns and (d) 10ns, and D**TT**DGD-PDA simulated at (e) 0 ns and (f) 10 ns.

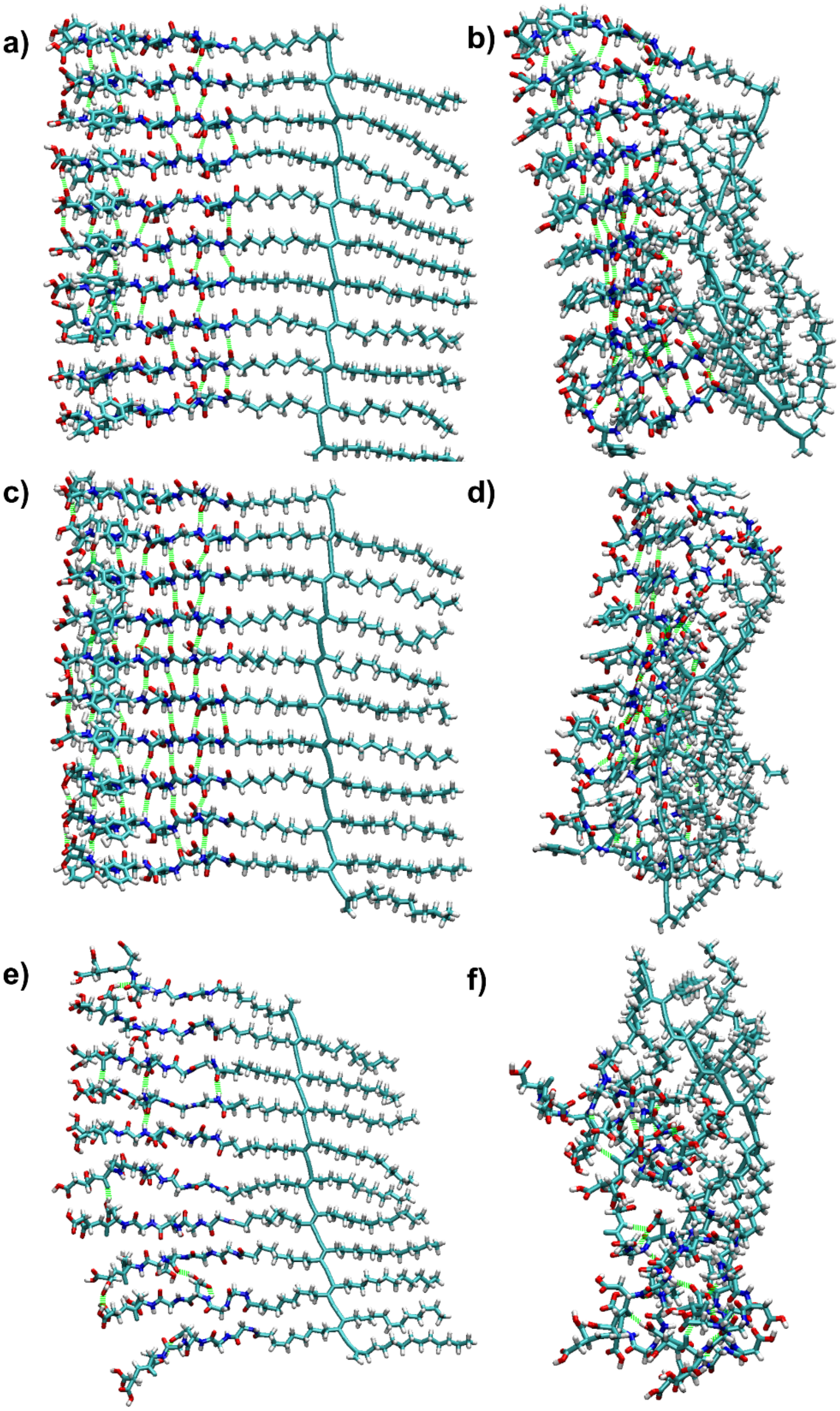

Figure S54. Molecular structures after energy relaxation of peptide-PDA (decamer). D**FF**DGD-PDA simulated at (a) 0 ns and (b) 10 ns, D**YY**DGD-PDA simulated at (c) 0 ns and (d) 10 ns, and DDGD**GG**-PDA simulated at (e) 0 ns and (f) 10 ns.

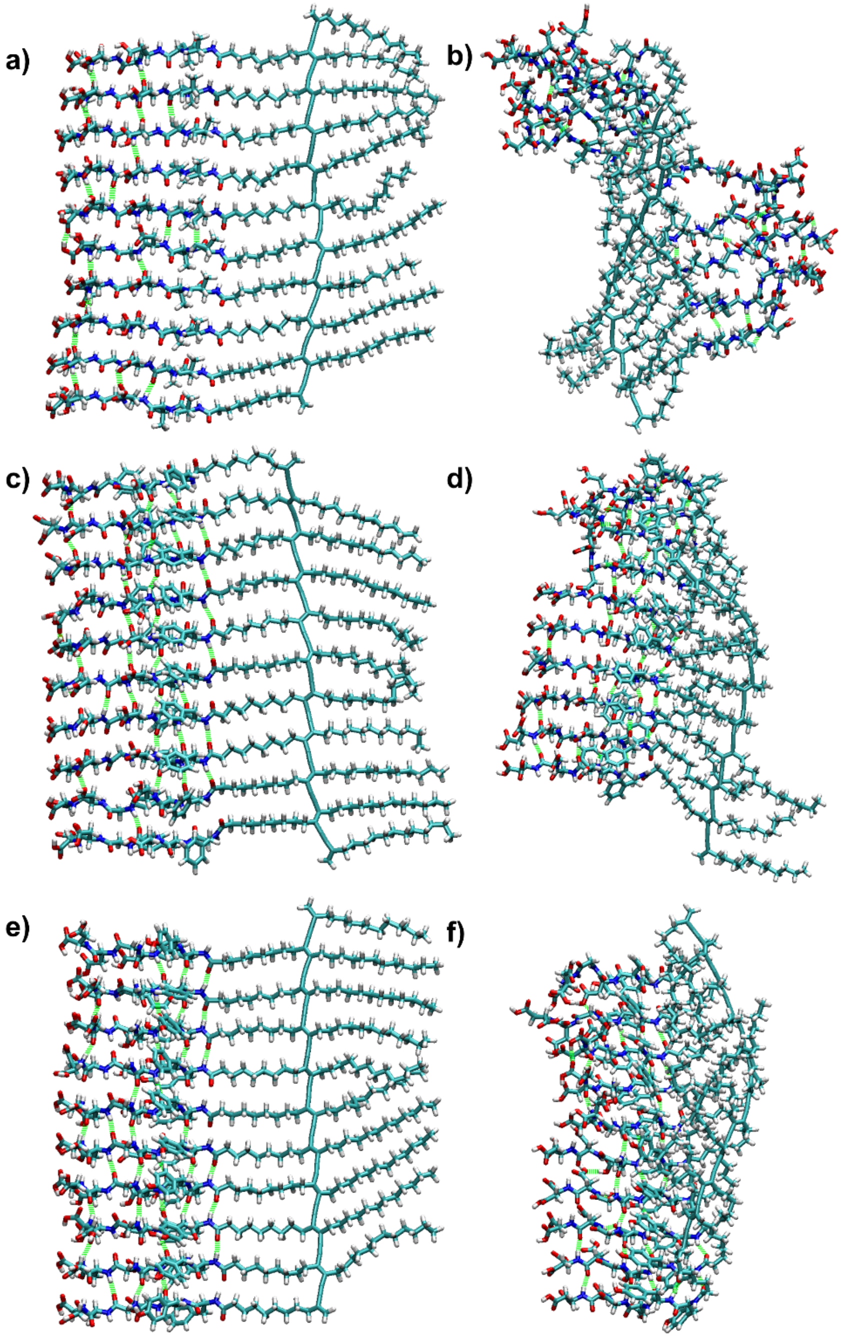

Figure S55. Molecular structures after energy relaxation of peptide-PDA (decamer). DDGD**TT**-PDA simulated at (a) 0 ns and (b) 10 ns, DDGD**FF**-PDA simulated at (c) 0 ns and (d) 10 ns, and DDGD**YY**-PDA simulated at (e) 0 ns and (f) 10 ns.

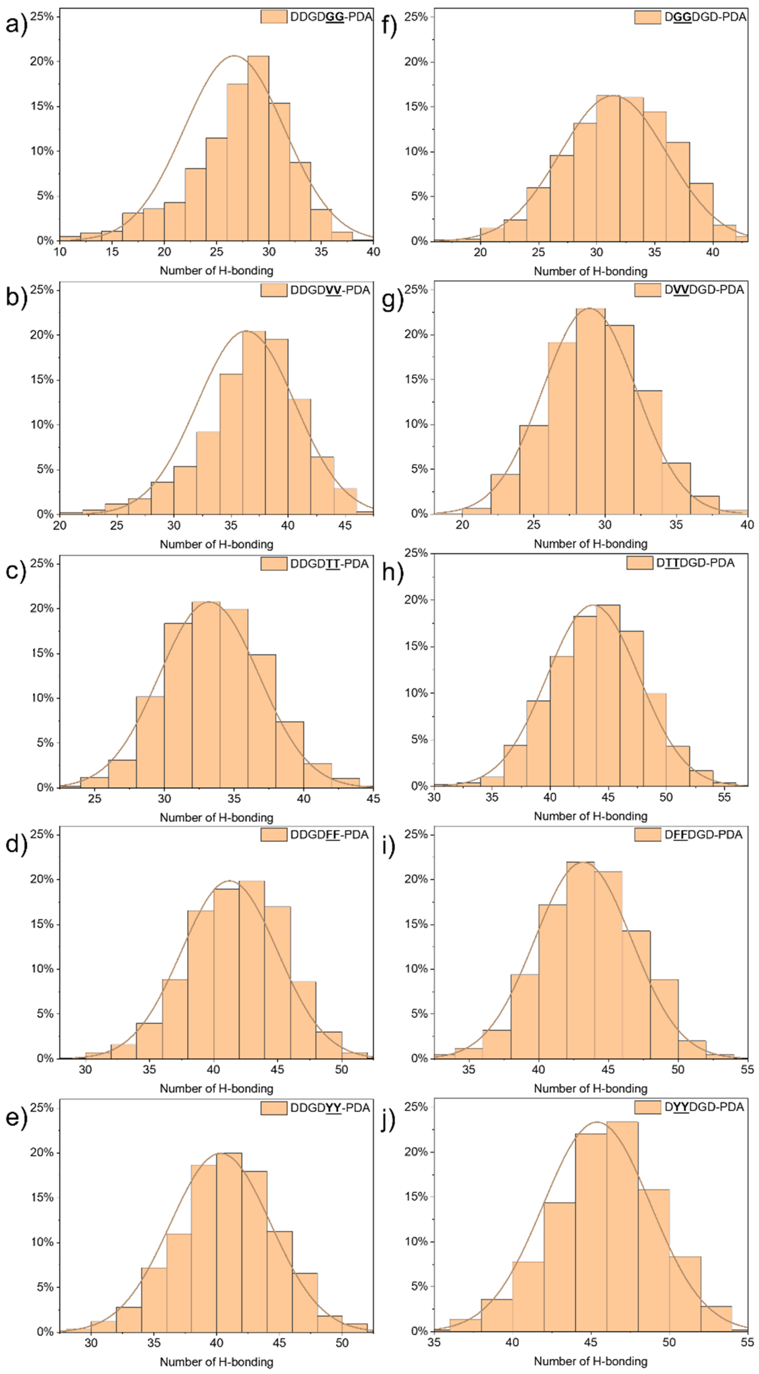

Figure S56. Distribution of intramolecular H-bonding between peptide moieties within 10 ns after energy relaxation for peptide-PDA (decamer). (a) DDGD**GG**-PDA, (b) DDGD**VV**-PDA, (c) DDGD**TT**-PDA, (d) DDGD**FF**-PDA, (e) DDGD**YY**-PDA, (f) D**GG**DGD-PDA, (g) D**VV**DGD-PDA, (h) D**TT**DGD-PDA, (i) D**FF**DGD-PDA, and (j) D**YY**DGD-PDA.

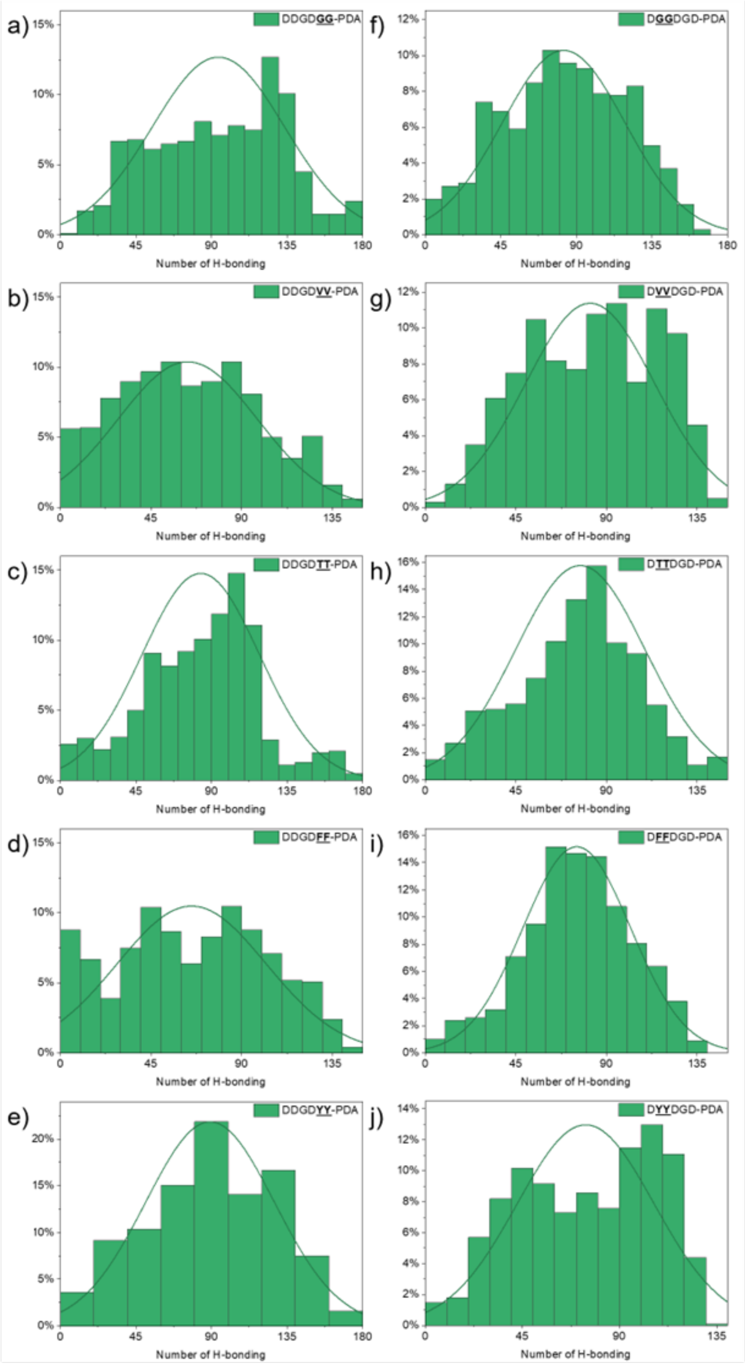

Figure S57. Distribution of intermolecular H-bonding between peptide moieties and water within 10ns after energy relaxation for peptide-PDA (decamer). (a) DDGD**GG**-PDA, (b) DDGD**VV**-PDA, (c) DDGD**TT**-PDA, (d) DDGD**FF**-PDA, (e) DDGD**YY**-PDA, (f) D**GG**DGD-PDA, (g) D**VV**DGD-PDA, (h) D**TT**DGD-PDA, (i) D**FF**DGD-PDA, and (j) D**YY**DGD-PDA.

Table S2. Calculated *p*-values from the *t*-tests performed on Figure 6d data set.

Comparison	*p*-value	Comparison	*p*-value
DDGD**GG** vs D**GG**DGD	$p < 0.0001$	DDGD**VV** vs D**VV**DGD	$p < 0.0001$
DDGD**TT** vs D**TT**DGD	$p < 0.0001$	DDGD**FF** vs D**FF**DGD	$p < 0.0001$
DDGD**YY** vs D**YY**DGD	$p < 0.0001$	DDGD**GG** vs DDGD**VV**	$p < 0.0001$
DDGD**GG** vs DDGD**TT**	$p < 0.0001$	DDGD**GG** vs DDGD**FF**	$p < 0.0001$
DDGD**GG** vs DDGD**YY**	$p < 0.0001$	DDGD**VV** vs DDGD**TT**	$p < 0.0001$
DDGD**VV** vs DDGD**FF**	n.s.	DDGD**VV** vs DDGD**YY**	n.s.
DDGD**TT** vs DDGD**FF**	$p < 0.0001$	DDGD**TT** vs DDGD**YY**	$p < 0.0001$
DDGD**FF** vs DDGD**YY**	n.s.	D**GG**DGD vs D**VV**DGD	$p < 0.0001$
D**GG**DGD vs D**TT**DGD	n.s.	D**GG**DGD vs D**FF**DGD	$p < 0.0001$
D**GG**DGD vs D**YY**DGD	$p < 0.05$	D**VV**DGD vs D**TT**DGD	$p < 0.0001$
D**VV**DGD vs D**FF**DGD	$p < 0.0001$	D**VV**DGD vs D**YY**DGD	$p < 0.0001$
D**TT**DGD vs D**FF**DGD	$p < 0.0001$	D**TT**DGD vs D**YY**DGD	n.s.
D**FF**DGD vs D**YY**DGD	$p < 0.0001$		

*p< 0.05; **p< 0.01; ***p< 0.001; ****p< 0.0001; (not significant; p>0.05).

AFM characterization

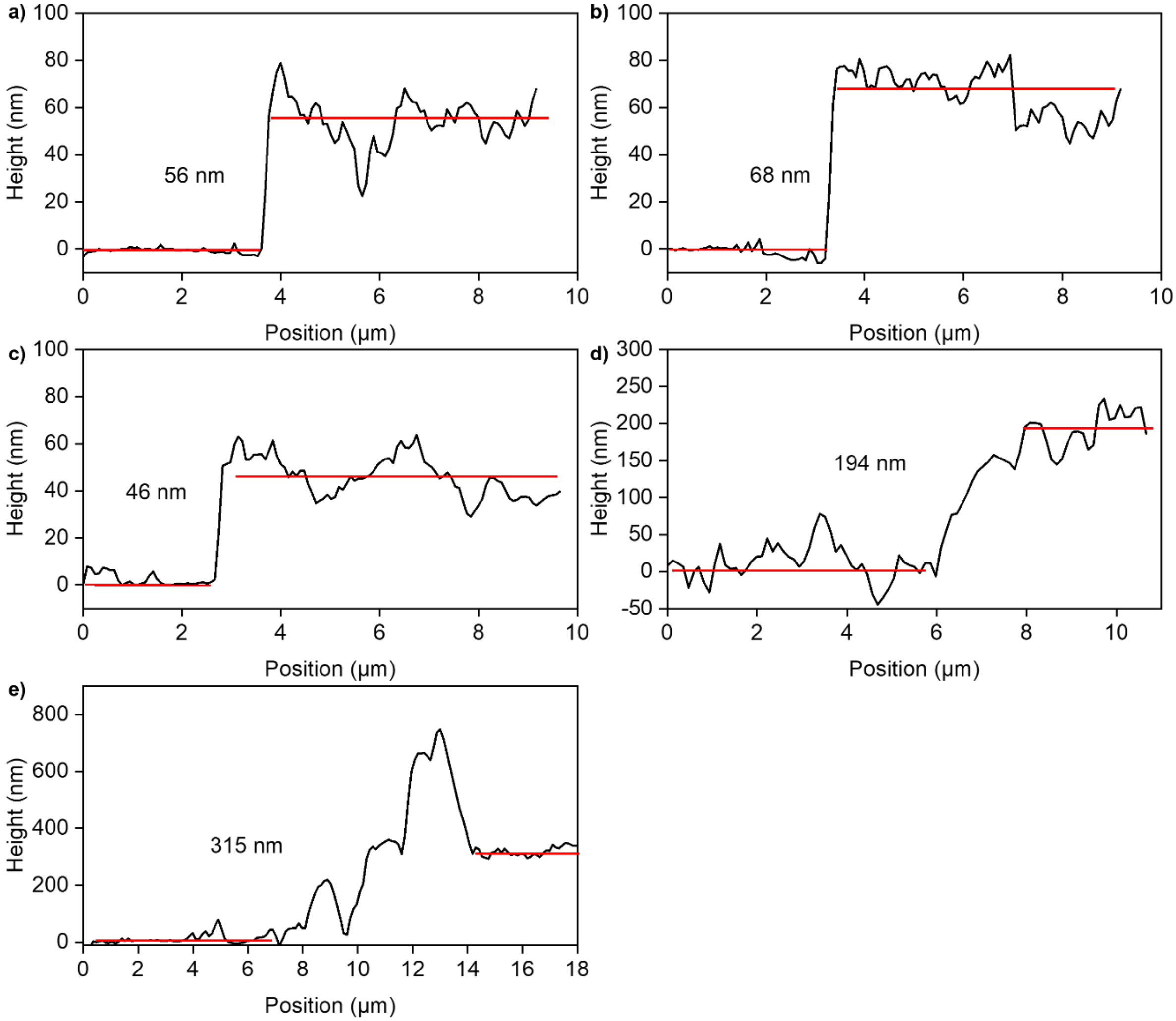

Figure S58. AFM thickness measurements of thin films made from 1 mM (a) DDGD**GG**-PDA, (b) DDGD**VV**-PDA, (c) DDGD**TT**-PDA, (d) DDGD**FF**-PDA, and (e) DDGD**YY**-PDA pH 2 solution. These films were used for the conductivity measurements.

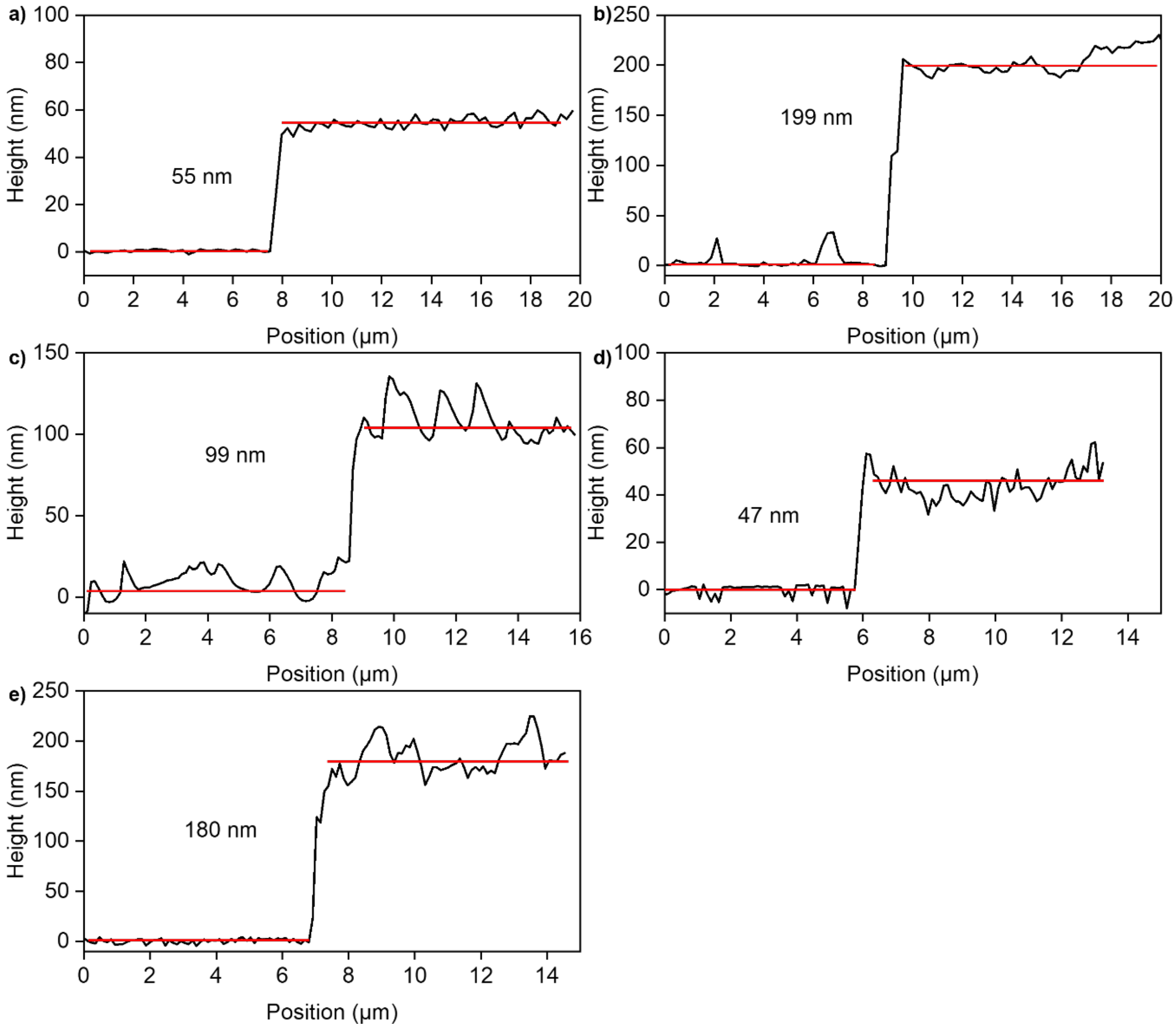

Figure S59. Thickness measurements of thin films made from 1 mM (a) DDGD**<u>GG</u>**-PDA, (b) DDGD**<u>VV</u>**-PDA, (c) DDGD**<u>TT</u>**-PDA, (d) DDGD**<u>FF</u>**-PDA, and (e) DDGD**<u>YY</u>**-PDA pH 10 solution by AFM. These films were used for the conductivity measurements.

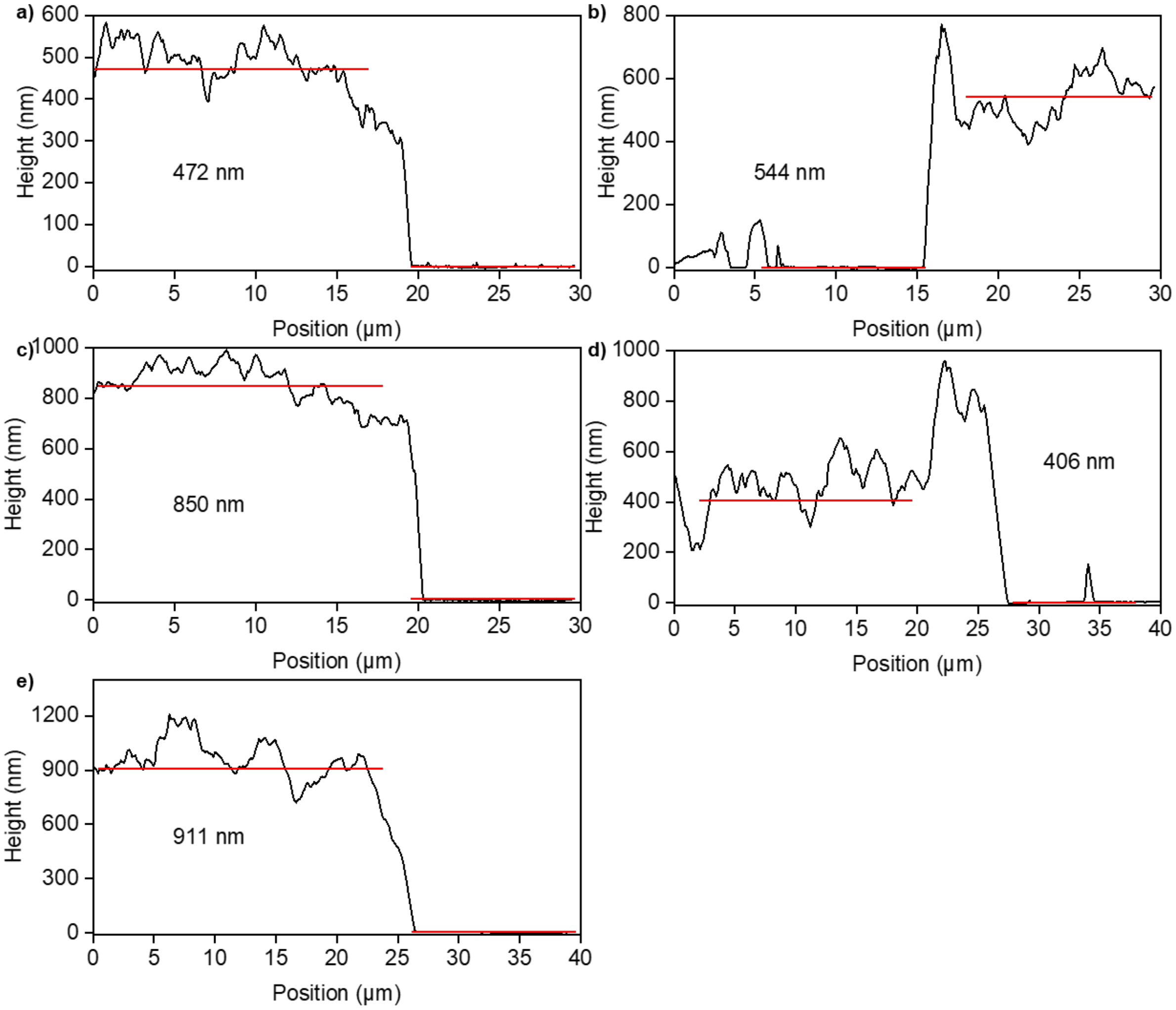

Figure S60. Thickness measurements of thin films made from 1 mM (a) D**GG**DGD-PDA, (b) D**VV**DGD-PDA, (c) D**TT**DGD-PDA, (d) D**FF**DGD-PDA, and (e) D**YY**DGD-PDA pH 2 solution by AFM. These films were used for the conductivity measurements.

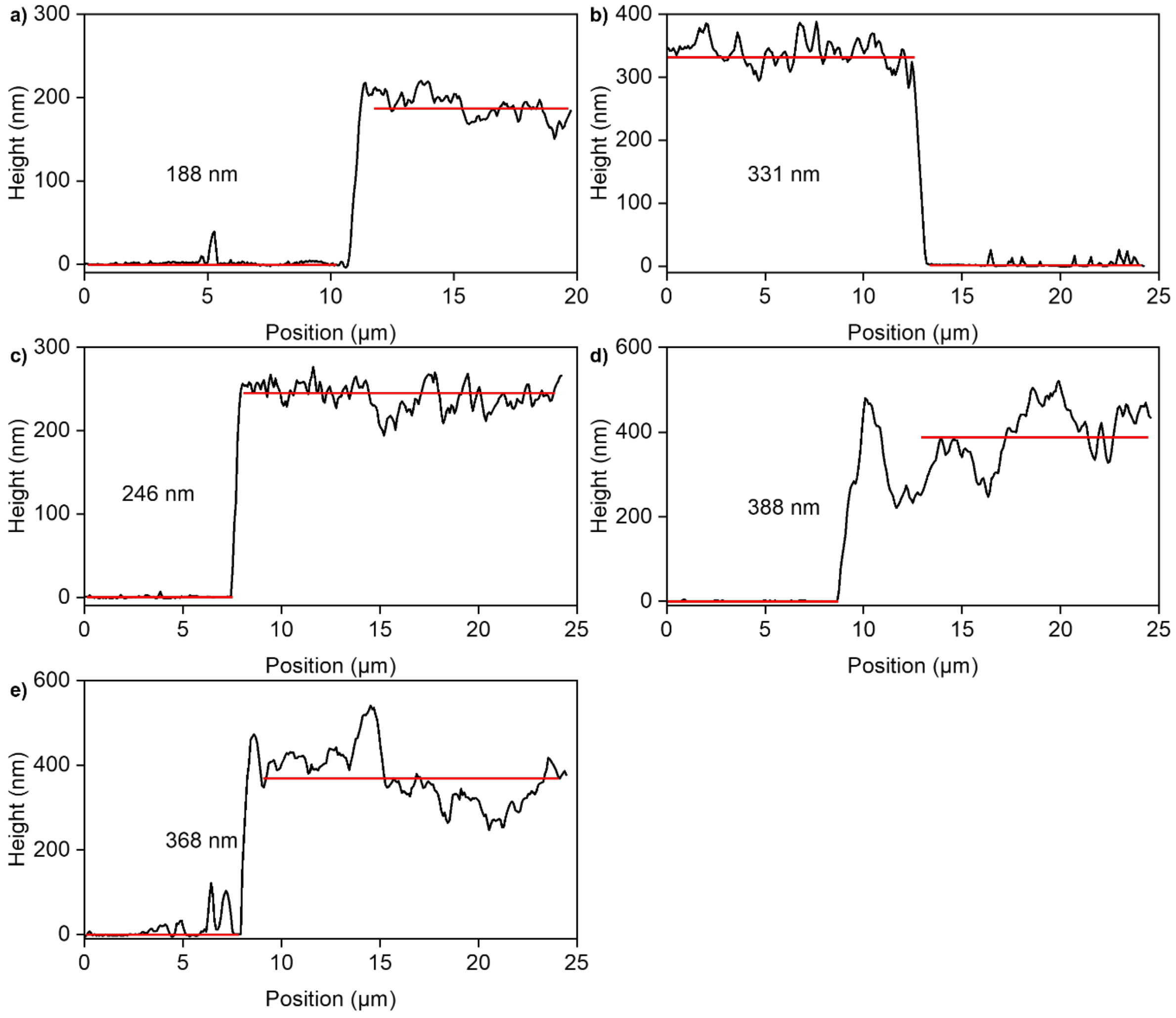

Figure S61. Thickness measurements of thin films made from 1 mM (a) D**GG**DGD-PDA, (b) D**VV**DGD-PDA, (c) D**TT**DGD-PDA, (d) D**FF**DGD-PDA, and (e) D**YY**DGD-PDA pH 10 solution by AFM.

Table S3. Calculated *p*-values from the *t*-tests performed on Figure 7a data set.

Comparison	*p*-value	Comparison	*p*-value
**GG** pH 2 vs **GG** pH 10	$p < 0.01$	**VV** pH 2 vs **VV** pH 10	$p < 0.01$
**TT** pH 2 vs **TT** pH 10	$p < 0.05$	**FF** pH 2 vs **FF** pH 10	$p < 0.01$
**YY** pH 2 vs **YY** pH 10	n.s.	**GG** pH 2 vs **VV** pH 2	$p < 0.01$
**GG** pH 2 vs **TT** pH 2	n.s.	**GG** pH 2 vs **FF** pH 2	$p < 0.05$
**GG** pH 2 vs **YY** pH 2	$p < 0.01$	**VV** pH 2 vs **TT** pH 2	n.s.
**VV** pH 2 vs **FF** pH 2	$p < 0.01$	**VV** pH 2 vs **YY** pH 2	$p < 0.01$
**TT** pH 2 vs **FF** pH 2	$p < 0.05$	**TT** pH 2 vs **YY** pH 2	$p < 0.05$
**FF** pH 2 vs **YY** pH 2	$p < 0.01$	**GG** pH 10 vs **VV** pH 10	$p < 0.05$
**GG** pH 10 vs **TT** pH 10	$p < 0.05$	**GG** pH 10 vs **FF** pH 10	n.s.
**GG** pH 10 vs **YY** pH 10	n.s.	**VV** pH 10 vs **TT** pH 10	n.s.
**VV** pH 10 vs **FF** pH 10	$p < 0.01$	**VV** pH 10 vs **YY** pH 10	$p < 0.01$
**TT** pH 10 vs **FF** pH 10	n.s.	**TT** pH 10 vs **YY** pH 10	$p < 0.05$
**FF** pH 10 vs **YY** pH 10	$p < 0.05$		

$^{*}p< 0.05$; $^{**}p< 0.01$; $^{***}p< 0.001$; $^{****}p< 0.0001$; n.s. (not significant; $p>0.05$).

Table S4. Calculated *p*-values from the *t*-tests performed on Figure 7b data set.

Comparison	*p*-value	Comparison	*p*-value
**GG** pH 2 vs **GG** pH 10	$p < 0.0001$	**VV** pH 2 vs **VV** pH 10	$p < 0.0001$
**TT** pH 2 vs **TT** pH 10	$p < 0.0001$	**FF** pH 2 vs **FF** pH 10	$p < 0.05$
**YY** pH 2 vs **YY** pH 10	$p < 0.001$	**GG** pH 2 vs **VV** pH 2	n.s.
**GG** pH 2 vs **TT** pH 2	$p < 0.0001$	**GG** pH 2 vs **FF** pH 2	$p < 0.0001$
**GG** pH 2 vs **YY** pH 2	$p < 0.0001$	**VV** pH 2 vs **TT** pH 2	$p < 0.0001$
**VV** pH 2 vs **FF** pH 2	n.s.	**VV** pH 2 vs **YY** pH 2	$p < 0.0001$
**TT** pH 2 vs **FF** pH 2	$p < 0.05$	**TT** pH 2 vs **YY** pH 2	$p < 0.0001$
**FF** pH 2 vs **YY** pH 2	$p < 0.01$	**GG** pH 10 vs **VV** pH 10	$p < 0.05$
**GG** pH 10 vs **TT** pH 10	$p < 0.05$	**GG** pH 10 vs **FF** pH 10	$p < 0.01$
**GG** pH 10 vs **YY** pH 10	$p < 0.01$	**VV** pH 10 vs **TT** pH 10	$p < 0.0001$
**VV** pH 10 vs **FF** pH 10	$p < 0.0001$	**VV** pH 10 vs **YY** pH 10	$p < 0.0001$
**TT** pH 10 vs **FF** pH 10	$p < 0.0001$	**TT** pH 10 vs **YY** pH 10	$p < 0.0001$
**FF** pH 10 vs **YY** pH 10	$p < 0.0001$		

*p< 0.05; **p< 0.01; ***p< 0.001; ****p< 0.0001; n.s. (not significant; p>0.05).

Optical properties of dried peptide-PDA films

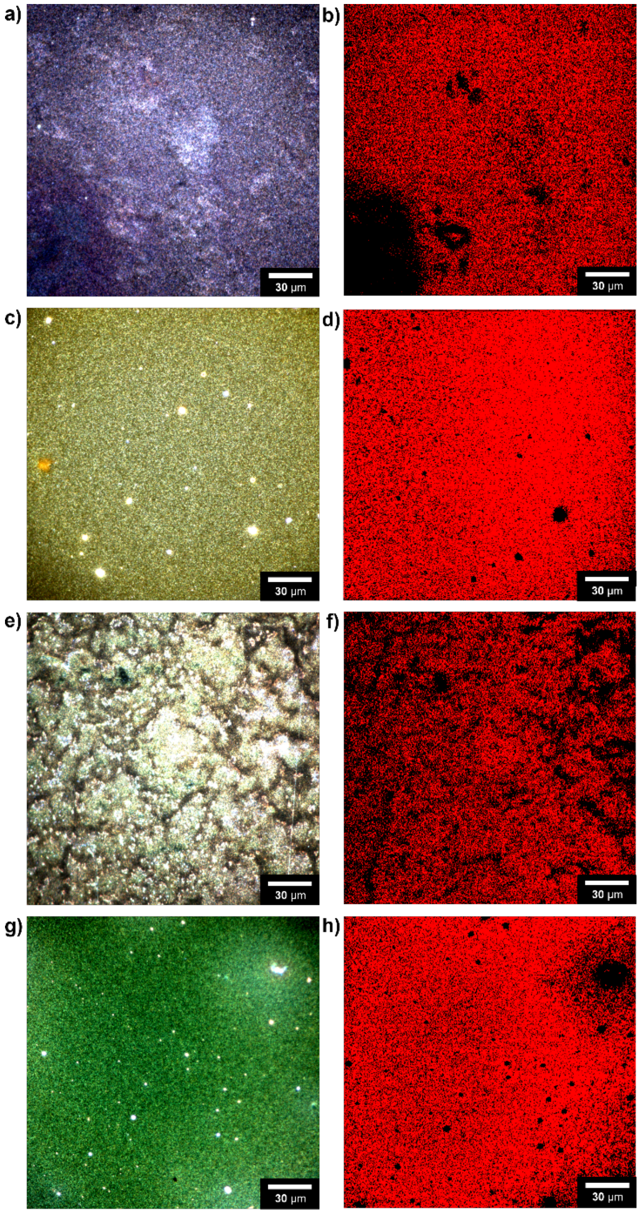

Figure S62. Darkfield images and SAM result of dried peptide-PDA films. Darkfield images of film made from (a) 1 mM DDGD**GG**-PDA pH 2 solution, (c) 1 mM DDGD**GG**-PDA pH 10 solution, (e) 1 mM DDGD**VV**-PDA pH 2 solution, and (g) 1 mM DDGD**VV**-PDA pH 10 solution. SAM (0.1R) images of film made from (b) 1 mM DDGD**GG**-PDA pH 2 solution, (d) 1 mM DDGD**GG**-PDA pH 10 solution, (f) 1 mM DDGD**VV**-PDA pH 2 solution, and (h) 1 mM DDGD**VV**-PDA pH 10 solution. Scale bar = 30 μm.

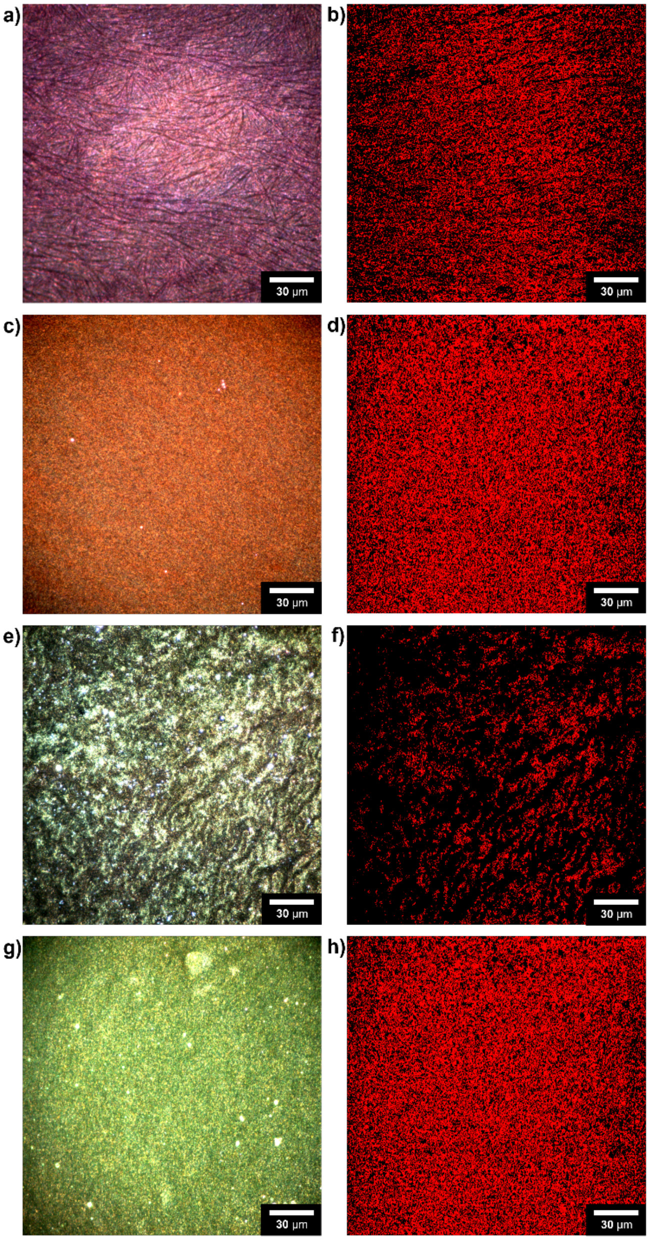

Figure S63. Darkfield images and SAM result of dried peptide-PDA films. Darkfield images of film made from (a) 1 mM DDGD**TT**-PDA pH 2 solution, (c) 1 mM DDGD**TT**-PDA pH 10 solution, (e) 1 mM DDGD**FF**-PDA pH 2 solution, and (g) 1 mM DDGD**FF**-PDA pH 10 solution. SAM (0.1R) images of film made from (b) 1 mM DDGD**TT**-PDA pH 2 solution, (d) 1 mM DDGD**TT**-PDA pH 10 solution, (f) 1 mM DDGD**FF**-PDA pH 2 solution, and (h) 1 mM DDGD**FF**-PDA pH 10 solution. Scale bar = 30 μm.

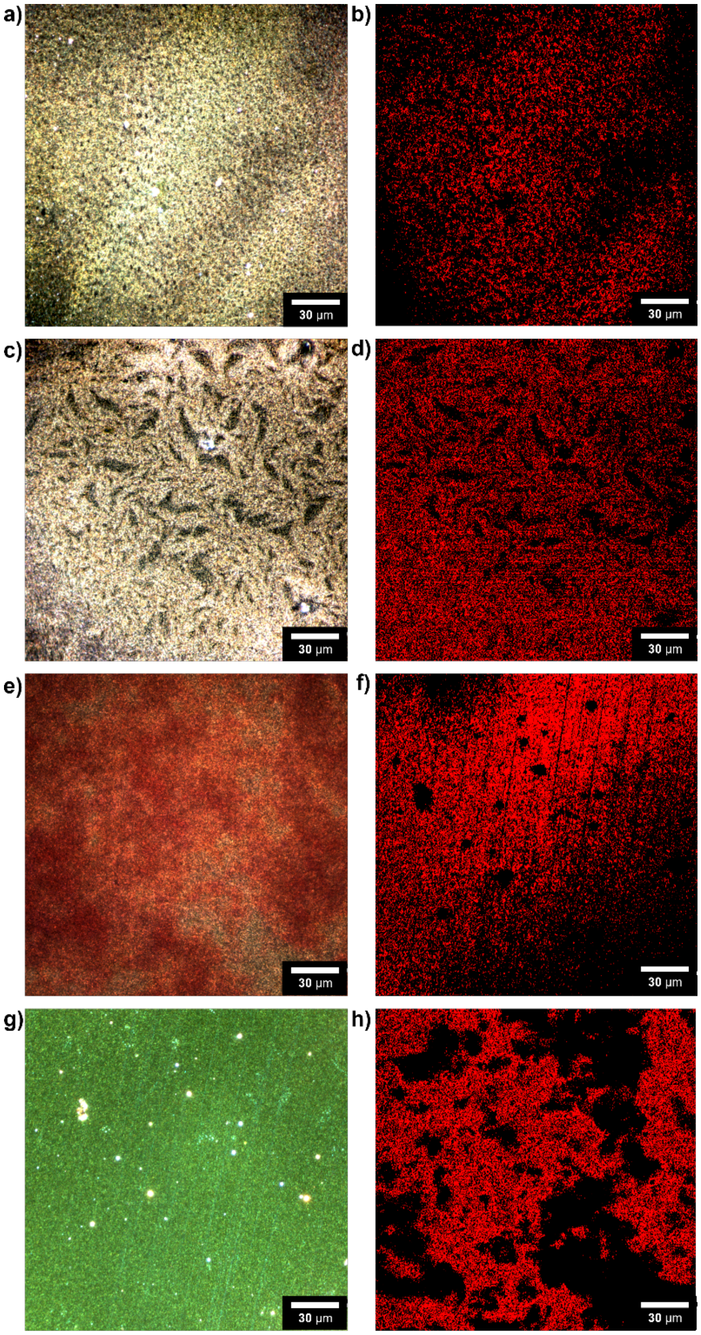

Figure S64. Darkfield images of film made from (a) 1 mM DDGD**YY**-PDA pH 2 solution, (c) 1 mM DDGD**YY**-PDA pH 10 solution, (e) 1 mM D**GG**DGD-PDA pH 2 solution, and (g) 1 mM D**GG**DGD-PDA pH 10 solution. SAM (0.1R) images of film made from (b) 1 mM DDGD**YY**-PDA pH 2 solution, (d) 1 mM DDGD**YY**-PDA pH 10 solution, (f) 1 mM D**GG**DGD-PDA pH 2 solution, and (h) 1 mM D**GG**DGD-PDA pH 10 solution. Scale bar = 30 µm.

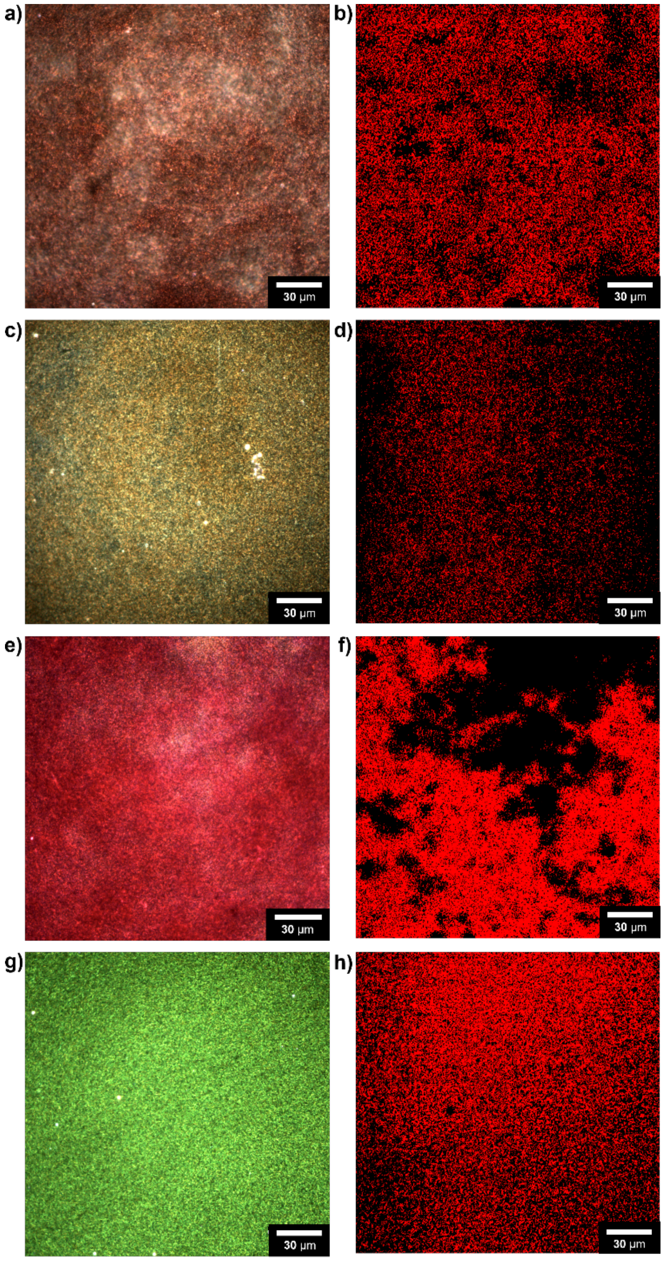

Figure S65. Darkfield images and SAM result of dried peptide-PDA films. Darkfield images of film made from (a) 1 mM D**VV**DGD-PDA pH 2 solution, (c) 1 mM D**VV**DGD-PDA pH 10 solution, (e) 1 mM D**TT**DGD-PDA pH 2 solution, and (g) 1 mM D**TT**DGD-PDA pH 10 solution. SAM (0.1R) images of film made from (b) 1 mM D**VV**DGD-PDA pH 2 solution, (d) 1 mM D**VV**DGD-PDA pH 10 solution, (f) 1 mM D**TT**DGD-PDA pH 2 solution, and (h) 1 mM DTTDGD-PDA pH 10 solution. Scale bar = 30 μm.

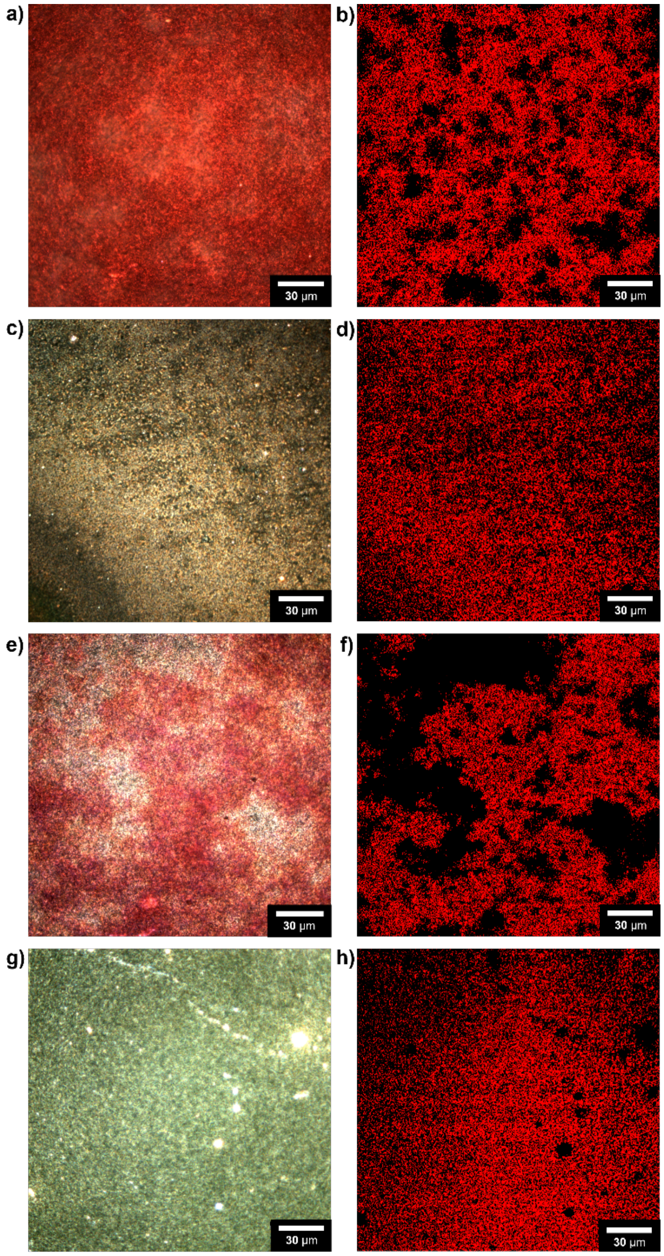

Figure S66. Darkfield images and SAM result of dried peptide-PDA films. Darkfield images of film made from (a) 1 mM D**FF**DGD-PDA pH 2 solution, (c) 1 mM D**FF**DGD-PDA pH 10 solution, (e) 1 mM D**YY**DGD-PDA pH 2 solution, and (g) 1 mM D**YY**DGD-PDA pH 10 solution. SAM (0.1R) images of film made from (b) 1 mM D**FF**DGD-PDA pH 2 solution, (d) 1 mM D**FF**DGD-PDA pH 10 solution, (f) 1 mM D**YY**DGD-PDA pH 2 solution, and (h) 1 mM D**YY**DGD-PDA pH 10 solution. Scale bar = 30 µm.

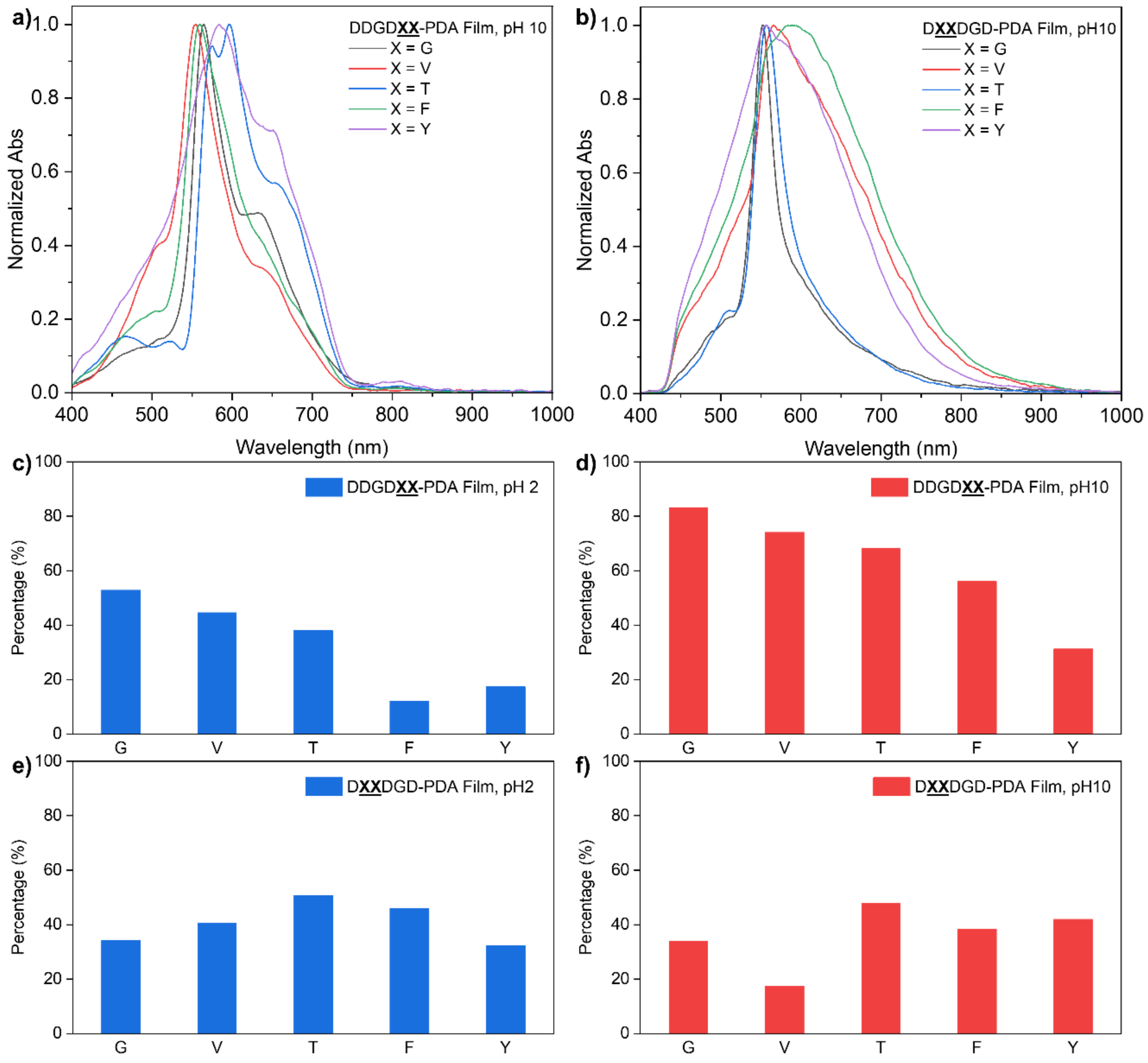

Figure S67. Optical properties of peptide-PDA dried films. Absorption spectra of film made from (a) DDGD**<u>XX</u>**-PDA) and (b) D**<u>XX</u>**DGD-PDA (X = G, V, T, F, and Y) solution (pH 10) with the highest matching percentage based on SAM (0.1 R). Distribution of the highest matching percentage based on SAM within the chosen location of the film made from (c, d) D**<u>XX</u>**DGD-PDA and (e,f) DDGD**<u>XX</u>**-PDA (X = G, V, T, F, and Y) solutions at (c,e) pH 2 and (d,f) pH 10.

Interfacing peptide-PDA monomers and their polymerized films with HDFs.

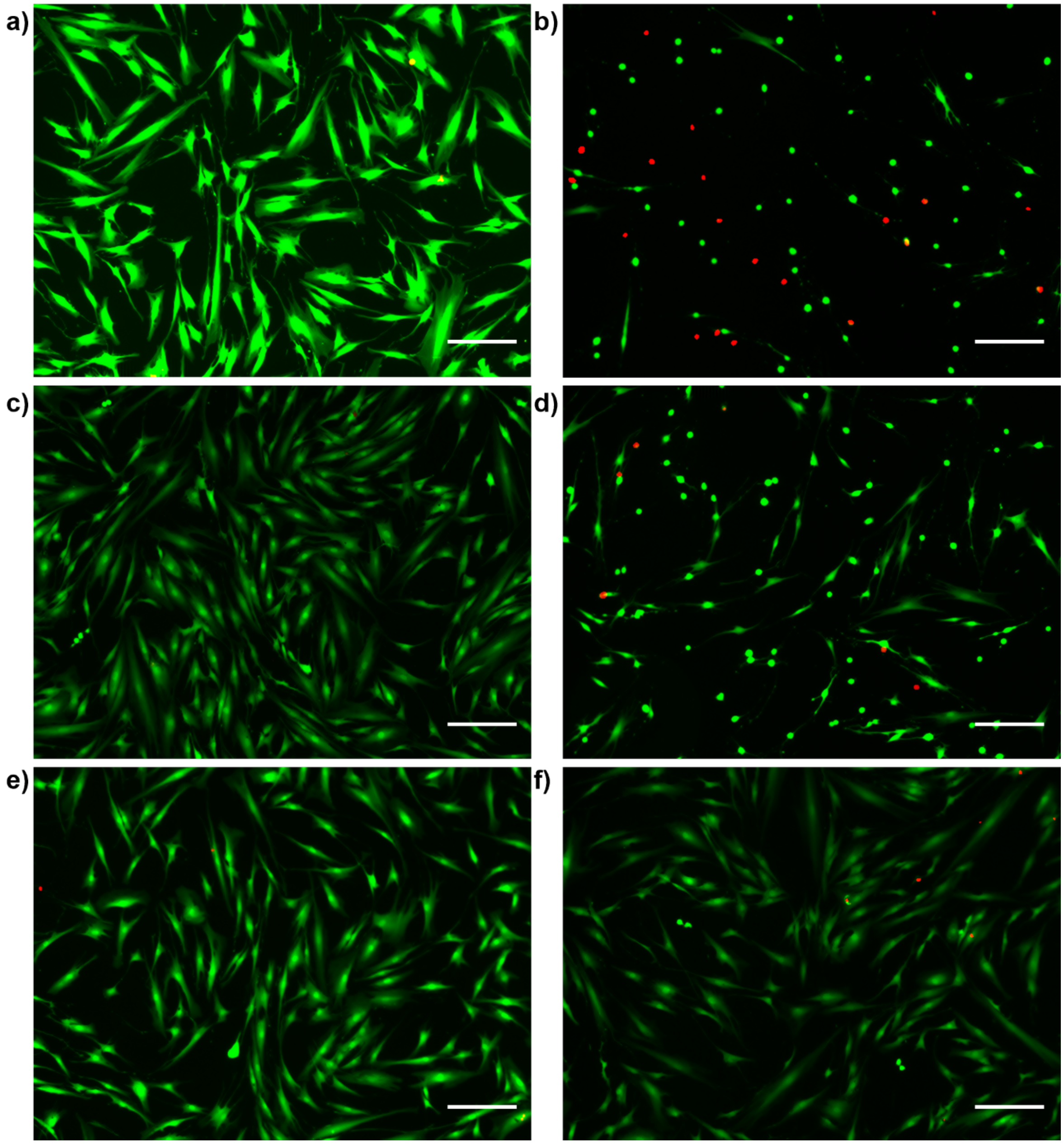

Figure S68. Representative fluorescence images of DDGD**XX**-DA-exposed HDFs subjected to LIVE/DEAD assay. (a) Positive control (without peptide-DA incubation). 6 h incubation with 1 mM (b) DDGD**GG**-DA, (c) DDGD**VV**-DA, (d) DDGD**TT**-DA, (e) DDGD**FF**-DA, and (f) DDGD**YY**-DA under cell culture conditions. Scale bar = 200 μm.

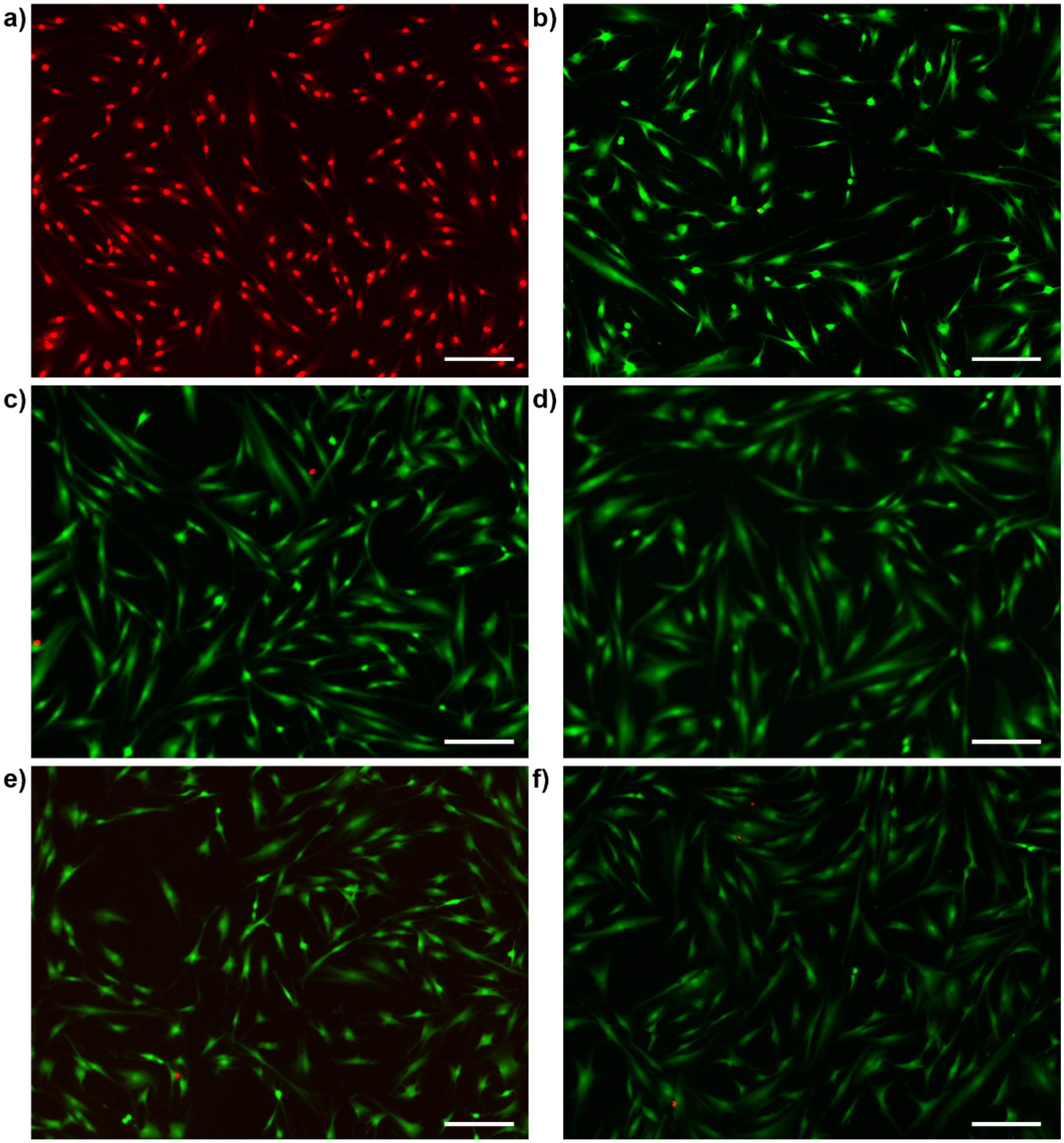

Figure S69. Representative fluorescence images of D**XX**DGD-DA-exposed HDFs subjected to LIVE/DEAD assay. (a) Negative control (30 min incubation with methanol). 6h incubation with 1 mM (b) D**GG**DGD-DA, (c) D**VV**DGD-DA, (d) D**TT**DGD-DA, (e) D**FF**DGD-DA, and (f) D**YY**DGD-DA under cell culture conditions. Scale bar = 200 μm.

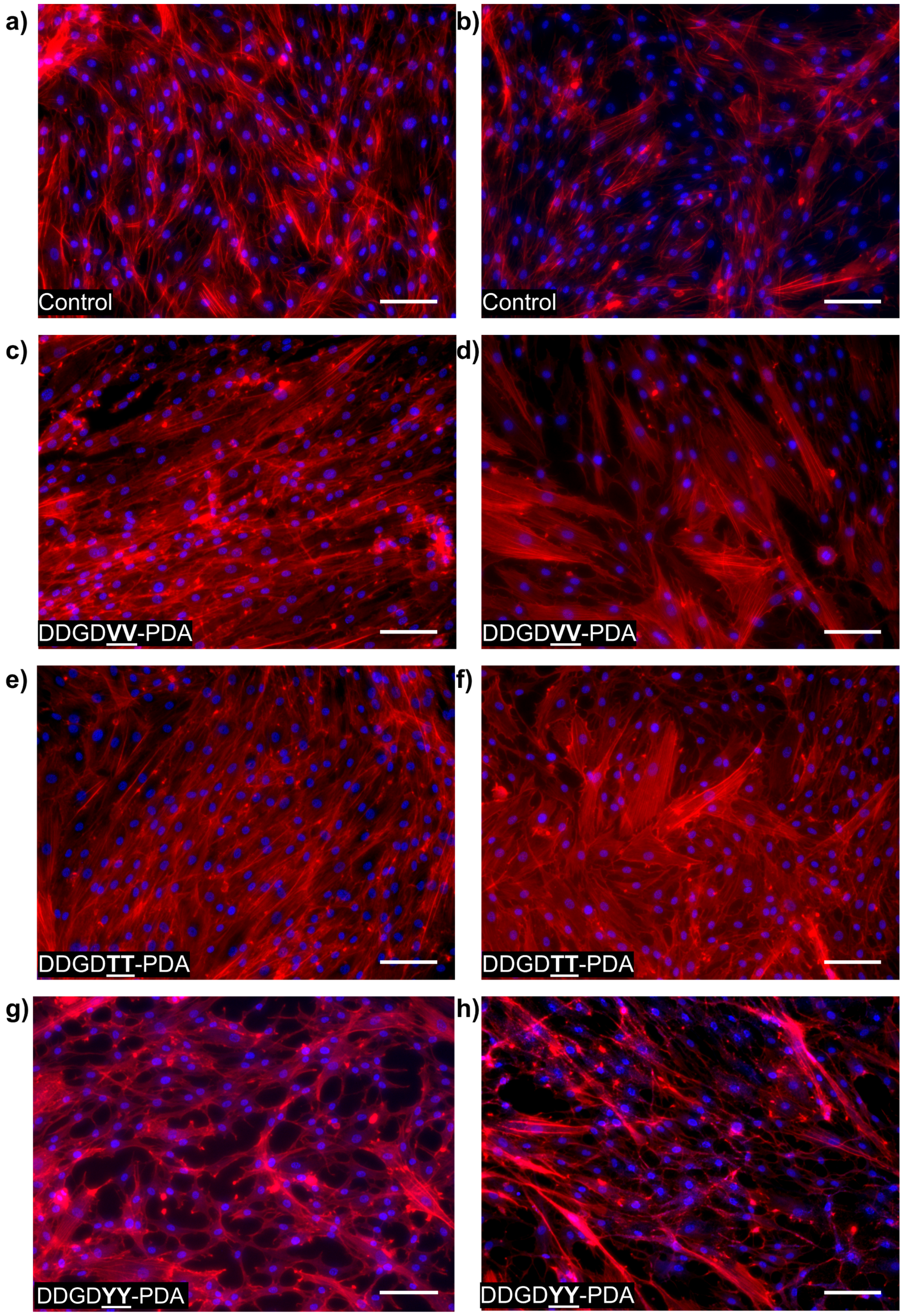

Figure S70. Representative images of HDFs incubated for 5 days on films made from 5 mM peptide-PDA solutions at pH 2; HDFs on (a)-(b) glass coverslips and 5 mM (c)-(d) DDGD**VV**-PDA, (e)-(f) 5 mM DDGD**TT**-PDA, and (g)-(h) 5 mM DDGD**YY**-PDA. Cells were stained with **DAPI** (nucleus) and **Phalloidin** (F-actin); Scale bar = 100 µm.

References

(1) Diegelmann, S. R.; Hartman, N.; Markovic, N.; Tovar, J. D. Synthesis and Alignment of Discrete Polydiacetylene-Peptide Nanostructures. *J Am Chem Soc* **2012**, *134* (4), 2028–2031. https://doi.org/10.1021/ja211539j.

(2) Neese, F. The ORCA Program System. *Wiley Interdiscip Rev Comput Mol Sci* **2012**, *2* (1), 73–78. https://doi.org/10.1002/wcms.81.

(3) Grimme, S.; Bannwarth, C.; Shushkov, P. A Robust and Accurate Tight-Binding Quantum Chemical Method for Structures, Vibrational Frequencies, and Noncovalent Interactions of Large Molecular Systems Parametrized for All Spd-Block Elements (Z = 1-86). *J Chem Theory Comput* **2017**, *13* (5), 1989–2009. https://doi.org/10.1021/acs.jctc.7b00118.

(4) Liu, Z.; Lu, T.; Chen, Q. Intermolecular Interaction Characteristics of the All-Carboatomic Ring, Cyclo[18]Carbon: Focusing on Molecular Adsorption and Stacking. *Carbon N Y* **2021**, *171*, 514–523. https://doi.org/10.1016/j.carbon.2020.09.048.

(5) Lu, T.; Chen, F. Multiwfn: A Multifunctional Wavefunction Analyzer. *J Comput Chem* **2012**, *33* (5), 580–592. https://doi.org/10.1002/jcc.22885.

(6) Jorgensen, W. L.; Maxwell, D. S.; Tirado-Rives, J. Development and Testing of the OPLS All-Atom Force Field on Conformational Energetics and Properties of Organic Liquids. *J Am Chem Soc* **1996**, *118* (45), 11225–11236. https://doi.org/10.1021/ja9621760.

(7) Bowers, K. J.; Sacerdoti, F. D.; Salmon, J. K.; Shan, Y.; Shaw, D. E.; Chow, E.; Xu, H.; Dror, R. O.; Eastwood, M. P.; Gregersen, B. A.; Klepeis, J. L.; Kolossvary, I.; Moraes, M. A. Molecular Dynamics---Scalable Algorithms for Molecular Dynamics Simulations on Commodity Clusters. In *Proceedings of the 2006 ACM/IEEE conference on Supercomputing - SC '06*; ACM Press: New York, New York, USA, 2006; p 84. https://doi.org/10.1145/1188455.1188544.

(8) MacRae, C. F.; Sovago, I.; Cottrell, S. J.; Galek, P. T. A. A.; McCabe, P.; Pidcock, E.; Platings, M.; Shields, G. P.; Stevens, J. S.; Towler, M.; Wood, P. A. Mercury 4.0 : From Visualization to Analysis, Design and Prediction. *J Appl Crystallogr* **2020**, *53* (1), 226–235. https://doi.org/10.1107/S1600576719014092.

(9) Humphrey, W.; Dalke, A.; Schulten, K. VMD: Visual Molecular Dynamics. *J Mol Graph* **1996**, *14* (1), 33–38. https://doi.org/10.1016/0263-7855(96)00018-5.

(10) Steiner, T.; Thomas, S. The Hydrogen Bond in the Solid State. *Angewandte Chemie International Edition* **2002**, *41* (1), 48–76. https://doi.org/10.1002/1521-3773(20020104)41:1<48::AID-ANIE48>3.0.CO;2-U.
